# Targeting transglutaminase 2 mediated exostosin glycosyltransferase 1 signaling in liver cancer stem cells with acyclic retinoid

**DOI:** 10.1038/s41419-023-05847-4

**Published:** 2023-06-13

**Authors:** Xian-Yang Qin, Yutaka Furutani, Kento Yonezawa, Nobutaka Shimizu, Miyuki Kato-Murayama, Mikako Shirouzu, Yali Xu, Yumiko Yamano, Akimori Wada, Luc Gailhouste, Rajan Shrestha, Masataka Takahashi, Jeffrey W. Keillor, Ting Su, Wenkui Yu, Shinya Fujii, Hiroyuki Kagechika, Naoshi Dohmae, Yohei Shirakami, Masahito Shimizu, Takahiro Masaki, Tomokazu Matsuura, Harukazu Suzuki, Soichi Kojima

**Affiliations:** 1 Laboratory for Cellular Function Conversion Technology, RIKEN Center for Integrative Medical Sciences, Yokohama, Japan; 2 Liver Cancer Prevention Research Unit, RIKEN Cluster for Pioneering Research, Wako, Saitama, Japan; 3 Department of Laboratory Medicine, The Jikei University School of Medicine, Tokyo, Japan; 4 Photon Factory, Institute of Materials Structure Science, High Energy Accelerator Research Organization (KEK), Tsukuba, Ibaraki Japan; 5 Center for Digital Green-innovation, Nara Institute of Science and Technology, Takayama, Ikoma, Nara, Japan; 6 Laboratory for Protein Functional and Structural Biology, RIKEN Center for Biosystems Dynamics Research, Yokohama, Kanagawa Japan; 7 School of Medicine, Nanjing University, Nanjing, Jiangsu China; 8 Laboratory of Organic Chemistry for Life Science, Kobe Pharmaceutical University, Kobe, Hyogo Japan; 9 Laboratory for Brain Development and Disorders, RIKEN Center for Brain Science, Saitama, Japan; 10 Department of Pharmacy, Kathmandu University, Dhulikhel, Kavre Nepal; 11 Department of Chemistry and Biomolecular Sciences, University of Ottawa, Ottawa, ON Canada; 12 Institute of Biomaterials and Bioengineering, Tokyo Medical and Dental University, Tokyo, Japan; 13 Biomolecular Characterization Unit, RIKEN Center for Sustainable Resource Science, Wako, Saitama Japan; 14 Department of Gastroenterology, Graduate School of Medicine, Gifu University, Gifu, Japan

**Keywords:** Mechanism of action, Liver cancer, SAXS, Cancer stem cells

## Abstract

Transglutaminase 2 (TG2) is a multifunctional protein that promotes or suppresses tumorigenesis, depending on intracellular location and conformational structure. Acyclic retinoid (ACR) is an orally administered vitamin A derivative that prevents hepatocellular carcinoma (HCC) recurrence by targeting liver cancer stem cells (CSCs). In this study, we examined the subcellular location-dependent effects of ACR on TG2 activity at a structural level and characterized the functional role of TG2 and its downstream molecular mechanism in the selective depletion of liver CSCs. A binding assay with high-performance magnetic nanobeads and structural dynamic analysis with native gel electrophoresis and size-exclusion chromatography-coupled multi-angle light scattering or small-angle X-ray scattering showed that ACR binds directly to TG2, induces oligomer formation of TG2, and inhibits the transamidase activity of cytoplasmic TG2 in HCC cells. The loss-of-function of TG2 suppressed the expression of stemness-related genes, spheroid proliferation and selectively induced cell death in an EpCAM+ liver CSC subpopulation in HCC cells. Proteome analysis revealed that TG2 inhibition suppressed the gene and protein expression of exostosin glycosyltransferase 1 (EXT1) and heparan sulfate biosynthesis in HCC cells. In contrast, high levels of ACR increased intracellular Ca^2+^ concentrations along with an increase in apoptotic cells, which probably contributed to the enhanced transamidase activity of nuclear TG2. This study demonstrates that ACR could act as a novel TG2 inhibitor; TG2-mediated EXT1 signaling is a promising therapeutic target in the prevention of HCC by disrupting liver CSCs.

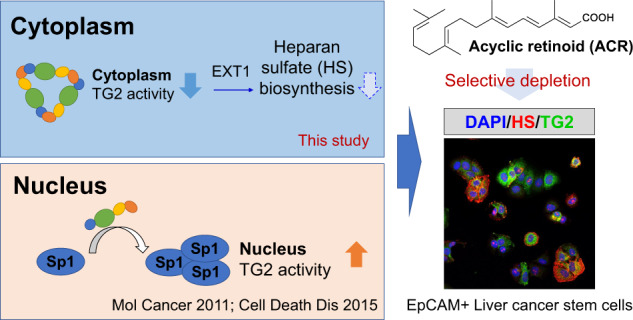

## Introduction

Liver cancer is a severe and fatal form of cancer, with ~830,180 deaths worldwide in 2020 [1]. Hepatocellular carcinoma (HCC) is the most common type of liver cancer, accounting for ~75–85% of cases [2]. Although considerable advances have been made in the treatment of patients with unresectable HCC using multikinase inhibitors [3, 4] or immuno-oncological therapies [5], these systemic therapies are often ineffective and prone to adverse events [6]. According to lineage tracing technology, HCC cells originate from the differentiated mature hepatocytes [7, 8]. Hepatocytes are highly plastic and can dedifferentiate into stem-like cells under stress [7, 9]. In this context they could behave as cancer stem cells (CSCs) and contribute to hepatocarcinogenesis; thus CSCs could serve as targets for preventative HCC therapies [10].

Acyclic retinoid (ACR; also known as all-*trans*-3,7,11,15-tetramethyl-2,4,6,10,14-hexadecapentaenoic acid, peretinoin, and NIK-333) was originally developed as an orally administered vitamin A derivative with high binding affinity with cellular retinoic acid-binding protein (CRABP) equal to that of all-*trans* retinoic acid (ATRA) [11]. Similarly to retinoic acids, ACR functions as an agonist for both nuclear retinoic acid receptors (RARs) and retinoid X receptors (RXRs) [12, 13] and activates their respective DNA-response elements (RXRE or RARE) and intracellular retinoid signaling. Animal studies showed that ACR exhibited substantial chemopreventive activity against chemically induced or metabolic syndrome-associated HCC, such as in diethylnitrosamine (DEN) or 3′-methyl-4-dimethylaminoazobenzene-induced rat liver tumorigenesis [14, 15], in DEN-induced liver tumorigenesis in obese and diabetic mice [16], in platelet-derived growth factor C overexpression transgenic mice [17], in atherogenic high-fat diet-fed mice and stelic animal model (STAM) mice [18], and very recently in murine double minute 2 depletion mice [19]. In line with these animal studies, several clinical studies have effectively applied ACR to prevent the recurrence of HCC after curative treatment [20, 21], partly by targeting MYCN-positive liver CSCs [22]. However, a recent phase 3 trial with a specific focus on patients classified as Child-Pugh A showed no statistic superiority of ACR in inhibiting the recurrence of HCC (NCT01640808), highlighting a need of future efforts to understand the molecular mechanism of ACR in liver CSCs.

Transglutaminase 2 (TG2) is the most widely distributed member of the transglutaminase family and is a multifunctional protein that promotes or suppresses tumorigenesis depending on the cell context [23], i.e., TG2 promotes cell proliferation or cell death based on its intracellular location and conformational structure [24]. Intracellular TG2 is predominantly found in the cytosol, where it transitions to a compact and closed conformation that can bind GTP and function as a G-protein [25]. The G-protein signaling is required for TG2-dependent cancer stem cell invasion, migration, and tumor formation in epidermal squamous cell carcinoma [26], colorectal cancer [27], and breast cancer [28]. These processes are generated partly by regulating epithelial-mesenchymal transition (EMT), which is critical for maintaining epithelial plasticity [29, 30]. In contrast, the binding of Ca^2+^ stabilizes the open conformation of TG2 and exposes the catalytic site of its transamidase activity [31]. Our previous studies showed that increased transamidase activity of nuclear TG2 contributes to alcohol [32], lipid [33], or microbiome-induced [34, 35] apoptosis of normal hepatocytes, and ACR-induced apoptosis in HCC cells [36]. ACR accelerated the formation of a trimeric complex of TG2 with importin-α/β, which might contribute to the nuclear localization of TG2 [37]. However, it has not been clear whether ACR binds to TG2 and directly affects its transamidase activity.

Although TG2 is well-known for its function in the maintenance of CSC-like cell survival in diverse cancers such as breast cancer [38], epidermal squamous cell carcinoma [39], renal cell carcinoma [40], ovarian cancer [41], and colon cancer [42], little is known about its role in liver CSCs [43]. In contrast, two independent clinical studies showed that the tissue and/or serum TG2 expression were significantly increased in patients with early stage HCC whose with no evaluated level of AFP [44] or with early recurrence within 12 months after primary treatment [45]. Therefore, there is a concern about the oncogenic function and molecular mechanism of TG2 in hepatic tumorigenesis by promoting the stemness characteristics of liver CSCs. In this study, we described the subcellular location-dependent effects of ACR on TG2 activity at the structural level. We characterized the functional role of TG2 by using a binding assay with high-performance magnetic nanobeads and structural dynamics analysis and elucidated its downstream molecular mechanism in the selective depletion of liver CSCs using proteome analysis. Our data showed that ACR directly binds to and induces oligomer formation of TG2, which inhibits the transamidase activity of cytoplasmic TG2 in HCC cells. The loss-of-function of TG2 selectively induced cell death in the liver CSC subpopulation in HCC cells, accompanying by suppressing the gene and/or protein expression of enzymes involved in cell recognition and membrane protein regulation such as exostosin glycosyltransferase 1 (EXT1) and cellular lipid composition and heparan sulfate (HS) biosynthesis. We propose that novel TG2 inhibitors should be further evaluated as a potential therapeutic approach in the prevention of HCC by targeting liver CSCs.

## Materials and methods

### Reagents

Magnetic ferrite glycidyl methacrylate (FG) beads with *N*-hydroxysuccinimide (NHS) functional groups (NHS beads; TAS8848 N1141) were purchased from Tamagawa Seiki (Nagano, Japan). ACR and its methyl derivatives, ACR-23 and ACR-55, were supplied by Kowa (Tokyo, Japan). H_2_N-ACR was synthesized from commercially available farnesyl acetate (Fig. S1). The vitamin K2 analog, SVK30, was synthesized by S.F. and H.K. [46]. The irreversible TG2 inhibitor, NC9, was synthesized by J.W.K. [47]. Recombinant human TG2 (T002) and the irreversible TG2 inhibitor, Z-DON-Val-Pro-Leu-OMe (ZDON, Z006), were purchased from Zedira (Darmstadt, Germany). A TG2 inhibitor cystamine (CTM; M6500), lipopolysaccharide (LPS; Escherichia coli O111:B4), 2,5-dihydroxybenzoic acid (DHB; 149357), trans-2-[3-(4-tert-butylphenyl)-2-methyl-2-propenylidene]malononitrile (DCTB; 727881), cesium triiodide (483338), acetonitrile (271004), and trifluoroacetic acid (A2889) were purchased from Sigma-Aldrich (St. Louis, MO, USA). A biotinylated substrate for TG2, EZ-Link™ Pentylamine-Biotin (5BAPA [48, 49]), was purchased from Thermo Fisher Scientific (21345; Rockford, IL, USA). *N,N*-Dimethylformamide (DMF), dimethyl sulfoxide (DMSO), phosphate-buffered saline solution (PBS), or ethanol (EtOH) were used as controls.

### ACR binding assays

DMF and H_2_N-ACR-immobilized FG beads were prepared by Tamagawa Seiki (Tokyo, Japan). Recombinant human TG2 (100 ng) was incubated on a rotator at 4 °C overnight with 5 µL FG beads immobilized with 0, 0.1, 0.3, or 1 mM H_2_N-ACR in the absence or presence of 50 nmol free ACR. Beads were extensively washed with Tris buffer (pH 7.8) and heated for 5 min at 97 °C. The boiled and eluted sample was loaded onto sodium dodecyl sulfate-polyacrylamide gel electrophoresis (SDS-PAGE) and transferred to a PVDF membrane (Bio-Rad Laboratories, Hercules, CA, USA). After blocking, the membrane was probed with mouse anti-TG2 antibody (1:3,000 dilution, MA5-12739, Thermo Fisher Scientific) overnight at 4 °C. The resultant blots were incubated with HRP-conjugated secondary antibodies (1:2000 dilution) and detected using an Amersham ECL Plus western blotting Detection System (GE Healthcare, Piscataway, NJ, USA). Band intensities were quantified using the ImageJ software (National Institutes of Health, USA).

### Expression and purification of recombinant human TG2

Recombinant human TG2 protein was expressed and purified as previously described [50]. Briefly, the TG2 gene, with an *N*-terminal histidine tag, was subcloned into the plasmid pCR2.1-TOPO (Thermo Fisher Scientific) and expressed in an *E. coli* KRX strain. The cells were sonicated in lysis buffer (50 mM sodium phosphate [pH 7.5], 400 mM NaCl, 5 mM 2-mercaptoethanol, 1 mM PMSF, 50 µM GTP, 50 µM ATP and 0.5% Triton X-100). TG2 was purified using a HisTrap column, HiTrap Q column and HiLoad 16/600 Superdex 200 gel filtration column (Cytiva, Tokyo, Japan). TG2 was concentrated in 50 mM HEPES (pH 7.0), 100 mM NaCl, 1 mM EDTA, 5 mM DTT, and 5% glycerol, to a final concentration of 5.6 mg/mL.

### SEC-MALS

Size-exclusion chromatography coupled with multi-angle light scattering (SEC-MALS) was performed with a high-performance liquid chromatography system (HPLC) (Alliance 2695, Waters, Milford, MA, USA) and MALS system (DAWN HELEOS II, Wyatt Technology, Santa Barbara, CA, USA) [51]. A refractive index detector (2414, Waters) was also installed in this system for the estimation of a sample concentration. The recombinant human TG2 (5.6 mg/mL) was incubated with EtOH, 100 μM GTP, 100 μM ACR or 100 μM ACR in the presence of 100 μM GTP for 2 h at room temperature. Superdex 200 Increase 10/300 GL (Cytiva, Uppsala, Sweden) was equilibrated with 50 mM HEPES (pH 7.0), 100 mM NaCl, 1 mM EDTA, 5 mM DTT, 5% glycerol was used as the gel filtration column, and the flow rate and the injection volume were 0.5 mL/min and 0.03 mL, respectively. SEC-MALS/RI data were analyzed by ASTRA 6.1 (Wyatt Technology) and image processing was carried out using Igor pro (WaveMetrics, Portland, OR, USA).

### SEC-SAXS

The small-angle X-ray scattering coupled with a size-exclusion chromatography (SEC-SAXS) was performed at the BL-10C beamline at the Photon Factory of the High Energy Accelerator Research Organization (KEK) in Tsukuba, Japan [52]. The X-ray wavelength and the distance between the sample and the detector were 1.213 Å and 2008 mm, respectively. The X-ray scattering intensity was measured using PILATUS3 2 M detector (DECTRIS AG, Baden, Switzerland). The sample cell for SAXS with an optical path length of 1.0 mm was made of stainless steel with a 1.5-mm vertical × 3.0-mm horizontal window covered with quartz glass of 0.02 mm thickness. The UV-visible absorption spectra were measured by a fiber spectrometer (QEpro, Ocean Insight, Largo, FL, USA) with the same sample cell as SAXS [51]. The fiber optics were placed at a 45-degree angle to the X-rays, and a UV-visible fiber light source (L10290, Hamamatsu Photonics K.K., Shizuoka, Japan) was used as the light source for the D2 lamp. The temperature of the sample cell was kept at 293 K. ACQUITY UPLC H-Class (Waters) and Superdex 200 increase 10/300 (Cytiva) were used as a HPLC system and a gel filtration column for SEC, respectively. The column was equilibrated with the same buffer as in the SEC-MALS experiments. All the prepared sample conditions for SEC-SAXS are identical to those for SEC-MALS. The sample volume for each injection was 0.15 mL. The elution rate was initially 0.5 mL/min and changed to 0.05 mL/min around the peak. An exposure time of 20 s was set for SAXS continuous measurement. The UV-visible absorption spectra were measured every 10 s with an integration time of 1 s. The recorded two-dimensional scattering intensity data were converted to one-dimensional scattering intensity data by azimuthal averaging. The background buffer data were acquired by averaging 20 data points before sample measurement, and all the background-subtracted scattering intensities were calibrated to absolute intensity scale by the water scattering intensity. These processes were performed with SAngler [53]. Data on the ascent side of the main elution peak were used predominantly in the analysis, and the scattering curve extrapolated to zero concentration were computed with Serial Analyzer [54]. Guinier analysis was carried out using AUTORG [55], and dimensionless Kratky plots [56] were created based on the resulting values of the radius of gyration *R*_g_ and the forward scattering intensity *I*_(0)_. Theoretical scattering curves from the two crystal structures (PDB code: 1KV3, 2Q3Z) were calculated with CRYSOL [57]. OLIGOMER [58] was used to estimate the volume fractions of these two crystal structures in solution based on the experimental curves. The degree of difference between the theoretical curve and the experimental curve is displayed by the χ^2^ value. The results of the SAXS analysis are summarized in Table S1 and all the graphs related to SAXS analysis were generated by Igor pro (WaveMetrics). The extinction coefficients were calculated with ProtParam [59], and the values of partial specific volume and scattering contrast were calculated with MULCh [60], respectively.

### Native PAGE

Recombinant human TG2 (1 μg) was incubated with either 500 μM enzyme inhibitor GTP; 5 mM enzyme activator CaCl_2_; 100 μM ACR, ACR-23, ACR-55, or SVK30 in 50 mM HEPES (pH 7.0), 100 mM NaCl, 1 mM EDTA, 5 mM DTT, 5% glycerol for 1–2 h at room temperature and then subjected to electrophoresis using a non-denaturing gel (8–16% Mini-Protean TGX gel, Bio-Rad Laboratories). The protein bands were visualized using a silver staining kit (Wako Industries, Osaka, Japan).

### Cell culture

The HCC cell line JHH7 was supplied by T.M. The HCC cell line Huh7 was obtained from JCRB Cell Bank (Osaka, Japan). The cells were maintained in Dulbecco’s modified Eagle’s medium (Wako Industries) containing 10% fetal bovine serum (Mediatech, Herndon, VA, USA) with 100 U/mL penicillin/streptomycin and 2 mmol/L _L_-glutamine (Mediatech) and were grown at 37 °C in a humidified incubator under 5% CO_2_.

### Lentiviral-shRNA transduction

TG2 (sc-37514-v) and control (sc‑108080) short hairpin RNA (shRNA) lentiviral particles were obtained from Santa Cruz Biotechnology (Santa Cruz, CA, USA). The cells were seeded in a 12-well plate and cultured until they reached ~50% confluence. The cells were transduced with lentiviral vectors expressing the shRNAs at 1 multiplicity of infection (MOI) using 5 μg/mL polybrene (Santa Cruz Biotechnology) and selected with 2 μg/mL puromycin‑containing culture medium.

### siRNA transfection

A pool of three target-specific siRNAs targeting human EXT1 (sc-36464, siEXT1) and control siRNA (sc-37007, siCtl) were purchased from Santa Cruz Biotechnology. The cells were transfected with 100 nM siRNA using a Lipofectamine 2000 transfection reagent (Thermo Fisher Scientific) at a concentration of 1.25 μL/well in 48-well plates for PCR analysis and 0.25 μL/well in 96-well plates for cell viability assay in serum-free medium.

### Cell viability assay

Cell viability was measured using a Cell Counting Kit-8 (Dojindo Molecular Technologies, Tokyo, Japan) and absorbance at 450 nm was measured using an EnSight Multimode Plate Reader (Perkin Elmer, Waltham, MA, USA).

### Spheroid proliferation assays

In all, 3D spheroid cultures were performed on non-adherent 96-well round-bottomed Sumilon PrimeSurface plates (MS-9096U, Sumitomo Bakelite, Tokyo, Japan) at a concentration of 5000 cells per well. The spheroids were cultured for 3 or 7 days, and images were recorded using an optical microscope (ZEN, NIKON, Tokyo, Japan). Spheroid proliferation was measured using a CellTiter-Fluor Cell Viability Assay (Promega Corporation, Madison, WI, USA) in the EnSight Multimode Plate Reader (Perkin Elmer Inc.) as previously described [61].

### Flow cytometry and cell sorting

The expression of the liver CSC marker EpCAM was analyzed using a BD LSR flow cytometer and CellQuest Pro software (BD Biosciences, San Jose, CA, USA). The cells were labeled with Alexa Fluor 647-conjugated mouse anti-EpCAM (1:20, 324212, BioLegend, San Diego, CA, USA) in cell staining buffer (BioLegend) for 30 min at room temperature. The data were analyzed using FlowJo software (Tree Star, Inc., Ashland, OR, USA). For cell sorting by flow cytometry, EpCAM-positive and EpCAM-negative subpopulations were isolated from JHH7 cells with Alexa Fluor 488-conjugated mouse anti-EpCAM (1:20, 324209, BioLegend) on a BD FACSAria (BD Biosciences) as previously described [22].

### Western blotting

Cells or liver tissues were lysed using a radioimmunoprecipitation assay buffer. Protein concentration was measured with the Pierce BCA Protein Assay Kit (Thermo Fisher Scientific). After brief sonication and boiling at 95 °C for 5 min, the protein samples were run on a 5–20% gradient gel, e-PAGE (EHR-R520L, ATTO, Tokyo, Japan), and transferred to a PVDF membrane (Bio-Rad Laboratories). The membranes were blocked with 5% nonfat dry milk in PBS containing 0.1 % Tween-20 (PBST) and then probed overnight at 4 °C with rabbit anti-TG2 (1:200 dilution, RB-060-P0, Thermo Fisher Scientific), mouse anti-EXT1 (1:100 dilution, sc-515144, Santa Cruz Biotechnology), mouse anti-pan-cytokeratin (1:500 dilution, MA182041, Thermo Fisher Scientific), or rat anti-GAPDH (1:1000 dilution, 607902, BioLegend) antibodies. The blots were incubated with HRP-conjugated secondary antibodies (1:2000 dilution) and detected using the Amersham ECL Plus western blotting Detection System (GE Healthcare). Band intensities were quantified using ImageJ software (National Institutes of Health).

### TG2 transamidase activity assay

The TG2 transamidase activity assay was performed as previously described [62]. Briefly, 96-well plates were coated with 1 mg/mL *N,N*-dimethylated casein in PBS overnight at 4 °C. After washing with PBST, recombinant human TG2 (obtained from Zedira or in-house purified TG2) was incubated with 20 μM of the TG2 substrate 5BAPA (Thermo Fisher Scientific) and ACR or NC9 in the presence or absence of 5 mM CaCl_2_ in 50 mM HEPES (pH 7.0), 100 mM NaCl, 1 mM EDTA, 5 mM DTT, and 5% glycerol for 1 h at 37 °C. Then, TG2-mediated incorporation of 5BAPA was quenched by the addition of 50 mM EDTA. After washing with PBST, immobilized 5BAPA was detected with streptavidin-alkaline phosphatase (Jackson ImmunoResearch Laboratories, West Grove, PA, USA) reacting with *p*-nitrophenyl phosphate (Sigma-Aldrich). Absorbance at 405 nm was measured using an EnSight Multimode Plate Reader (Perkin Elmer Inc.).

### Measurement of intracellular TG2 transamidase activity

Intracellular TG2 transamidase activity was measured as previously described [34]. Briefly, the cells were seeded in Greiner 96-well microtiter plates (Greiner Bio-One, Kremsmuenter, Austria) and incubated with 0.2 mM 5BAPA and 0.1 mM aminoguanidine overnight at 37 °C. The cells were treated with increasing concentrations of ACR in serum-free media for 4 h and then fixed with a 10% formaldehyde solution, permeabilized, blocked, and stained with streptavidin-tetramethylrhodamine isothiocyanate (TRITC, Jackson ImmunoResearch Laboratories). Intracellular TG2 transamidase activity was then detected as a fluorescence signal from TRITC and analyzed using an ImageXpressMICRO High Content Screening System (Molecular Devices, Sunnyvale, CA, USA). Morphological analysis was performed using a Multi-Wavelength Cell Scoring Application Module in MetaXpress Image Analysis software (Molecular Devices).

### Intracellular Ca^2+^ measurement

The cells were seeded in Greiner 96-well microtiter plates (Greiner Bio-One) and treated with ACR for 4 h. Then, the cells were loaded with a calcium-sensitive fluorescent ratiometric Fura-2AM dye (Dojindo Molecular Technologies) for 30 min at 37 °C. The fluorescence intensities were determined by recording at 340- and 380-nm excitation and 510-nm emission with the EnSight Multimode Plate Reader (Perkin Elmer Inc.). The ratio of F340 to F380 (F340/F380) was used as a relative indicator of intracellular Ca^2+^ concentration.

### LPS experiment

All the animal experiments were performed in accordance with protocols approved by the Institutional Committee of Animal Experiment of RIKEN and adhered to the guidelines in the Institutional Regulations for Animal Experiments and Fundamental Guidelines for Proper Conduct of Animal Experiments and Related Activities in Academic Research Institutions under the jurisdiction of the Ministry of Education, Culture, Sports, Science and Technology, Japan. Male C57BL6/J mice (age, 6–8 weeks) were housed under constant temperature (22 °C ± 1 °C) with free access to food and water. LPS injection experiment was performed as previously described [35]. With detail, the mice were intraperitoneally injected with LPS at 10 mg/kg body weight. ACR was dissolved in a mixture of DMSO and corn oil and then were intraperitoneally injected at 0.1 mg/kg body weight or vehicle with the same volume 30 min before LPS treatment (*n* = 3 each group). All mice received an intraperitoneal injection of 100 mg/kg 5BAPA dissolved in PBS, at 30 min before being sacrificed.

### Immunofluorescence staining

The cells were seeded in Greiner 96-well microtiter plates (Greiner Bio-One), fixed in 4% paraformaldehyde for 10 min, and permeabilized in 0.5% Triton X-100 in PBS for 10 min at room temperature. The cells were then blocked in PBS containing 5% fetal bovine serum for 1 h at room temperature and incubated overnight at 4 °C with primary antibodies, including rabbit anti-cleaved caspase-3 (clCasp3, 1:500 dilution, 9661 S, Cell Signaling Technology, Danvers, MA, USA), mouse anti-Ki67 (1:200 dilution, 350502, BioLegend), rabbit anti-TG2 (1:200 dilution; RB-060-P0, Thermo Fisher Scientific), mouse anti-EpCAM (1:100 dilution, 324202, BioLegend), or mouse anti- HS (10E4 epitope, 1:200 dilution, 370255-S, AMSBIO, San Diego, CA, USA). The cells were washed and stained with Alexa Fluor 488/555-conjugated secondary antibodies (1:500 dilution, Thermo Fisher Scientific) for 20 min at room temperature. Fresh liver tissues were washed with PBS and then embedded in optimum cutting temperature (O.C.T.) compound (Sakura Finetek, Torrance, CA, USA) and snapfrozen in liquid nitrogen. Frozen liver sections (10 μm) were cut and mounted on slides and dried using a dryer prior to use. Cryosections were washed with PBS containing 0.1% Tween 20 (PBST), fixed with 4% paraformaldehyde (PFA) for 10 min at room temperature and washed twice with PBST for 5 min. Then, the sections were permeabilized with PBS containing 0.3% Triton X-100 for 10 min at room temperature and incubated in blocking buffer (5% fetal bovine serum in PBS containing 0.3% Triton X-100) for 30 min in a humidified chamber at room temperature. The sections were stained with TRITC-conjugated streptavidin (1:500, Jackson ImmunoResearch Laboratories, West Grove, PA, USA) diluted in same blocking buffer for 45 min at room temperature. Cell nuclei were visualized using DAPI (1:2000 dilution, Dojindo Molecular Technologies). Immunofluorescence staining signals were detected using an LSM 700 or ApoTome laser scanning confocal microscope (Carl Zeiss, Inc., Jena, Germany) or the ImageXpressMICRO High-Content Screening System (Molecular Devices). Morphological analysis was performed using the Multi-Wavelength Cell Scoring Application Module of the MetaXpress Image Analysis software (Molecular Devices).

### RNA isolation and real-time PCR

Total RNA samples were extracted using a FastGene RNA Basic Kit (NIPPON Genetics, Tokyo, Japan) and the amount and purity were evaluated using a DeNovix spectrophotometer (DeNovix, Wilmington, DE, USA). cDNA was synthesized using a PrimeScript RT Master Mix Kit (Takara Bio, Otsu, Japan). The primer sequences used in this study was summarized in Table S2. PCR reactions were performed using the SYBR Premix ExTaq II (Takara Bio) on a CFX96 Real-Time PCR Detection System (Bio-Rad Laboratories) or StepOne Real-Time PCR Systems (Applied Biosystems, Carlsbad, CA, USA). Gene expression was normalized to *GAPDH* or *ACTB* expression using the ΔΔCT method.

### nLC-MS/MS-based proteomic analysis

Proteome analysis was performed as previously described [63]. Briefly, JHH7 cells were lysed using a radioimmunoprecipitation assay buffer, purified, and digested using the filter-aided sample preparation method. nLC-MS/MS analysis was performed using an EASY-nLC 1000 (Thermo Fisher Scientific) and Q Exactive mass spectrometer (Thermo Fisher Scientific) equipped with a NANO HPLC capillary column C18 (0.075 mm ID × 150 mm length, 3 µm particle size, Nikkyo Technos, Tokyo, Japan) using a linear gradient (120 min, acetonitrile/0.1% formic acid) at a flow rate of 300 nL/min. The resulting MS and MS/MS data were searched against the Swiss-Prot database using Proteome Discoverer (ver.2.2, Thermo Fisher Scientific) with MASCOT search engine software (ver. 2.7.0, Matrix Science, London, UK).

### MALDI-TOFMS-based lipidomic analysis

Lipidomic analysis was performed as previously described [63]. In details, lipids were isolated from HCC cells JHH7 transduced with shCtl or shTG2 using the chloro-form/methanol/water extraction method. The chloroform phase containing lipids was evaporated under vacuum in SpeedVac (CC-105, TOMY, Tokyo, Japan) and reconstituted in 50 μL isopropanol (Wako Industries). Lipids were mixed with 15 mg/mL DHB matrix (1:5 v/v) in 90% acetonitrile/0.1% tri-fluoroacetic acid aqueous solution, and 0.5 μL of the mixture was loaded onto a MAL-DI-TOF target plate (MTP 384 target plate ground steel, Bruker Daltonics). Mass spectrometric analysis was performed using a rapifleX MALDI Tissuetyper mass spectrometer (Bruker Daltonics) at a spatial resolution of 20 µm. Peak calibration was carried out with a mixture of 10 mg/mL DCTB and 1 mg/mL cesium triiodide (1:1 v/v). Each collected spectrum was the sum of 10 single spectra obtained by shooting the laser at random positions on the target spot. Data were analyzed using FlexImaging software (Bruker Daltonics). Peak intensity was normalized to the protein concentration.

### Data mining

RNA-seq data were obtained from the liver hepatocellular carcinoma TCGA PanCancer RNA-seq database (www.cbioportal.org/) for 366 HCC tumor samples [64], from the CCLE database (https://portals.broadinstitute.org/ccle) for 25 HCC cell lines [65], and from CAGE analysis of 50 HCC liver tissues in a European cohort [66].

### Knowledge-based pathway analysis

To explore the upstream regulatory of EXT1 and the biological interpretation of TG2-correlated genes in HCC cells, gene type and molecular and cellular functions annotation and upstream regulator analysis was performed using knowledge-based functional analysis software Ingenuity Pathways Analysis (IPA; Ingenuity Systems, Mountain View, CA, USA) as previously described [67]. Upstream regulator analysis is one of the causal analytics algorithms in IPA that was developed to identify the upstream molecules in the data set that can explain the observed expression changes [68].

### Statistical analysis

Quantitative data were expressed as the mean ± SD of at least three biological replicates. The statistical significance of differences was assessed using the Student’s *t*-test and was set at *P* < 0.05. Unsupervised principal component analysis (PCA) was performed on the R platform. Volcano plots were visualized using the VolcaNoseR web tool (https://huygens.science.uva.nl/VolcaNoseR/) [69].

## Results

### ACR binds to and induces oligomer formation in TG2

**Fig. 1:**
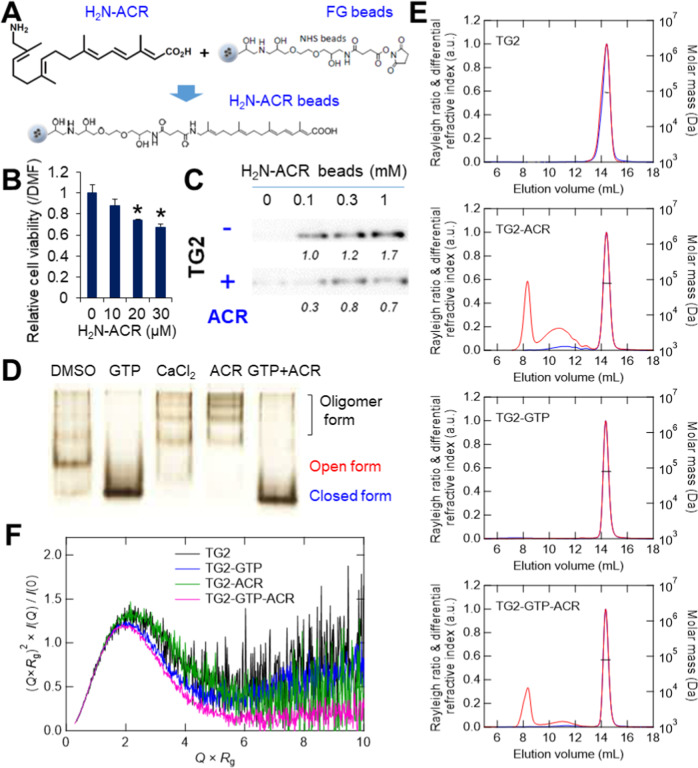
ACR induces conformational change of TG2. **A** Fixation of H_2_N-ACR onto magnetic FG beads. The amino-group of H_2_N-ACR was attached covalently by displacement of the NHS functional groups of the FG beads. **B** Cytotoxic activity of H_2_N-ACR on JHH7 cells. The cells were treated with increasing concentrations of H_2_N-ACR for 24 h. DMF was used as the vehicle control. The data are presented as the mean ± SD; *P < 0.05, Student’s *t* test. **C** Pull down assay of human recombinant TG2 incubated with increasing concentrations of H_2_N-ACR-immobilized beads in the presence (+) or absence (–) of 50 nmol ACR for competitive inhibition. TG2 was detected by western blot analysis. Intensity value of each blot was shown relative to the treatment with 0.1 mM H_2_N-ACR-immobilized beads in the absence of ACR. **D** Native PAGE conformation study of recombinant human TG2. TG2 was incubated with DMSO, 500 μM GTP, 5 mM CaCl_2_, 100 μM ACR, or 100 μM ACR in the presence of 500 μM GTP for 2 h at room temperature and then subjected to electrophoresis. The protein bands were visualized with silver staining. **E** SEC-MALS chromatograms. Rayleigh ratio, differential refractive index and molar mass are displayed by red, blue, and black lines, respectively. **F** Dimensionless Kratky plots measured by SEC-SAXS of recombinant human TG2 (5.6 mg/mL) incubated with vehicle control EtOH, 100 μM GTP, 100 μM ACR, or 100 μM ACR in the presence of 100 μM GTP for 2 h at room temperature.

Since growth suppression was not observed in a previous study of HCC cells with the ethyl analogs of ACR at the carboxyl group [22], we synthesized a novel derivative of ACR by introducing an amino group on the terminus opposite of the carboxyl group (H_2_N-ACR) (Fig. S1) and established an analytical strategy to examine the binding of TG2 and ACR with H_2_N-ACR immobilized magnetic nanobeads (FG beads) (Fig. 1A). A dose-dependent suppressive effect of H_2_N-ACR on the proliferation of JHH7 cells was observed (Fig. 1B). The pull-down of recombinant human TG2 incubated with H_2_N-ACR immobilized FG beads showed a dose-dependent binding of TG2 and H_2_N-ACR, which was inhibited by the addition of free ACR (Fig. 1C, original western blot data 1). This suggests that ACR binds directly to TG2. Next, native PAGE analysis was performed to assess the direct effect of ACR binding on the conformation structure of TG2. Similar to the enzyme activator CaCl_2_, ACR induced the oligomer formation of TG2, which was almost completely prevented by the addition of GTP (Figs. 1D and S2A). Similar effects were not observed with the ethyl analogs of ACR (Fig. S2B, C). Intriguingly, the vitamin K2 analog, SVK30, with an ACR-like structure also induced oligomer formation of TG2 (Fig. S2D).

To evaluate the structural changes induced by ACR, the absolute molar mass of TG2 was determined by SEC-MALS. First, recombinant human TG2 was synthesized and purified in-house to obtain sufficient quantities for analysis. A dose-dependent increase in transamidase activity was confirmed based on 5BAPA incorporation onto a casein-coated plate (Fig. S3A). The results of the SEC-MALS analysis measured for TG2 apo, TG2-GTP complex, TG2-ACR complex, and TG2-GTP-ACR complex are displayed in Fig. 1E. The molar masses obtained from the analysis of the main peaks were 91,650 for TG2 apo, 79,530 for TG2-GTP complex, 79,210 for TG2-ACR complex, and 79,040 for TG2-GTP-ACR complex, respectively. The molecular weight observed for TG2 apo was slightly larger than expected, but these values essentially indicate that TG2 is monomeric in all conditions. A broad peak at the elution volume of 10–12 mL and a sharp peak around 8 mL, upstream of the main peak, were observed in the two conditions where ACR bound. The peak observed around 8 mL is considered to be derived from aggregates, while the peak observed at the region of 10–12 mL suggests the oligomer formation of TG2. Therefore, in line with the native PAGE analysis, the binding of ACR was shown to have induced the oligomer formation of TG2. On the other hand, in the presence of GTP, the height of the peak observed in the 10–12 mL region decreased, suggesting that the binding of GTP suppressed the oligomer formation.

SEC-SAXS analysis was performed under four different structure conditions to clarify the structural state of TG2 in solution as well as SEC-MALS. Fig. S3B showed the Guinier plots of the scattering intensities obtained under each condition. The *R*_*g*_ value, a measure of molecular size obtained from the Guinier analysis, is 35.0 ± 0.8 Å for TG2 apo, 32.2 ± 0.2 Å for the TG2-GTP complex, 35.5 ± 1.0 Å for the TG2-ACR complex, and 31.8 ± 0.2 Å for the TG2-GTP-ACR complex, respectively. The values for the two conditions bound with GTP were around 3 Å smaller than those of other conditions. Dimensionless Kratky plots were displayed in Fig. 1F to qualitatively compare the four structure states in solution by using the *R*_g_ and *I*_(0)_ values obtained from the Guinier analysis. Although a bell-shaped peak is observed in all conditions, indicating the presence of a folded structure region, the position and width of the peak were different from each other. The TG2 apo and TG2-ACR complexes had a peak at ~2.2 of *Q*×*R*g, which was a little wider than the other two conditions. The position of the peak for TG2-GTP-ACR complex was almost the same as that of TG2-GTP complex (~2.0 of *Q*×*R*g); however, the width of the peak was even narrower than that of TG2-GTP complex. Both the shift of the peak to the lower *Q* region and the decrease in the peak width indicated a change to a more compact structure state. Although the comparison in the *Q* region higher than the peak was not clear due to the signal-to-noise ratio of the data, the TG2-GTP-ACR complex plot approached the baseline asymptotically, suggesting that it was in the most compact structural state among the four conditions. The further investigation of the structural state of TG2 in solution was carried out using the previously reported crystal structures. Two PDB structures for TG2 have been published by X-ray crystallography [31]. One is 2Q3Z (open conformation) bound to the inhibitor, and the other is 1KV3 (closed conformation) bound to GDP (Fig. S3C). In Fig. S3D, the experimental SAXS curves of the four structure conditions were superimposed with the theoretical SAXS curves calculated from these two PDB structures. The *R*_g_ values obtained from each theoretical scattering curve were 40.3 Å for 2Q3Z and 30.0 Å for 1KV3, respectively. Although the χ2 value indicates whether each structure state was closer to the open or closed conformations, it was clear that the four structure states of TG2 were not identical to either conformation as far as the residual plots were concerned (Fig. S3D). Since SAXS provides information on the average structure in solution, this result suggests that TG2 in solution is either in a mixed state of two structural forms or in an average structural state different from these two. Hence, the volume fractions of the two structure forms in solution under the four conditions were calculated using these two PDB structures as probes. The theoretical SAXS curves synthesized based on the obtained volume fractions were superimposed on each experimental curve in Fig. S3E. The plots of residuals converged to almost zero in all results, indicating that the agreement was better than that obtained when comparing with open and closed TG2 respectively in Fig. S3D. The *χ*^2^ values were also smaller than those in the directly comparison with open and closed TG2, and the volume fractions showed that not only TG2 apo and TG2-ACR complex had more open form fractions but also that the GTP-bound to TG2 increased the fraction of closed form (Fig. S3E). This result was consistent with the trend of *R*g values and dimensionless Kratky plots.

### Transamidase activity of cytoplasmic TG2 mediates HCC cell proliferation

**Fig. 2:**
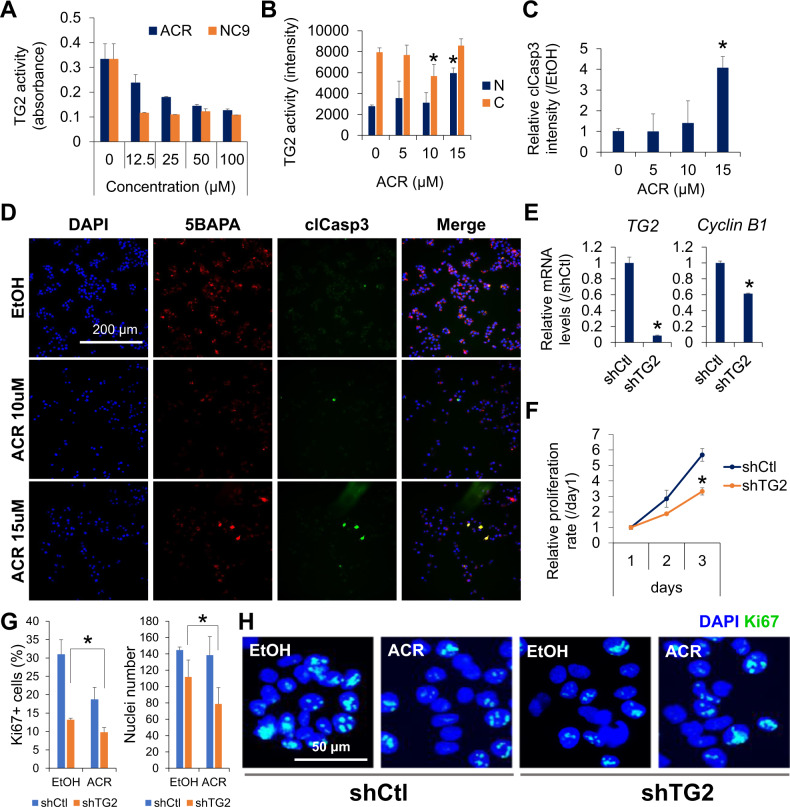
Dose-dependent roles of ACR on intracellular TG2 activity. **A** Dose-dependent inhibitory effects of ACR and NC9 on transamidase activity. 5BAPA incorporation onto casein-coated plate in the presence of TG2, 5 mM CaCl_2_ and increasing concentrations of ACR or NC9 for 1 h at 37 °C was examined as the indicator of TG2 transamidase activity. **B** Dose-dependent effects of ACR on intracellular transamidase activity of nuclear (N) and cytoplasm (C) TG2 in JHH7 cells treated with increasing concentrations of ACR as indicated for 4 h at 37 °C. Fluorescence intensity resulting from the TRITC-based incorporation of 5BAPA into the cells was examined as an indicator of intracellular TG2 transamidase activity. **C** Relative intensity of clCasp3 and **D** representative immunofluorescence staining for 5BAPA and clCasp3 of JHH7 cells treated with increasing concentrations of ACR for 4 h. Scale bar, 200 μm. **E** Relative gene expression of TG2 (*TGM2*) and cyclin B1 (*CCNB1*) and **F** cell proliferation of JHH7 cells transduced with shCtl or shTG2 shRNA lentiviral particles. **G** Percentages of Ki67 positive (Ki67+) cells (left) and nuclei number (right) and **H** representative immunofluorescence staining of Ki67 in shCtl and shTG2-transduced JHH7 cells treated with EtOH or 5 μM ACR for 24 h. Scale bar, 50 μm. The data are presented as the mean ± SD; *P < 0.05, Student’s *t* test.

Then, we assessed the potential effect of ACR on TG2 activation. Similar to the irreversible TG2 inhibitor NC9, ACR significantly inhibited transamidase activity in the presence of CaCl_2_ in a dose-dependent manner (Fig. 2A). We hypothesized that the binding of CaCl_2_ stabilized the open form and induced transamidase activity of TG2, in turn inducing the covalent cross-linking of TG2 itself. In contrast, the binding of ACR induced the oligomerization of TG2 as a linker and inhibited transamidase activity. Indeed, oligomer formation of CaCl_2_-incubated—not ACR-treated—TG2 was observed after boiling, based on SDS-PAGE analysis (Fig. S4A). Next, we examined the dose-dependent effects of ACR on the intracellular transamidase activity of TG2 and apoptosis in JHH7 cells (Fig. 2B–D). In agreement with previous reports [22, 36], a dose-dependent effect of ACR on the cell viability of JHH7 cells was observed, with a half-maximal inhibitory concentration (IC_50_) at 16.7 μM (Fig. S4B). To avoid unspecific effects due to cytotoxicity, we chose 15 μM, which is close to the IC_50_ of ACR, as the high dose of ACR and 5 and 10 μM of ACR as the low doses in this study. Additionally, we used 5BAPA as a cell permeable amine probe to examine the intracellular transamidase activity of TG2 [34, 70]. 5BAPA is a biotinylated substrate for TG2, which is able to participate in the acyltransferase reaction and covalently attached to intracellular protein substrates at their glutamine residues [71, 72]. Immunofluorescence triple staining for DAPI nuclear counterstaining, TG2 transamidation as indicated by 5BAPA incorporation, and apoptotic cells labeled with the apoptosis marker clCasp3 all demonstrated that low dose ACR without apoptosis induction significantly suppressed the transamidase activity of cytoplasmic TG2 (Fig. 2B–D). In contrast, ACR at a high dose induced the transamidase activity of nuclear TG2 along with apoptosis in JHH7 cells, especially in clCasp3 positive apoptotic cells (Fig. 2B–D). To elucidate the molecular mechanism of ACR-induced nuclear TG2 activation, we examined the effect of ACR on intracellular Ca^2+^ concentrations. Similar to its effect on the intracellular transamidase activity, ACR, at high doses exclusively, increased intracellular Ca2^+^ concentrations in JHH7 cells (Fig. S4C). A similar effect of ACR on the intracellular transamidase activity of TG2 and apoptosis was also observed in HCC cell line Huh7 (Fig. S5). Next, we characterized the functional role of TG2 in cell proliferation and survival. Loss-of-function analysis revealed that knockdown of TG2 using shRNA inhibited the gene expression of the cell cycle regulator *cyclin B1* (Fig. 2E) and the proliferation of JHH7 cells (Fig. 2F). The percentage of proliferating cells labeled with the cellular proliferation marker Ki67 and nuclei number was significantly decreased in shTG2-transduced JHH7 cells compared to that in shCtl-transduced cells and the combination of ACR and TG2 knockdown further suppressed the proliferation of JHH7 cells (Fig. 2G, H).

### TG2 regulates the stemness of HCC cells

**Fig. 3:**
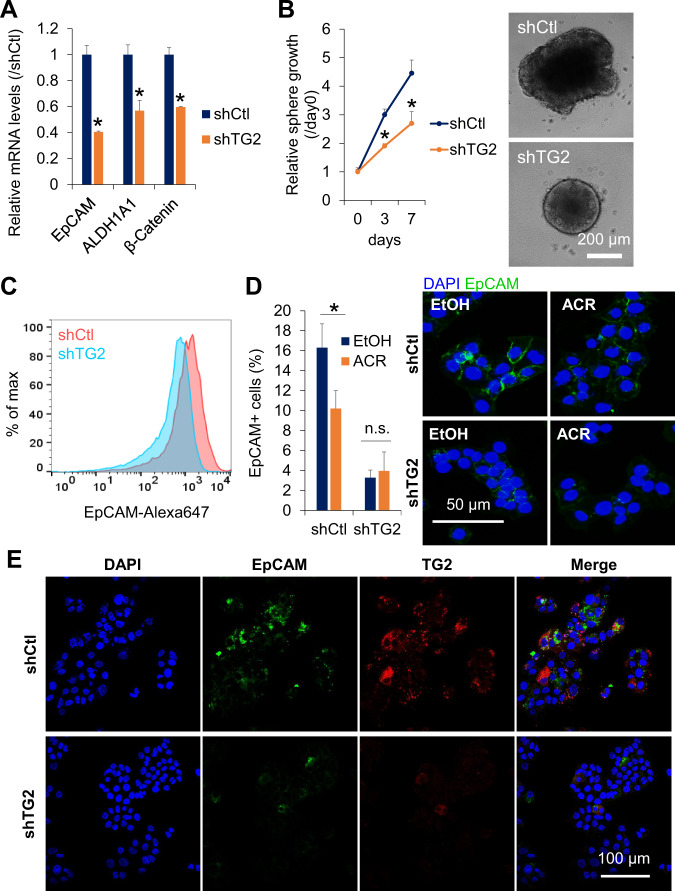
TG2 regulates proliferation of liver CSCs in monolayer and spheroid cultures. **A** Relative gene expression of stemness-related genes *EpCAM*, *ALDH1A1*, and *CTNNB1* and **B** spheroid proliferation in shCtl and shTG2-transduced JHH7 cells. **C** Flow cytometric analysis of EpCAM protein expression in shCtl and shTG2-transduced JHH7 cells. **D** Immunofluorescence triple staining of DAPI (blue), EpCAM (green), and TG2 (red) in shCtl and shTG2-transduced JHH7 cells. Scale bar, 100 μm. **E** Percentages (left) and representative immunofluorescence staining (right) of EpCAM+ cells in shCtl and shTG2-transduced JHH7 cells treated with EtOH or 10 μM ACR for 24 h. Scale bar, 50 μm.

Next, we examined the functional role of TG2 in regulating the stemness of HCC cells. First, a significant decrease in the expression of stemness-related genes, including *EpCAM*, *ALDH1A1*, and *CTNNB1* was observed in shTG2-transduced JHH7 cells compared to shCtl-transduced cells (Fig. 3A). In addition, knockdown of TG2 with shRNA significantly suppressed the proliferation of JHH7 cells grown in spheroid cultures (Fig. 3B). Consistent with the gene expression data, flow cytometric analysis showed a decrease in the presence of EpCAM+ cells in TG2 knockdown JHH7 cells (Fig. 3C). Similarly, immunofluorescence staining showed that the number of EpCAM+ cells was dramatically reduced in shTG2-transduced JHH7 cells compared to shCtl-transduced cells (Fig. 3D). Furthermore, immunofluorescence double staining identified a TG2 and EpCAM co-expression subpopulation in heterogeneous JHH7 cells, which were diminished by TG2 knockdown (Fig. 3E).

### Sensitivity of CSC-like HCC cells in response to TG2 inhibition

**Fig. 4:**
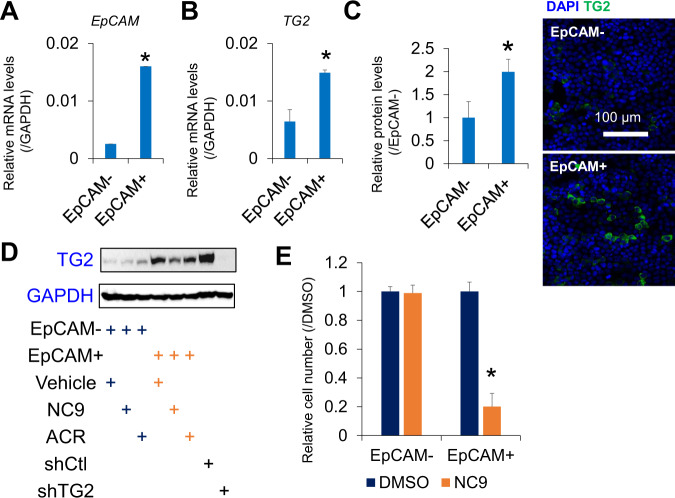
TG2 high expression liver CSCs are selectively targeted by NC9. Relative gene expression of **A** *EpCAM* and **B** *TGM2* in sorted EpCAM+ and EpCAM- JHH7 cells. **C** Relative fluorescence intensity (left) and representative immunofluorescence staining (right) of TG2 in EpCAM+ and EpCAM– JHH7 cells. **D** Protein expression of TG2 in sorted EpCAM+ and EpCAM- JHH7 cells treated with vehicle control, 25 μM NC9 or 10 μM ACR for 16 h or shCtl and shTG2-transduced JHH7 cells. **E** Cell viability of EpCAM+ and EpCAM- JHH7 cells treated with 25 μM NC9 for 48 h. The data are presented as the mean ± SD; *P < 0.05, Student’s *t* test.

Next, EpCAM+ and EpCAM– subpopulations were isolated from JHH7 cells using fluorescence-activated cell sorting (Fig. 4A). Both the gene (Fig. 4B) and protein (Fig. 3C) of TG2 were expressed at significantly higher levels in EpCAM+ liver CSC-like cells than in EpCAM– cells. Intriguingly, ACR and the irreversible TG2 inhibitor, NC9, reduced TG2 protein expression levels in EpCAM+ cells (Fig. 4D, original western blot data 2). NC9 selectively suppressed the proliferation of EpCAM+ cells (Fig. 4E), suggesting differential sensitivity to TG2 inhibition in EpCAM+ and EpCAM− cells. Additionally, NC9 significantly suppressed MYCN gene expression in JHH7 cells (Fig. S6A), which was recently identified as a marker of liver CSCs and was selectively inhibited by ACR [22, 73]. Next, protein expression levels of TG2 were examined in the livers of DEN-induced hepatic tumorigenesis mice [74]. Similar to the liver progenitor marker pan-cytokeratin, TG2 was highly expressed in DEN-induced liver tumors and strongly inhibited in the livers of mice administered with ACR (Fig. S6B, original western blot data 3). Notably, in the DEN and high-fat diet-induced hepatocarcinogenesis mice, we previously reported that ACR inhibited hepatic lipid metabolism and inflammation along with the suppression of liver tumorigenesis and proposed that targeting inflammatory microenvironment was important for the prevention of HCC by ACR [16, 74]. As a proof-of-concept to show whether ACR might affect the in vivo transamidase activity of TG2 in inflammatory liver, the effect of ACR on TG2 activity was examined in the liver of LPS-challenged mice [35]. ACR could strongly suppress in vivo transamidase activity of TG2 as indicated by the incorporation of the TG2 probe 5BAPA in the liver of LPS-challenged mice (Fig. S6C).

### TG2 regulates EXT1-mediated HS biosynthesis in HCC cells

**Fig. 5:**
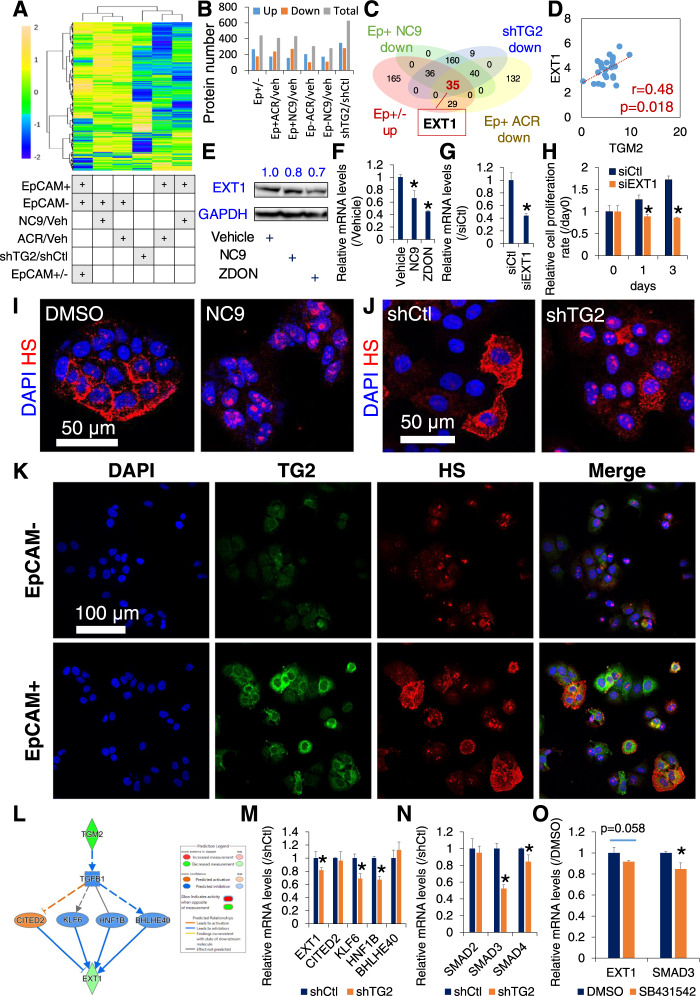
TG2 mediates HS signaling in liver CSCs. **A** Hierarchical clustering of fold change expression for the proteins measured by nLC-MS/MS in sorted EpCAM+ and EpCAM– JHH7 cells treated with vehicle control, 25 μM NC9, or 10 μM ACR for 16 h or shCtl and shTG2-transduced JHH7 cells. **B** Summary of the number of differentially expressed proteins with a threshold of more than 2-fold. **C** Comparison of downregulated proteins by NC9 and ACR in EpCAM+ JHH7 cells, downregulated proteins between shTG2 and shCtl JHH7 cells, and upregulated proteins between EpCAM+ and EpCAM– JHH7 cells. The three common proteins are highlighted. **D** Correlation between gene expression of *TGM2* and *EXT1* in a total of 25 HCC cell lines in the CCLE database. The data are presented as a robust multiarray average. **E** Protein expression of EXT1 in JHH7 cells treated with vehicle control, 25 μM NC9, or 25 μM ZDON for 16 h. **F** Gene expression of *EXT1* in JHH7 cells treated with vehicle control, 25 μM NC9, or 25 μM ZDON for 4 h. **G** Gene expression of *EXT1* and **H** cell proliferation of JHH7 cells transfected either with siCtl or siEXT1 for 24 h. Representative immunofluorescence staining for HS (**I**) in JHH7 cells treated with DMSO or 25 μM NC9 for 24 h and **J** in shCtl and shTG2-transduced JHH7 cells. Scale bar, 50 μm. **K** Immunofluorescence triple staining of DAPI (blue), TG2 (green), and HS (red) in EpCAM+ and EpCAM– JHH7 cells. Scale bar, 100 μm. **L** A schematic model of TGF-β1 activation-dependent regulatory network underlying the control of EXT1 gene expression by TG2 in HCC cells generated using “Upstream Regulator Analysis” and “Path Explore” functions in IPA platform. Gene expression of (**M**) *EXT1* and 4 candidate upstream transcription regulators of EXT1 that *CITED2*, *KLF6*, *HNF1B*, and *BHLHE40* and (**N**) downstream targets of TGF-β1 that *SMAD2*, *SMAD3* and *SMAD4* in shCtl and shTG2-transduced JHH7 cells. (**O**) Gene expression of *EXT1* and *SMAD3* in JHH7 cells treated with DMSO or a TGF-β small molecule inhibitor SB431542 at 10 μM for 4 h. The data are presented as the mean ± SD; *P < 0.05, Student’s *t*-test.

To elucidate the mechanism underlying the regulation of cell proliferation by TG2, nLC-MS/MS-based proteome analysis was performed to identify the molecular targets of TG2 in sorted EpCAM+ and EpCAM– JHH7 cells treated with a vehicle control, ACR, or NC9, and in JHH7 cells transduced with shCtl or shTG2 (Table S3). Hierarchical clustering was performed on fold change expression data of EpCAM+ cells treated with ACR and NC9, as well as on heterogeneous JHH7 cells with shTG2 knockdown, demonstrating similar effects on protein expression profiles. Conversely, these effects showed opposite trends compared to those in protein expression profiles induced by ACR and NC9 treatment in EpCAM- cells, as well as differences between EpCAM+ and EpCAM- cells. (Fig. 5A). The number of differentially expressed proteins (with a cutoff of more than 2-fold) induced by ACR or NC9 was higher in EpCAM+ cells than that in EpCAM– cells, suggesting that EpCAM+ cells are more sensitive to TG2 inhibition (Fig. 5B). Collectively, these results suggest that genetic or pharmacological inhibition of TG2 selectively targets liver CSC-enriched signaling pathways. Next, the molecular targets of TG2 were identified by comparing proteins upregulated in EpCAM+ cells than those in EpCAM– cells, proteins downregulated by ACR or NC9 in EpCAM+ cells, and protein downregulated by TG2 knockdown with shTG2 in JHH7 cells (Fig. 5C), as well as by comparing proteins downregulated in EpCAM+ cells than that in EpCAM– cells, proteins upregulated by ACR or NC9 in EpCAM+ cells and proteins upregulated by TG2 knockdown with shTG2 in JHH7 cells (Fig. S7A). Thirty-five commonly downregulated proteins, including EXT1, were identified as the molecular target of TG2 in HCC (Figs. 5C and S7B), while 5 commonly upregulated proteins were identified (Fig. S7A). As a negative control of the unintegrated genes, the expression of galectin-1 (LGALS1) was significantly inhibited by TG2 knockdown but not by NC9/ACR (Fig. S7C). Using data from three previous studies, we assessed correlations in gene expression between TG2 (*TGM2*) and *EXT1*; EXT1 was strongly correlated with that of TG2 in human HCC (Figs. 5D and S7D–F) and was selected for further analysis. Both the irreversible TG2 inhibitors NC9 and ZDON inhibited the protein expression (Fig. 5E, original western blot data 4) and gene expression (Fig. 5F) of EXT1 in JHH7 cells. As functional evidence, transfecting JHH7 cells with a pool of three target-specific siRNAs against *EXT1* (Fig. 5G) significantly inhibited cell proliferation (Fig. 5H). Given the critical role of EXT1 as a glycosyltransferase in HS biosynthesis especially HS chain elongation [75], we investigated whether TG2 can regulate HS biosynthesis in HCC cells. Immunofluorescence staining showed that TG2 inhibition using either NC9 **(**Fig. 5I) or shTG2 (Fig. 5J) dramatically suppressed the level of HS in JHH7 cells. To confirm the effect of TG2 inhibition and EXT1-HS signaling, we included another TG2 inhibitor CTM, which is widely used as a competitive inhibitor for the TG-catalyzed transamidation reaction [32, 76]. Significant inhibitory effects of CTM on EXT1 and HS expression were observed in both JHH7 and Huh7 cells (Fig. S8). Notably, immunofluorescence double staining demonstrated that the expression of HS was higher and showed co-expression with TG2 in EpCAM+ cells compared to EpCAM– cells (Fig. 5K). Consistent with this result, we observed a significant increase in EXT1 gene expression in EpCAM+ cells compared to EpCAM- cells (Fig. S7G). Additionally, we found that ACR and NC9 treatment had a combined inhibitory effect on EXT1 gene expression in EpCAM+ cells (Fig. S7G).

To further explore the molecular mechanism by which TG2 regulates EXT1 gene expression, upstream regulator analysis was performed using IPA platform. Based on the proteome profiling data, CITED2, KLF6, HNF1B, and BHLHE40, whose activity was induced or inhibited following TG2 knockdown in JHH7 cells, were predicted as the upstream transcription regulators of EXT1 (Fig. 5L). We then examined the gene expression of these transcription regulators using PCR analysis and confirmed that the gene expression of KLF6 (krueppel-like factor 6) and HNF1B (hepatocyte nuclear factor 1 homeobox B) was significantly downregulated following TG2 knockdown in JHH7 cells (Fig. 5M). Previous studies showed direct evidence that loss-of-function of KLF6 or HNF1B decreased expression of EXT1 in cultured mouse bone marrow-derived macrophages [77] or ovarian cancer cell line SKOV3 [78]. Then, we examined the relationship between TG2 and KLF6 and HNF1B using the “Path Explore” function of IPA platform. No direct relationship was observed between TG2 and KLF6 and HNF1B. Interestingly, transforming growth factor beta 1 (TGF-β1)-associated indirect relationships between TG2 and KLF6 and HNF1B were observed. The presence of TG2 on plasma membrane and its transamidase activity are required for the activation of TGF-β1 [79, 80]. The activity of TGF-β1 was predicted to be inhibited following TG2 knockdown in JHH7 cells (Fig. 5L). In line with this prediction, gene expression of downstream targets of TGF-β1, including SMAD3 and SMAD4, was significantly inhibited by TG2 knockdown in JHH7 cells (Fig. 5N). Finally, to provide direct evidence for the regulatory role of TGF-β1 on EXT1 gene expression, loss-of-function analysis of TGF-β1 was performed using a small molecule inhibitor SB431542. At the concentration that SB431542 significantly inhibited SMAD3 gene expression, a decrease in EXT1 gene expression but not significant (*P* = 0.058) was observed in JHH7 cells (Fig. 5O).

### Correlation of TG2 and membrane signaling in human HCC tumors

**Fig. 6:**
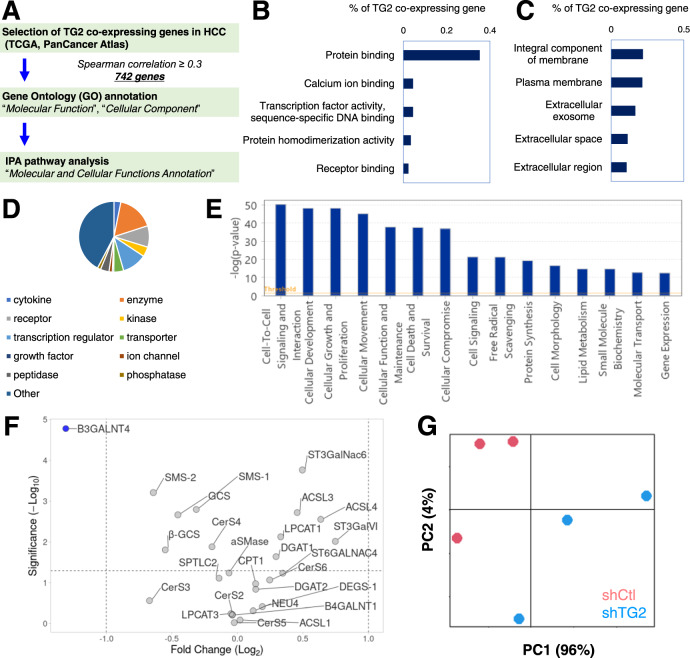
Correlation of TG2 and membrane signaling in human HCC tumors. **A** Schematic overview to identify TG2-correlated genes in human HCC tumors through an analysis of the Cancer Genome Atlas (TCGA). Gene Ontology (GO) annotation of **B** “Molecular Function” and **C** “Cellular Component” with TG2-correlated genes in human HCC tumors. **D** Gene type and **E** molecular and cellular functions annotation analysis with TG2-correlated genes in human HCC tumors in IPA platform. **F** Volcano plot of the gene expression profile of 26 well-known enzymes involved in cell recognition and membrane protein regulation and **G** PCA plot of lipidomic profiling analysis with MALDI-TOFMS in shCtl and shTG2-transduced JHH7 cells.

To further explore the clinical relevance of the correlation of TG2 and membrane signaling in liver cancer, a data mining was performed with the Cancer Genome Atlas Liver Hepatocellular Carcinoma (TCGA-LIHC) data, which contains RNA-sequencing data from 366 HCC tumor samples (Fig. 6A). With a threshold of Spearman’s correlation coefficient >0.3, 742 TG2-correlated genes were identified in human HCC tumors. Of interest, in agreement with our findings regarding the role of TG2 in regulating membrane-related signaling in HCC cells, Gene Ontology (GO) annotation for “Molecular Function” and “Cellular Component” indicated that most of the TG2-correlated genes in human HCC tumors encode proteins that are in membrane and involved in protein binding function (Fig. 6B, C). Further gene annotation and molecular and cellular functions annotation analysis with IPA platform showed that the mostly enriched gene types of TG2-correlated genes were “enzyme”, “transcription factors” and “receptors” and the most enriched molecular and molecular and cellular functions were “Cell-To-Cell Signaling and Interaction”, “Cellular Development”, and “Cellular Growth and Proliferation” in human HCC tumors (Fig. 6D, E and Table S4). As functional evidence, the role of TG2 knockdown on the gene expression of 26 well-known enzymes involved in cell recognition and membrane protein regulation were examined and robust changes in gene expression, most of which were significantly inhibited by TG2 knockdown, were observed in HCC cells. Especially, the gene expression of beta-1,3-galactosyltransferase 4 (*B3GALT4*), a glycosyltransferase involved in ganglioside synthesis, was dramatically inhibited by TG2 knockdown in HCC cells (Fig. 6F). In line with these findings, a high-throughput lipidomic profiling analysis with MALDI-TOFMS showed a strong effect of TG2 knockdown on membrane lipid profiles in HCC cells (Fig. 6G and Table S5). Collectively, our study highlighted the roles of TG2 inhibitors as a potential therapeutic strategy in the prevention of HCC by targeting liver CSCs, which is important in advancing our knowledge about TG2 in liver CSCs.

## Discussion

**Fig. 7:**
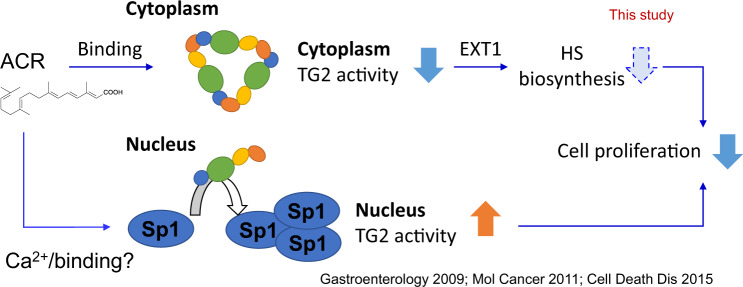
Schematic diagram of multiple roles of ACR on intracellular TG2 activity in regulating cell proliferation of liver CSCs. ACR directly bonded to and induced oligomer formation of TG2, which inhibited the transamidase activity of cytoplasmic TG2 in HCC cells. Loss-of-function of TG2 suppressed the expression of stemness-related genes, spheroid proliferation and selectively induced cell death of the EpCAM+ liver CSC subpopulation in HCC cells. Mechanistically, TG2 inhibition suppressed the gene and protein expression of the HS biosynthesis enzyme, EXT1, and HS biosynthesis in HCC cells. TG2 and HS were highly expressed and co-located in EpCAM+ liver CSCs, highlighting a potential role of TG2-mediated HSPG signaling in regulating cell proliferation of liver CSCs. In contrast, ACR at a high dose increased intracellular Ca2^+^ concentration along with clCasp3^+^ cells, which probably contributed to the enhanced transamidase activity of nuclear TG2, cross-linking of nuclear transcription factors such as Sp1, and apoptosis in HCC cells as previously reported [32, 36, 37].

In this study, we described the multiple roles of ACR on intracellular TG2 activity in regulating cell proliferation of liver CSCs and identified a critical role of TG2 in the regulation of EXT1-related signaling using proteome and lipidome analyses (Fig. 7). By combining a binding assay with high-performance magnetic nanobeads and structural dynamic analysis with native gel electrophoresis and SEC-MALS/SAXS analyses, we provide direct evidence at the molecular level that ACR directly bonded to and induced oligomer formation of TG2. In addition to the retinoic acid-binding proteins and nuclear receptors [11–13], our study reported for the first time that TG2 is a novel binding protein of ACR and highlighted a potential mechanism of the anti-HCC effect of ACR independent retinoic acid receptor pathways. Similar to their suppressive effects on HCC cell proliferation, induction of TG2 oligomer formation was observed with SVK30, having an ACR-like structure, but not with the ethyl analogs of ACR [22, 81], suggesting that the binding of ACR with TG2 is likely related to the carboxylate terminus of the chain containing three isoprene residues of ACR. These findings provide useful insights for designing derivatives to target TG2. On the other hand, GTP strongly prevented ACR-induced oligomer formation of TG2. It is unclear whether there is a direct interaction between GTP and ACR, or whether ACR specifically binds to TG2 in an open conformation, and not in a closed conformation favored by GTP binding. However, since the oligomer formation of TG2 induced by ACR disappeared after SDS-PAGE, it is unlikely that the covalent cross-linking of TG2 was due to enhanced transamidase activity, as observed with CaCl_2_ treatment [31]. In addition, ACR inhibited the Ca^2+^-dependent transamidase activity of purified TG2 in solution with a level of effect similar to that of the transamidase site-targeted TG2 inhibitor, NC9 [47]. We hypothesize that ACR induces polymerization of TG2 through protein-ligand interaction and the ACR-linked TG2 inhibited its catalytic activity. SAXS analysis showed that ACR slightly favored the open conformation of the monolayer TG2. Future studies should examine whether ACR targets the transamidase site and locks TG2 in an open conformation, in a similar manner to NC9 [82].

Imaging of intracellular transamidase activity in HCC cells demonstrated stage- and subcellular location-dependent effects of ACR on TG2 activity. It is widely accepted that TG2 is silent as a transamidase under physiological conditions, and TG2 activation under stress conditions, such as high levels of Ca^2+^ (>1 mM), leads to protein cross-linking and cell death [83]. In our previous studies, both in vitro and ex vivo imaging showed undetectable transamidase activity of TG2 in normal human and mouse hepatocytes [34, 35]. In this study, along with the increase in TG2 expression in HCC cells and DEN-induced liver tumors in mice, a strong transamidase activity of TG2 was observed in the cytosol of HCC cells, which was dramatically suppressed by ACR at low doses without apoptosis induction. Conversely, ACR at high doses increased intracellular Ca^2+^ concentration accompanied by an increase in clCasp3^+^ cells, which probably contributed to the enhanced transamidase activity of nuclear TG2, cross-linking of nuclear transcription factors such as Sp1, and apoptosis in HCC cells [32, 36, 37]. We previously reported that ACR strongly induced endoplasmic reticulum (ER) stress in HCC cells in a manner dependent on lipid desaturation [61]. Given that ER is the main storage organelle and source of intracellular Ca^2+^ [84], we hypothesized that ACR might indirectly induce nuclear activation of TG2 by regulating intracellular Ca2^+^ concentrations. Notably, ACR-induced nuclear activation of TG2 was also observed in clCasp3 negative cells, suggesting an upstream positive regulatory role of nuclear TG2 in apoptosis induction. Indeed, knockdown of TG2 blocked the cell death induced by ACR and SVK30 in HCC cells [36, 81]. Here, we also showed that knockdown of TG2 repressed cell cycle progression by reducing the number of Ki67+ proliferating cells, suggesting that TG2 is a target of ACR in HCC cells. Further studies should identify and compare TG2 substrates in the cytosol and nucleus of HCC cells to characterize the context-dependent roles of TG2 activation in the pathology of liver tumorigenesis as well as the molecular targets of ACR.

Herein we provided experimental evidence that loss-of-function of TG2 suppressed the expression of stemness-related genes, spheroid proliferation, and selectively induced cell death in the EpCAM+ liver CSC subpopulation in HCC cells. Similar to the changes in Ki67+ proliferating cells, the growth-inhibitory effect of ACR on EpCAM+ cells was significantly reduced in TG2 knockdown HCC cells, suggesting that TG2 is a target of ACR in liver CSCs. Further proteomic analysis revealed that TG2 inhibition suppressed the gene and protein expression of the HS biosynthesis enzyme, EXT1, and HS biosynthesis in HCC cells. HS is a glycosaminoglycan composed of linear sulfated polysaccharides. Heparan sulfate proteoglycans (HSPGs) are glycoproteins found on the cell surface and extracellular matrix (ECM) and contain at least one covalently bound HS chain. HSPGs, especially syndecan-4, are critical in the extracellular trafficking of TG2 onto the cell surface and into the ECM, while it does not affect the transamidase activity of TG2 itself [85]. The HS binding sites on TG2 are located in a narrow pocket, which is closely related to its closed conformation. Stabilization of TG2 in its open conformation almost completely disrupts TG2-HS binding [86, 87]. Our data suggest that TG2 plays an upstream regulatory role in HS biosynthesis, potentially through the regulation of EXT1 gene expression by TGF-β1 activation-dependent pathways. The critical role of the TG2-HSPG interaction in the activation of TGF-β [88] suggests that conformational changes in TG2 induced by ACR or TG2 inhibitors may disrupt the interplay between TG2 and HS chains of HSPGs, which might further affects TGF-β downstream signaling, such as HS biosynthesis.

HSPGs play important roles in embryonic development and oncogenesis by regulating membrane signaling complexes, such as the assembly of ternary complexes with growth factors or Wnt ligands and their receptors, in both HS chain-dependent and -independent manners [89, 90]. Therefore, there is an emerging interest in HSPGs as potential therapeutic targets for HCC. For example, an HSPG glypican-3 (GPC3) is highly expressed in HCC [91], and an antibody targeting the HS chain of GPC3 was shown to block Wnt signaling and suppress tumor growth in a mouse model of HCC [92]. EXT1 catalyzes the elongation of the HS chain [75] and its inhibition selectively reduced the CSC subpopulation, concomitant with decreased EMT and drug resistance in breast cancer cells [93]. In contrast, the tumor-suppressor function of EXT1 has been reported in sporadic human malignancies [94]. Here, we showed that EXT1 knockdown inhibited the proliferation of HCC cells. In addition, TG2 and HS were highly expressed and co-located in EpCAM+ cells, highlighting the potential role of TG2-mediated EXT1 signaling in liver CSCs. Given the heterogeneous saccharide length and sulfation of the HS chain, HSPGs have opposing roles in cancer biology that are determined by the dynamic tumor microenvironment [90]. Future experiments to identify the specific HSPGs involved in TG2-mediated EXT1 signaling are required to advance our understanding of CSC biology and promote HS-targeted drug development against HCC.

In summary, we (1) provided a structural basis for the subcellular location-dependent effects of ACR on TG2 activity; (2) characterized the tumor-promoting roles of cytoplasmic TG2 in HCC and the therapeutic potential of TG2 inhibition in the selective depletion of liver CSCs; and (3) identified a critical role of TG2 in the regulation of EXT1 expression and the downstream signaling, such as HS biosynthesis, partly through TGF-β1 activation-dependent pathways. The development of both irreversible and reversible inhibitors of TG2 with high efficiency and substrate selectivity has achieved great success in past studies [95]. Translational research on TG2-targeted therapy is expected to aid the development of preventative treatments for HCC by targeting liver CSCs. Clinical trials have already shown that orally administered ACR is well tolerated in the treatment of HCC [96]. Therefore, ACR holds considerable clinical promise as a novel TG2 inhibitor for the treatment of TG2-driven diseases.

## Supplementary information

Figure S1

Figure S2

Figure S3

Figure S4

Figure S5

Figure S6

Figure S7

Figure S8

Table S1

Table S2

Table S3

Table S4

Table S5

Original data for western blots

reproducibility checklist

Authorship confirmation document

## Data Availability

All relevant data were included within the article and online supplementary material. Further inquiries can be directed to the corresponding author.
