# Supplementary material for: Targeting transglutaminase 2 mediated exostosin glycosyltransferase 1 signaling in liver cancer stem cells with acyclic retinoid
Source: Cell Death Dis. 2023 Jun 13;14(6):358. doi: 10.1038/s41419-023-05847-4 (PMC10261105; doi:10.1038/s41419-023-05847-4)
Supplement: Supplementary file 1 — Figure S1 [file 41419_2023_5847_MOESM1_ESM.docx]

**Fig. S1.** **Synthesis of H_2_N-ACR.** Oxidation of farnesyl acetate by a previously reported method (1) using selenium dioxide provided the hydroxy-farnesyl acetate **1**. After protection of the hydroxyl group, reduction of the silyloxy–acetate **2** and subsequent MnO_2_ oxidation provided the aldehyde **3**. Condensation of **3** with the phosphonate **4** gave the silyloxy–ester **5**, which was treated with acid and the resulting alcohol was oxidized with MnO_2_ to yield the formyl-ester **6**. This was then converted into oxime **7** and subjected to reduction (2) with NaBH_4_ in the presence of MoO_3_ to give the amino–ester **8**, accompanied by dimeric secondary amine **9**. Basic hydrolysis of **8** and purification by ion-exchange chromatography generated the desired H_2_N-ACR.

**Supplementary References**

1. Wada A, Wang F, Suhara Y, Yamano Y, Okitsu T, Nakagawa K, et al. Efficient synthesis and biological evaluation of demethyl geranylgeranoic acid derivatives. Bioorg Med Chem. 2010;18(16):5795-806.

2. Ipaktschi J. Reduktion von Oximen mit Natriumboranat in Gegenwart von Übergangsmetallverbindungen. Chem Ber. 1984;117:856–8.
