# Supplementary material for: Targeting transglutaminase 2 mediated exostosin glycosyltransferase 1 signaling in liver cancer stem cells with acyclic retinoid
Source: Cell Death Dis. 2023 Jun 13;14(6):358. doi: 10.1038/s41419-023-05847-4 (PMC10261105; doi:10.1038/s41419-023-05847-4)
Supplement: Supplementary file 2 — Figure S2 [file 41419_2023_5847_MOESM2_ESM.docx]

**Fig. S2. Effects of ACR and vitamin K2 analog on the conformation change of TG2.** (*A*) Recombinant human TG2 was incubated with DMSO or 100 μM ACR in the absence or presence of GTP with increasing concentrations for 2 h at room temperature and then subjected to nondenaturing electrophoresis. The protein bands were visualized with silver staining. (*B*) Chemical structures of ACR, ACR-23, ACR-55, and SVK30. Recombinant human TG2 was incubated (*C*) with 100 μM ACR, or 100 μM of the inactive derivatives of ACR (ACR-23 and ACR-55), or (*D*) with DMSO, 100 μM GTP, 5 mM CaCl_2_, and 100 μM ACR or SVK30 in the absence or presence of 100 μM GTP for 1-2 h at room temperature and then subjected to nondenaturing electrophoresis. The protein bands were visualized with silver staining.
