# Supplementary material for: Targeting transglutaminase 2 mediated exostosin glycosyltransferase 1 signaling in liver cancer stem cells with acyclic retinoid
Source: Cell Death Dis. 2023 Jun 13;14(6):358. doi: 10.1038/s41419-023-05847-4 (PMC10261105; doi:10.1038/s41419-023-05847-4)
Supplement: Supplementary file 3 — Figure S3 [file 41419_2023_5847_MOESM3_ESM.docx]

**
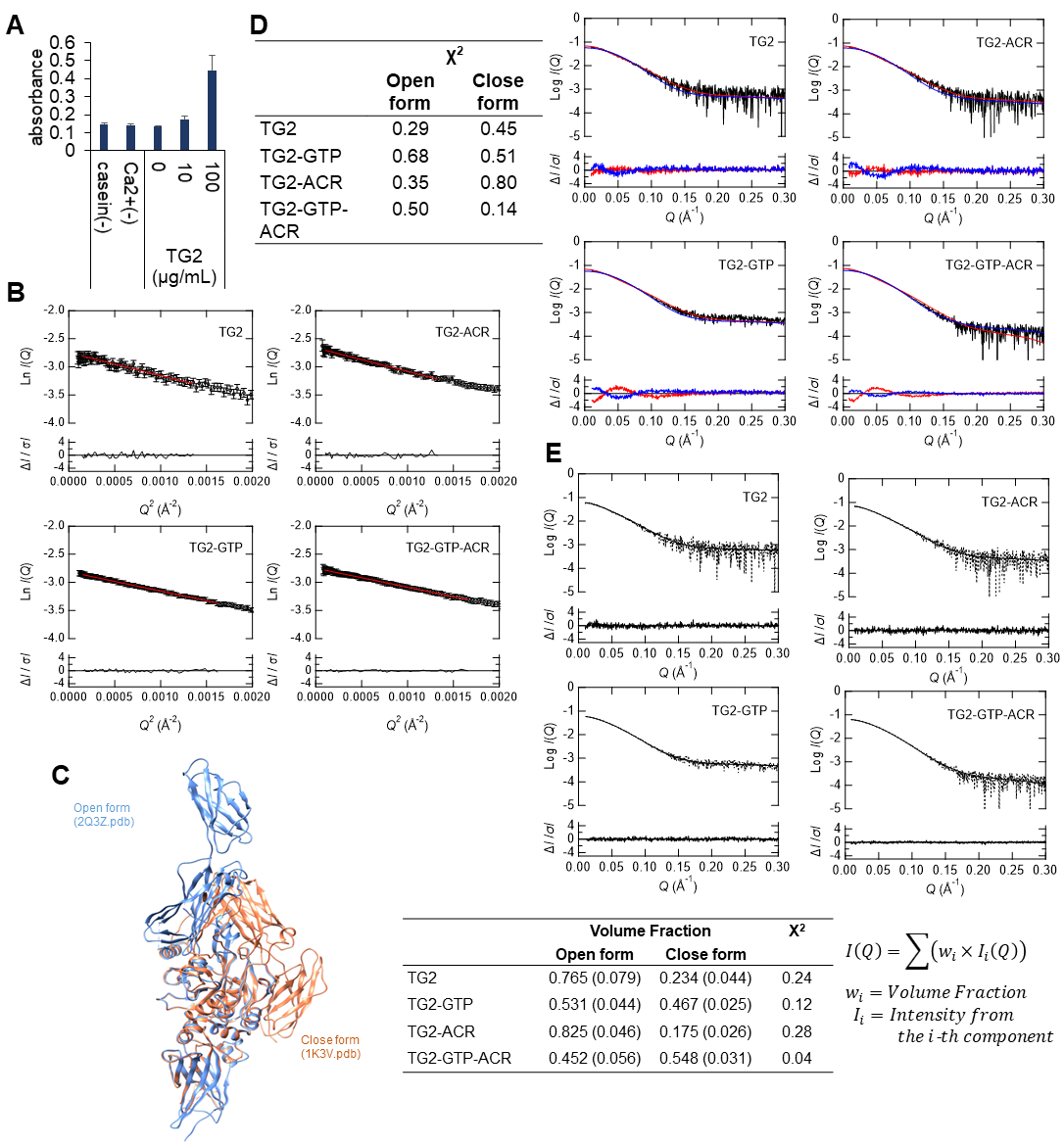
**

**Fig. S3. Effect of ACR and GTP on the conformational structure of TG2 measured with SEC-SAXS.** (*A*) Verification of the transamidase activity of in-house purified recombinant human TG2. 5BAPA incorporation onto the casein-coated plate in the presence of 5 mM CaCl_2_ and increasing concentrations of TG2 for 1 h at 37℃ was examined as an indicator of TG2 transamidase activity. 5BAPA incorporation without casein coating (Casein–) or TG2 (TG2–) was used as the negative control. The data are presented as the mean ± SD. (*B*) (Top) Guinier plots of SAXS intensities measured under four different conditions, where the open black circles with error bars represent the experimental curves and the solid red lines display the results of the linear approximation. (Bottom) Residuals of the linear approximation are plotted. (*C*) Two crystal structures of TG2 already reported. These models were illustrated using Chimera. (*D*) (Left) The χ^2^ values on the following comparisons calculated with CRYSOL are summarized in the table. (Right; Top) Comparison between the four experimental SAXS curves and the theoretical SAXS curves calculated with CRYSOL from the Open or Close form, respectively. The black, red, and blue lines indicate the experimental curve, the theoretical curve from the Open form, and the theoretical curve from the Close form, respectively. (Right; Bottom) Residuals between the experimental SAXS curves and the theoretical SAXS curves calculated from each form. (*E*) Volume Fraction analysis using the experimental data combined with open and closed forms of TG2 calculated with OLIGOMER. (Upper; Top) The dotted line and solid line show the experimental SAXS curve and the theoretical synthesized SAXS curve calculated with OLIGOMER, respectively. (Upper; Bottom) Residuals obtained between the experimental and theoretical curves. (Lower) Volume fractions of open and closed forms and the χ^2^ values for each condition calculated with OLIGOMER are summarized in the table.
