# Supplementary material for: Targeting transglutaminase 2 mediated exostosin glycosyltransferase 1 signaling in liver cancer stem cells with acyclic retinoid
Source: Cell Death Dis. 2023 Jun 13;14(6):358. doi: 10.1038/s41419-023-05847-4 (PMC10261105; doi:10.1038/s41419-023-05847-4)
Supplement: Supplementary file 4 — Figure S4 [file 41419_2023_5847_MOESM4_ESM.docx]

**
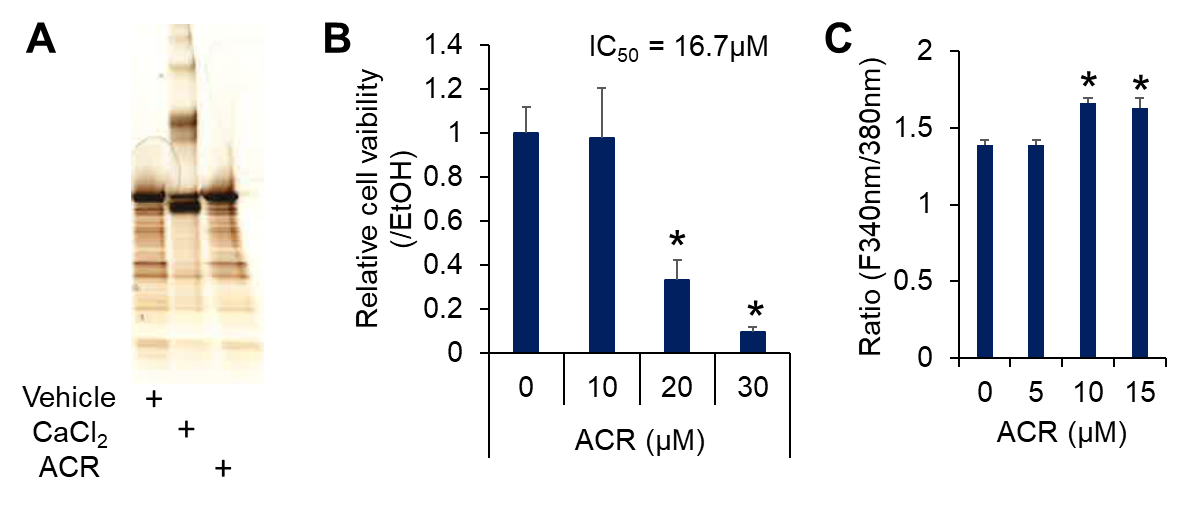
**

**Fig. S4. Effects of CaCl_2_ and ACR on the conformation structure of TG2.** (*A*) Recombinant human TG2 was incubated with EtOH, 5 mM CaCl_2_ or 100 μM ACR for 2 h at room temperature. The proteins were boiled at 98 ℃ for 10 min and subjected to SDS-PAGE. The protein bands were visualized with silver staining. (*B*) Dose-dependent effect of ACR on the cell viability of JHH7 cells. The cells were treated with ACR for 24 h at the indicated concentrations. The half-maximal inhibitory concentration (IC_50_) was determined using the formula: 10^(LOG(A/B)*(50-C)/(D-C)+LOG(B)), where A represents the high concentration that encompasses 50% of inhibition, B represents the low concentration that encompasses 50% of inhition, C represents the inhibition rate at concentration B, and D represents the inhibition rate at concentration A. (*C*) Effect of ACR on intracellular CaCl_2_ concentrations in JHH7 cells. The cells were treated with increasing concentrations of ACR for 4 h and intracellular CaCl_2_ concentration was measured using Fura-2-AM fluorescence and presented as the ratio of F340/F380. The data are presented as the mean ± SD; **P* < 0.05, Student’s *t*-test.
