# Supplementary material for: Targeting transglutaminase 2 mediated exostosin glycosyltransferase 1 signaling in liver cancer stem cells with acyclic retinoid
Source: Cell Death Dis. 2023 Jun 13;14(6):358. doi: 10.1038/s41419-023-05847-4 (PMC10261105; doi:10.1038/s41419-023-05847-4)
Supplement: Supplementary file 5 — Figure S5 [file 41419_2023_5847_MOESM5_ESM.docx]

**Fig. S5. Effect of ACR on intracellular transamidase activity of TG2 and apoptosis in HCC cell line Huh7.** Representative immunofluorescence staining for 5BAPA (red) and clCasp3 (green) of Huh7 cells treated with with EtOH or ACR 15 μM for 4h. Scale bar, 100 μm.
