# Supplementary material for: Targeting transglutaminase 2 mediated exostosin glycosyltransferase 1 signaling in liver cancer stem cells with acyclic retinoid
Source: Cell Death Dis. 2023 Jun 13;14(6):358. doi: 10.1038/s41419-023-05847-4 (PMC10261105; doi:10.1038/s41419-023-05847-4)
Supplement: Supplementary file 6 — Figure S6 [file 41419_2023_5847_MOESM6_ESM.docx]

**Fig. S6. Correlation of TG2 with liver tumorigenesis and effect of ACR on in vivo transglutaminase activity.** (*A*) Effect of NC9 on the gene expression of liver CSC marker *MYCN* in JHH7 cells (3). The cells were treated with DMSO or 10 μM NC9 for 4 h. The data are presented as the mean ± SD; **P* < 0.05, Student’s *t*-test. (*B*) The protein expression of liver progenitor marker pan-cytokeratin and TG2 in the livers of DEN-induced hepatic tumorigenesis mice (4). Mice were given tap water containing 40 ppm of DEN for the first two weeks and fed with basal diet alone (DEN group) and basal diet containing 0.03% ACR (DEN-0.03ACR group) or 0.06% ACR (DEN-0.06ACR group). The group fed with basal diet containing 0.06% ACR alone without DEN treatment (0.06ACR group) were used as the negative control. The stars indicate the non-specific bands. (*C*) Effect of ACR on in vivo transamidase activity of TG2 in the liver of LPS-challenged mice. Schematic overview (*Upper*) and representative immunofluorescence staining for 5BAPA (*Lower*) of the LPS-induced inflammatory livers. Mice were intraperitoneally injected with 10 mg/kg LPS for 24 h. ACR was dissolved in a mixture of DMSO and corn oil and then were intraperitoneally injected at 0.1 mg/kg body weight 30 min before LPS treatment. Thirty min before sacrifice, mice were intraperitoneally injected with 100 mg/kg 5BAPA. Scale bar, 100 μm.

**Supplementary References**

3. Qin XY, Suzuki H, Honda M, Okada H, Kaneko S, Inoue I, et al. Prevention of hepatocellular carcinoma by targeting MYCN-positive liver cancer stem cells with acyclic retinoid. Proc Natl Acad Sci U S A. 2018;115(19):4969-74.

4. Qin XY, Tatsukawa H, Hitomi K, Shirakami Y, Ishibashi N, Shimizu M, et al. Metabolome Analyses Uncovered a Novel Inhibitory Effect of Acyclic Retinoid on Aberrant Lipogenesis in a Mouse Diethylnitrosamine-Induced Hepatic Tumorigenesis Model. Cancer Prev Res. 2016;9(3):205-14.
