# Supplementary material for: Targeting transglutaminase 2 mediated exostosin glycosyltransferase 1 signaling in liver cancer stem cells with acyclic retinoid
Source: Cell Death Dis. 2023 Jun 13;14(6):358. doi: 10.1038/s41419-023-05847-4 (PMC10261105; doi:10.1038/s41419-023-05847-4)
Supplement: Supplementary file 7 — Figure S7 [file 41419_2023_5847_MOESM7_ESM.docx]

**Fig. S7. Proteome analysis of the molecular targets of TG2 in liver CSC.** (*A*) Comparison of upregulated proteins upon treatment with NC9 or ACR in EpCAM+ cells and by TG2 knockdown with shTG2 in JHH7 cells, and downregulated proteins between EpCAM+ and EpCAM– cells. Five common proteins were defined. (*B*) Quantitative data of EXT1 detected using nLC-MS/MS in sorted EpCAM+ and EpCAM– JHH7 cells treated with vehicle control, 25 μM NC9, or 10 μM ACR for 16 h or shCtl and shTG2-transduced JHH7 cells. (*C*) Gene expression of *LGALS1* as a negative control of the unintegrated genes. Correlation between the gene expression of *TGM2* and *EXT1* (*D*) in HCC tissues of 366 patients obtained from the liver hepatocellular carcinoma TCGA PanCancer RNA-seq database; (*E*) in 25 HCC cell lines in the CCLE database; and (*F*) in 50 HCC liver tissues as assessed by CAGE analysis in a European cohort (5). (*G*) Gene expression of *EXT1* in sorted EpCAM+ and EpCAM- JHH7 cells treated with 25 μM NC9 and/or 10 μM ACR for 4h. The data are presented as the mean ± SD; **P* < 0.05, Student’s *t*-test.

**Supplementary References**

5. Hashimoto K, Suzuki AM, Dos Santos A, Desterke C, Collino A, Ghisletti S, et al. CAGE profiling of ncRNAs in hepatocellular carcinoma reveals widespread activation of retroviral LTR promoters in virus-induced tumors. Genome Res 2015;25**:**1812-24.
