# Supplementary material for: Targeting transglutaminase 2 mediated exostosin glycosyltransferase 1 signaling in liver cancer stem cells with acyclic retinoid
Source: Cell Death Dis. 2023 Jun 13;14(6):358. doi: 10.1038/s41419-023-05847-4 (PMC10261105; doi:10.1038/s41419-023-05847-4)
Supplement: Supplementary file 8 — Figure S8 [file 41419_2023_5847_MOESM8_ESM.docx]

**Fig. S8. Effect of TG2 inhibitor CTM on EXT1-HS signaling in HCC cell lines JHH7 and Huh7.** (*A*) Gene expression of *EXT1* and (*B*) representative immunofluorescence staining for HS in JHH7 cells treated with PBS or 500 μM CTM for 16 h. (*C*) Gene expression of *EXT1* and (*D*) representative immunofluorescence staining for HS in Huh7 cells treated with PBS or 250 μM CTM for 16 h. **P* < 0.05, Student’s t-test. Scale bar, 100 μm.
