# Supplementary material for: Targeting transglutaminase 2 mediated exostosin glycosyltransferase 1 signaling in liver cancer stem cells with acyclic retinoid
Source: Cell Death Dis. 2023 Jun 13;14(6):358. doi: 10.1038/s41419-023-05847-4 (PMC10261105; doi:10.1038/s41419-023-05847-4)
Supplement: Supplementary file 9 — Table S1 [file 41419_2023_5847_MOESM9_ESM.docx]

| **A. Sample details** | | | | |
| --- | --- | --- | --- | --- |
|  | TG2 | TG2-GTP | TG2-ACR | TG2-GTP-ACR |
| Organism | *Homo sapiens* | | | |
| Source | *Purification from E. coli KRX strain* | | | |
| Uniprot ID | P21980 | | | |
| Extinction coefficient (A_280nm_, Abs _0.1%_ (w/v)) | 1.369 | | | |
| Partial specific volume (cm^3^ g^-1^) | 0.735 | | | |
| scattering contrast (cm^-2^) | 2.742×10^10^ | | | |
| Molar mass from chemical composition (Da) | 77,258 | | | |
| Initial concentration, for Injection (mg mL^-1^) | 5.6 | | | |
| Injection Volume (mL) | 0.15 | | | |
| Concentration, (mg mL^-1^) | 0.503-1.174 | 0.550-1.477 | 0.300-1.154 | 0.754-1.573 |
| Concentration method | UV-Vis. spectroscopy | | | |
| Solvent composition | 50 mM HEPES, 100 mM NaCl, 1 mM EDTA, 5 mM DTT, 5 % Glycerol, pH 7.0 | | | |
| **B. SAS data collection parameters** | | | | |
| Source, instrument | Photon Factory, BL-10C | | | |
| Wavelength (Å) | 1.213 | | | |
| Camera Length (mm) | 2,008 | | | |
| Beam geometry (mm) | V0.35 × H0.55, Bent cylindrical mirror + 2 slits + 1 pinhole | | | |
| *q*-measurement range (Å^-1^) | 0.0099-0.3053 | 0.0107-0.3373 | 0.0086-0.3373 | 0.0090-0.3373 |
| Absolute scaling method | Comparison with scattering from pure H_2_O | | | |
| Basis for normalization to constant counts | Normalized to incident intensity by µ ion chamber | | | |
| Method for monitoring radiation damage | data frame-by-frame comparison | | | |
| Expo. time, No. of image | 20 sec, 285 | | 20 sec, 642 | |
| path length (mm) | 1.0 | | | |
| Sample temperature (K) | 293 | | | |
| **C. Software employed for SAS data reduction, analysis and interpretation** | | | | |
| SAS data processing | *SAngler, Serial Analyzer* | | | |
| Calculation of contrast and PSV values | *MULCh* | | | |
| Guinier analysis | *AUTORG* | | | |
| Calculation of theoretical scattering curves | *CRYSOL* | | | |
| Calculation of the volume fractions of each component in the mixture | *OLIGOMER* | | | |
| **D. Structural parameters** | | | | |
| Guinier Analysis |  |  |  |  |
| *I*(0) (cm^-1^) | 0.0641 +/- 0.0007 | 0.0602 +/- 0.0002 | 0.0699 +/- 0.0004 | 0.0640 +/- 0.0001 |
| Molar mass from Absolute Scale (Da) | 94,890 | 88,960 | 103,786 | 94,890 |
| *R*_g_ (Å) | 35.0 +/- 0.8 | 32.2 +/- 0.2 | 35.5 +/- 1.0 | 31.8 +/- 0.2 |
| *Q*-range (Å^-1^)  (*Q*×*R*_g_) | 0.0120-0.0368  (0.42-1.29) | 0.0124-0.0402  (0.40-1.30) | 0.0103-0.0364  (0.36-1.29) | 0.0098-0.0407  (0.31-1.29) |

**Table S1. SAS data acquisition, sample details, data analysis, and software used.**
