# Supplementary material for: Targeting transglutaminase 2 mediated exostosin glycosyltransferase 1 signaling in liver cancer stem cells with acyclic retinoid
Source: Cell Death Dis. 2023 Jun 13;14(6):358. doi: 10.1038/s41419-023-05847-4 (PMC10261105; doi:10.1038/s41419-023-05847-4)
Supplement: Supplementary file 14 — Original data for western blots [file 41419_2023_5847_MOESM14_ESM.docx]

**Original western blot data 1.** Related to Figure 1C.

**Original western blot data 2.** Related to Figure 3H.

**Original western blot data 3.** Related to Figure S5B.

**Original western blot data 4.** Related to Figure 4E.
