# Supplementary material for: Targeting transglutaminase 2 mediated exostosin glycosyltransferase 1 signaling in liver cancer stem cells with acyclic retinoid
Source: Cell Death Dis. 2023 Jun 13;14(6):358. doi: 10.1038/s41419-023-05847-4 (PMC10261105; doi:10.1038/s41419-023-05847-4)
Supplement: Supplementary file 16 — Authorship confirmation document [file 41419_2023_5847_MOESM16_ESM.pdf]

XianYang Qin

**差出人:**	Yutaka Furutani <yfurutani@jikei.ac.jp>
**送信日時:**	2023年4月26日水曜日 11:23
**宛先:**	XianYang Qin
**件名:**	Re: Your response is NEEDED

Dear Qin-san,

I agree on the authorship.

Best,
Furutani

On 2023/04/26 11:08, XianYang Qin wrote:

Dear all,

The ACR-TG2 paper is currently undergoing the final quality check in Cell Death & Disease, and I need your confirmation on the authorships.

The most current author list is as follows:
Xian-Yang Qin, Yutaka Furutani, Kento Yonezawa, Nobutaka Shimizu, Miyuki Kato-Murayama, Mikako Shirouzu, Yali Xu, Yumiko Yamano, Akimori Wada, Luc Gailhouste, Rajan Shrestha, Masataka Takahashi, Jeffrey W. Keillor, Ting Su, Wenkui Yu, Shinya Fujii, Hiroyuki Kagechika, Naoshi Dohmae, Yohei Shirakami, Masahito Shimizu, Takahiro Masaki, Tomokazu Matsuura, Harukazu Suzuki

As requested by the journal, I have removed Kojima-sensei from the author list. Alternatively, a footnote has been added to the title to highlight that "This manuscript is dedicated to the memory of Dr. Soichi Kojima (1961-2019)".

Please reply to this email with your agreement on the authorship. I apologize for any inconvenience. You may simply reply with a single word. The most current version of the manuscript is attached at the end of this email.

Best regards,
Xian-Yang

Xian-Yang QIN, Ph.D.
Laboratory for Cellular Function Conversion Technology,
RIKEN Center for Integrative Medical Sciences
1-7-22 Suehiro, Tsurumi, Yokohama, Kanagawa 230-0045, Japan
Tel. +81-(0)50-3502-3731; Fax +81-45-503-9216
E-mail address: xyqin@riken.jp

From: cddisease@springernature.com <cddisease@springernature.com>
Sent: Tuesday, April 25, 2023 11:38 PM
To: XianYang Qin <xyqin@riken.jp>
Subject: CDDIS-22-1027RRR Initial Quality Check

Dear Dr Qin,

In checking in your manuscript submitted to Cell Death & Disease it has come to our attention that the following must be addressed before we can proceed.

1. It has come to our attention that your most recent author list differs from the one in your original submission. Please request agreement from all authors including additions and deletions, these can be collected in the following way:

Email your co-authors with the final author list, and ask them to reply to your email confirming that they agree to the way it is presented. Once you have collected these replies, please combine all of the co-authors' email responses in one document and upload this file to your submission.

2. In checking your manuscript, we have noticed that the author list in your article file differs from your submission. Please could you ensure that all authors are included in both your article file and your online submission.

3. Please make sure you include a data availability statement in your Article file under its own subheading.

Your paper is now available for you to edit, you may access via the following link:

https://mts-cddis.nature.com/cgi-bin/main.plex?el=A5CA3XVP3D2BUIH7F5A9ftdobz29akaW4A60jLYOwQCwZ

(Press/Click on the above link to be automatically sent to the web page.)

Please make the correction(s) as specified above and resubmit your paper following the same steps as before.

If you have any questions please feel free to contact us.

Sincerely,

Editorial Office
Cell Death & Disease
cddisease@springernature.com

This email has been sent through the NPG Manuscript Tracking System NY-610A-NPG&MTS

Confidentiality Statement:

This e-mail is confidential and subject to copyright. Any unauthorised use or disclosure of its contents is prohibited. If you have received this email in error please notify our Manuscript Tracking System Helpdesk team at http://platformsupport.nature.com.

Details of the confidentiality and pre-publicity policy may be found here
http://www.nature.com/authors/policies/confidentiality.html

Privacy Policy | Update Profile

--
****

古谷　裕
東京慈恵会医科大学臨床検査医学講座
〒105-8461 東京都港区西新橋３－２５－８
E-mail: yfurutani@jikei.ac.jp
Tel: 03-3433-1111 内線 2291

****

XianYang Qin

**差出人:**	Yonezawa Kento <yonezawa.kento@ms.naist.jp>
**送信日時:**	2023年4月26日水曜日 12:59
**宛先:**	XianYang Qin
**件名:**	Re: Your response is NEEDED

Dear Qin san,

I agree with you.

Is it possible to change my affiliation as follows?
(before) Center for Digital Green-Innovation,→
(after) Center for Digital Green-innovation,

I apologize for the inconvenience.

Best Regards,
Kento

//
Kento Yonezawa (Assistant Prof.)
Nara institute of Science and Technology Complex Molecular Systems Laboratory Center for Digital Green-Innovation / Division of Materials Science
8916-5 Takayama-cho, Ikoma, Nara, 630-0192, Japan
e-mail: yonezawa.kento@ms.naist.jp
Tel:+81-743-72-6102
//

2023-04-26 11:08 に XianYang Qin さんは書きました:
> Dear all,
>
> The ACR-TG2 paper is currently undergoing the final quality check in
> Cell Death & Disease, and I need your confirmation on the authorships.
>
>
> The most current author list is as follows:
>
> Xian-Yang Qin, Yutaka Furutani, Kento Yonezawa, Nobutaka Shimizu,
> Miyuki Kato-Murayama, Mikako Shirouzu, Yali Xu, Yumiko Yamano, Akimori

> Wada, Luc Gailhouste, Rajan Shrestha, Masataka Takahashi, Jeffrey W.
> Keillor, Ting Su, Wenkui Yu, Shinya Fujii, Hiroyuki Kagechika, Naoshi
> Dohmae, Yohei Shirakami, Masahito Shimizu, Takahiro Masaki, Tomokazu
> Matsuura, Harukazu Suzuki
>
> As requested by the journal, I have removed Kojima-sensei from the
> author list. Alternatively, a footnote has been added to the title to
> highlight that "This manuscript is dedicated to the memory of Dr.
> Soichi Kojima (1961-2019)".
>
> Please reply to this email with your agreement on the authorship. I
> apologize for any inconvenience. You may simply reply with a single
> word. The most current version of the manuscript is attached at the
> end of this email.
>
> Best regards,
>
> Xian-Yang
>
> Xian-Yang QIN, Ph.D.
>
> Laboratory for Cellular Function Conversion Technology,
>
> RIKEN Center for Integrative Medical Sciences
>
> 1-7-22 Suehiro, Tsurumi, Yokohama, Kanagawa 230-0045, Japan
>
> Tel. +81-(0)50-3502-3731; Fax +81-45-503-9216
>
> E-mail address: xyqin@riken.jp
>
> From: cddisease@springernature.com <cddisease@springernature.com>
> Sent: Tuesday, April 25, 2023 11:38 PM
> To: XianYang Qin <xyqin@riken.jp>
> Subject: CDDIS-22-1027RRR Initial Quality Check
>
> Dear Dr Qin,
>
> In checking in your manuscript submitted to Cell Death & Disease it
> has come to our attention that the following must be addressed before
> we can proceed.

>
> 1. It has come to our attention that your most recent author list
> differs from the one in your original submission. Please request
> agreement from all authors including additions and deletions, these
> can be collected in the following way:
>
> Email your co-authors with the final author list, and ask them to
> reply to your email confirming that they agree to the way it is
> presented. Once you have collected these replies, please combine all
> of the co-authors' email responses in one document and upload this
> file to your submission.
>
> 2. In checking your manuscript, we have noticed that the author list
> in your article file differs from your submission. Please could you
> ensure that all authors are included in both your article file and
> your online submission.
>
> 3. Please make sure you include a data availability statement in your
> Article file under its own subheading.
>
> Your paper is now available for you to edit, you may access via the
> following link:
>
> https://mts-cddis.nature.com/cgi-bin/main.plex?el=A5CA3XVP3D2BUIH7F5A9
> ftdobz29akaW4A60jLYOwQCwZ
>
> (Press/Click on the above link to be automatically sent to the web
> page.)
>
> Please make the correction(s) as specified above and resubmit your
> paper following the same steps as before.
>
> If you have any questions please feel free to contact us.
>
> Sincerely,
>
> Editorial Office
> Cell Death & Disease
> cddisease@springernature.com
>
> This email has been sent through the NPG Manuscript Tracking System

> NY-610A-NPG&MTS
>
> _Confidentiality Statement:_
>
> _This e-mail is confidential and subject to copyright. Any
> unauthorised use or disclosure of its contents is prohibited. If you
> have received this email in error please notify our Manuscript
> Tracking System Helpdesk team at __http://platformsupport.nature.com_
> [1]_ . _
>
> _Details of the confidentiality and pre-publicity policy may be found
> here __http://www.nature.com/authors/policies/confidentiality.html_
> [2]_ _
>
> Privacy Policy [3] | Update Profile [4]
>
> Links:
> ------
> [1] http://platformsupport.nature.com
> [2] http://www.nature.com/authors/policies/confidentiality.html
> [3] http://www.nature.com/info/privacy.html
> [4] https://mts-cddis.nature.com

XianYang Qin

**差出人:**	Nobutaka Shimizu <nobutaka.shimizu@kek.jp>
**送信日時:**	2023年4月26日水曜日 13:52
**宛先:**	XianYang Qin
**ＣＣ:**	yfurutani@jikei.ac.jp
**件名:**	Re: Your response is NEEDED

Dear Qin-san,

I also agree with this authorship.
Thank you very much for working toward publication.

Best,
Nobutaka

On Wed, 26 Apr 2023 02:08:27 +0000
XianYang Qin <xyqin@riken.jp> wrote:

> Dear all,
>
> The ACR-TG2 paper is currently undergoing the final quality check in Cell Death & Disease, and I need your confirmation on the authorships.
>
> The most current author list is as follows:
> Xian-Yang Qin, Yutaka Furutani, Kento Yonezawa, Nobutaka Shimizu,
> Miyuki Kato-Murayama, Mikako Shirouzu, Yali Xu, Yumiko Yamano, Akimori
> Wada, Luc Gailhouste, Rajan Shrestha, Masataka Takahashi, Jeffrey W.
> Keillor, Ting Su, Wenkui Yu, Shinya Fujii, Hiroyuki Kagechika, Naoshi
> Dohmae, Yohei Shirakami, Masahito Shimizu, Takahiro Masaki, Tomokazu
> Matsuura, Harukazu Suzuki
>
> As requested by the journal, I have removed Kojima-sensei from the author list. Alternatively, a footnote has been added to the title to highlight that "This manuscript is dedicated to the memory of Dr. Soichi Kojima (1961-2019)".
>
> Please reply to this email with your agreement on the authorship. I apologize for any inconvenience. You may simply reply with a single word. The most current version of the manuscript is attached at the end of this email.
>

> Best regards,
> Xian-Yang
>
> Xian-Yang QIN, Ph.D.
> Laboratory for Cellular Function Conversion Technology, RIKEN Center
> for Integrative Medical Sciences
> 1-7-22 Suehiro, Tsurumi, Yokohama, Kanagawa 230-0045, Japan Tel.
> +81-(0)50-3502-3731; Fax +81-45-503-9216 E-mail address:
> xyqin@riken.jp<mailto:xyqin@riken.jp>
>
> From: cddisease@springernature.com <cddisease@springernature.com>
> Sent: Tuesday, April 25, 2023 11:38 PM
> To: XianYang Qin <xyqin@riken.jp>
> Subject: CDDIS-22-1027RRR Initial Quality Check
>
> Dear Dr Qin,
>
> In checking in your manuscript submitted to Cell Death & Disease it has come to our attention that the following must be addressed before we can proceed.
>
> 1. It has come to our attention that your most recent author list differs from the one in your original submission. Please request agreement from all authors including additions and deletions, these can be collected in the following way:
>
> Email your co-authors with the final author list, and ask them to reply to your email confirming that they agree to the way it is presented. Once you have collected these replies, please combine all of the co-authors' email responses in one document and upload this file to your submission.
>
> 2. In checking your manuscript, we have noticed that the author list in your article file differs from your submission. Please could you ensure that all authors are included in both your article file and your online submission.
>
> 3. Please make sure you include a data availability statement in your Article file under its own subheading.
>
>
> Your paper is now available for you to edit, you may access via the following link:
>
> https://mts-cddis.nature.com/cgi-bin/main.plex?el=A5CA3XVP3D2BUIH7F5A9
> ftdobz29akaW4A60jLYOwQCwZ
>

> (Press/Click on the above link to be automatically sent to the web
> page.)
>
> Please make the correction(s) as specified above and resubmit your paper following the same steps as before.
>
> If you have any questions please feel free to contact us.
>
> Sincerely,
>
> Editorial Office
> Cell Death & Disease
> cddisease@springernature.com<mailto:cddisease@springernature.com>
>
>
>
>
>
> This email has been sent through the NPG Manuscript Tracking System
> NY-610A-NPG&MTS
>
> Confidentiality Statement:
>
> This e-mail is confidential and subject to copyright. Any unauthorised use or disclosure of its contents is prohibited. If you have received this email in error please notify our Manuscript Tracking System Helpdesk team at http://platformsupport.nature.com .
>
> Details of the confidentiality and pre-publicity policy may be found
> here http://www.nature.com/authors/policies/confidentiality.html
>
> Privacy Policy<http://www.nature.com/info/privacy.html> | Update
> Profile<https://mts-cddis.nature.com>

XianYang Qin

差出人: 村山 美幸 <miiyukii@hotmail.com>
送信日時: 2023年4月26日水曜日 19:50
宛先: XianYang Qin
ＣＣ: Mikako Shirouzu
件名: RE: Your response is NEEDED

Dear Dr. Xian-Yang Qin
I agree with you.
Best regard,
Miyuki Kato-Murayama

Windows の メール から送信

差出人: XianYang Qin
送信日時: 2023 年 4 月 26 日 11:08
宛先: yfurutani@jikei.ac.jp; yonezawa.kento@ms.naist.jp; nobutaka.shimizu@kek.jp; miyuki.murayama.aa@riken.jp; Mikako Shirouzu; y-yamano@kobepharma-u.ac.jp; w.akimori5139@gmail.com; Luc Gailhouste; rajanshrestha@gmail.com; jkeillor@uottawa.ca; fujiis.chem@tmd.ac.jp; kage.chem@tmd.ac.jp; tmasaki@jikei.ac.jp; matsuuratomo@gmail.com; Harukazu Suzuki; dr.suting@smail.nju.edu.cn; yudrnj@nju.edu.cn; Naoshi Dohmae; Yali Xu; shimizu.masahito.j1@f.gifu-u.ac.jp; shirakami.yohei.t3@f.gifu-u.ac.jp; rajanshrestha@ku.edu.np
件名: Your response is NEEDED

Dear all,

The ACR-TG2 paper is currently undergoing the final quality check in Cell Death & Disease, and I need your confirmation on the authorships.

The most current author list is as follows:
Xian-Yang Qin, Yutaka Furutani, Kento Yonezawa, Nobutaka Shimizu, Miyuki Kato-Murayama, Mikako Shirouzu, Yali Xu, Yumiko Yamano, Akimori Wada, Luc Gailhouste, Rajan Shrestha, Masataka Takahashi, Jeffrey W. Keillor, Ting Su, Wenkui Yu, Shinya Fujii, Hiroyuki Kagechika, Naoshi Dohmae, Yohei Shirakami, Masahito Shimizu, Takahiro Masaki, Tomokazu Matsuura, Harukazu Suzuki

As requested by the journal, I have removed Kojima-sensei from the author list. Alternatively, a footnote has been added to the title to highlight that "This manuscript is dedicated to the memory of Dr. Soichi Kojima (1961-2019)".

Please reply to this email with your agreement on the authorship. I apologize for any inconvenience. You may simply reply with a single word. The most current version of the manuscript is attached at the end of this email.

Best regards,
Xian-Yang

Xian-Yang QIN, Ph.D.
Laboratory for Cellular Function Conversion Technology,

RIKEN Center for Integrative Medical Sciences
1-7-22 Suehiro, Tsurumi, Yokohama, Kanagawa 230-0045, Japan
Tel. +81-(0)50-3502-3731; Fax +81-45-503-9216
E-mail address: xyqin@riken.jp

From: cddisease@springernature.com <cddisease@springernature.com>
Sent: Tuesday, April 25, 2023 11:38 PM
To: XianYang Qin <xyqin@riken.jp>
Subject: CDDIS-22-1027RRR Initial Quality Check

Dear Dr Qin,

In checking in your manuscript submitted to Cell Death & Disease it has come to our attention that the following must be addressed before we can proceed.

1. It has come to our attention that your most recent author list differs from the one in your original submission. Please request agreement from all authors including additions and deletions, these can be collected in the following way:

Email your co-authors with the final author list, and ask them to reply to your email confirming that they agree to the way it is presented. Once you have collected these replies, please combine all of the co-authors' email responses in one document and upload this file to your submission.

2. In checking your manuscript, we have noticed that the author list in your article file differs from your submission. Please could you ensure that all authors are included in both your article file and your online submission.

3. Please make sure you include a data availability statement in your Article file under its own subheading.

Your paper is now available for you to edit, you may access via the following link:

https://mts-cddis.nature.com/cgi-bin/main.plex?el=A5CA3XVP3D2BUIH7F5A9ftdobz29akaW4A60jLYOwQCwZ

(Press/Click on the above link to be automatically sent to the web page.)

Please make the correction(s) as specified above and resubmit your paper following the same steps as before.

If you have any questions please feel free to contact us.

Sincerely,

Editorial Office
Cell Death & Disease
cddisease@springernature.com

This email has been sent through the NPG Manuscript Tracking System NY-610A-NPG&MTS

Confidentiality Statement:

This e-mail is confidential and subject to copyright. Any unauthorised use or disclosure of its contents is prohibited. If you have received this email in error please notify our Manuscript Tracking System Helpdesk team at http://platformsupport.nature.com .

Details of the confidentiality and pre-publicity policy may be found here
http://www.nature.com/authors/policies/confidentiality.html

Privacy Policy | Update Profile

XianYang Qin

差出人: Mikako Shirouzu
送信日時: 2023年4月26日水曜日 13:15
宛先: XianYang Qin

件名: Re: Your response is NEEDED

Dear Dr. Qin,

I agree on the authorship.

Best regards,

Mikako Shirouzu

On 2023/04/26 11:08, XianYang Qin wrote:
> Dear all,
>
> The ACR-TG2 paper is currently undergoing the final quality check in
> Cell Death & Disease, and I need your confirmation on the authorships.
>
> The most current author list is as follows:
>
> Xian-Yang Qin, Yutaka Furutani, Kento Yonezawa, Nobutaka Shimizu,
> Miyuki Kato-Murayama, Mikako Shirouzu, Yali Xu, Yumiko Yamano, Akimori
> Wada, Luc Gailhouste, Rajan Shrestha, Masataka Takahashi, Jeffrey W.
> Keillor, Ting Su, Wenkui Yu, Shinya Fujii, Hiroyuki Kagechika, Naoshi
> Dohmae, Yohei Shirakami, Masahito Shimizu, Takahiro Masaki, Tomokazu
> Matsuura, Harukazu Suzuki
>
> As requested by the journal, I have removed Kojima-sensei from the
> author list. Alternatively, a footnote has been added to the title to
> highlight that "This manuscript is dedicated to the memory of Dr.
> Soichi Kojima (1961-2019)".
>
> Please reply to this email with your agreement on the authorship. I
> apologize for any inconvenience. You may simply reply with a single
> word. The most current version of the manuscript is attached at the
> end of this email.
>

> Best regards,
>
> Xian-Yang
>
> Xian-Yang QIN, Ph.D.
>
> Laboratory for Cellular Function Conversion Technology,
>
> RIKEN Center for Integrative Medical Sciences
>
> 1-7-22 Suehiro, Tsurumi, Yokohama, Kanagawa 230-0045, Japan
>
> Tel. +81-(0)50-3502-3731; Fax +81-45-503-9216
>
> E-mail address: xyqin@riken.jp <mailto:xyqin@riken.jp>
>
> *From:*cddisease@springernature.com <cddisease@springernature.com>
> *Sent:* Tuesday, April 25, 2023 11:38 PM
> *To:* XianYang Qin <xyqin@riken.jp>
> *Subject:* CDDIS-22-1027RRR Initial Quality Check
>
> Dear Dr Qin,
>
> In checking in your manuscript submitted to Cell Death & Disease it
> has come to our attention that the following must be addressed before
> we can proceed.
>
> 1. It has come to our attention that your most recent author list
> differs from the one in your original submission. Please request
> agreement from all authors including additions and deletions, these
> can be collected in the following way:
>
> Email your co-authors with the final author list, and ask them to
> reply to your email confirming that they agree to the way it is presented.
> Once you have collected these replies, please combine all of the
> co-authors' email responses in one document and upload this file to
> your submission.
>
> 2. In checking your manuscript, we have noticed that the author list
> in your article file differs from your submission. Please could you
> ensure that all authors are included in both your article file and

> your online submission.
>
> 3. Please make sure you include a data availability statement in your
> Article file under its own subheading.
>
>
> Your paper is now available for you to edit, you may access via the
> following link:
>
> https://mts-cddis.nature.com/cgi-bin/main.plex?el=A5CA3XVP3D2BUIH7F5A9
> ftdobz29akaW4A60jLYOwQCwZ
> <https://mts-cddis.nature.com/cgi-bin/main.plex?el=A5CA3XVP3D2BUIH7F5A
> 9ftdobz29akaW4A60jLYOwQCwZ>
>
> (Press/Click on the above link to be automatically sent to the web
> page.)
>
> Please make the correction(s) as specified above and resubmit your
> paper following the same steps as before.
>
> If you have any questions please feel free to contact us.
>
> Sincerely,
>
> Editorial Office
> Cell Death & Disease
> cddisease@springernature.com <mailto:cddisease@springernature.com>
>
>
>
>
> This email has been sent through the NPG Manuscript Tracking System
> NY-610A-NPG&MTS
>
> /Confidentiality Statement://
>
> /This e-mail is confidential and subject to copyright. Any
> unauthorised use or disclosure of its contents is prohibited. If you
> have received this email in error please notify our Manuscript
> Tracking System Helpdesk team at ///http://platformsupport.nature.com/
> <http://platformsupport.nature.com>/. /

>
> /Details of the confidentiality and pre-publicity policy may be found
> here //http://www.nature.com/authors/policies/confidentiality.html/
> <http://www.nature.com/authors/policies/confidentiality.html>//
>
> Privacy Policy <http://www.nature.com/info/privacy.html> | Update
> Profile <https://mts-cddis.nature.com>
>

XianYang Qin

差出人: Yali Xu
送信日時: 2023年4月26日水曜日 12:45
宛先: XianYang Qin
件名: RE: Your response is NEEDED

I confirm that I am a co-author of the paper. Thank you for reaching out to me and congrats on the publication!

Sincerely,

Yali XU
CFCT
RIKEN Center for Integrative Medical Sciences

From: XianYang Qin <xyqin@riken.jp>
Sent: Wednesday, April 26, 2023 11:08 AM
To: yfurutani@jikei.ac.jp; yonezawa.kento@ms.naist.jp; nobutaka.shimizu@kek.jp; miyuki.murayama.aa@riken.jp; Mikako Shirouzu <mikako.shirouzu@riken.jp>; y-yamano@kobepharma-u.ac.jp; w.akimori5139@gmail.com; Luc Gailhouste <luc.gailhouste@riken.jp>; rajanshrestha@gmail.com; jkeillor@uottawa.ca; fujiis.chem@tmd.ac.jp; kage.chem@tmd.ac.jp; tmasaki@jikei.ac.jp; matsuuratomo@gmail.com; Harukazu Suzuki <harukazu.suzuki@riken.jp>; dr.suting@smail.nju.edu.cn; yudrnj@nju.edu.cn; Naoshi Dohmae <dohmae@riken.jp>; Yali Xu <yali.xu@riken.jp>; shimizu.masahito.j1@f.gifu-u.ac.jp; shirakami.yohei.t3@f.gifu-u.ac.jp; rajanshrestha@ku.edu.np
Subject: Your response is NEEDED

Dear all,

The ACR-TG2 paper is currently undergoing the final quality check in Cell Death & Disease, and I need your confirmation on the authorships.

The most current author list is as follows:
Xian-Yang Qin, Yutaka Furutani, Kento Yonezawa, Nobutaka Shimizu, Miyuki Kato-Murayama, Mikako Shirouzu, Yali Xu, Yumiko Yamano, Akimori Wada, Luc Gailhouste, Rajan Shrestha, Masataka Takahashi, Jeffrey W. Keillor, Ting Su, Wenkui Yu, Shinya Fujii, Hiroyuki Kagechika, Naoshi Dohmae, Yohei Shirakami, Masahito Shimizu, Takahiro Masaki, Tomokazu Matsuura, Harukazu Suzuki

As requested by the journal, I have removed Kojima-sensei from the author list. Alternatively, a footnote has been added to the title to highlight that "This manuscript is dedicated to the memory of Dr. Soichi Kojima (1961-2019)".

Please reply to this email with your agreement on the authorship. I apologize for any inconvenience. You may simply reply with a single word. The most current version of the manuscript is attached at the end of this email.

Best regards,
Xian-Yang

Xian-Yang QIN, Ph.D.
Laboratory for Cellular Function Conversion Technology,
RIKEN Center for Integrative Medical Sciences
1-7-22 Suehiro, Tsurumi, Yokohama, Kanagawa 230-0045, Japan
Tel. +81-(0)50-3502-3731; Fax +81-45-503-9216
E-mail address: xyqin@riken.jp

From: cddisease@springernature.com <cddisease@springernature.com>
Sent: Tuesday, April 25, 2023 11:38 PM
To: XianYang Qin <xyqin@riken.jp>
Subject: CDDIS-22-1027RRR Initial Quality Check

Dear Dr Qin,

In checking in your manuscript submitted to Cell Death & Disease it has come to our attention that the following must be addressed before we can proceed.

1. It has come to our attention that your most recent author list differs from the one in your original submission. Please request agreement from all authors including additions and deletions, these can be collected in the following way:

Email your co-authors with the final author list, and ask them to reply to your email confirming that they agree to the way it is presented. Once you have collected these replies, please combine all of the co-authors' email responses in one document and upload this file to your submission.

2. In checking your manuscript, we have noticed that the author list in your article file differs from your submission. Please could you ensure that all authors are included in both your article file and your online submission.

3. Please make sure you include a data availability statement in your Article file under its own subheading.

Your paper is now available for you to edit, you may access via the following link:

https://mts-cddis.nature.com/cgi-bin/main.plex?el=A5CA3XVP3D2BUIH7F5A9ftdobz29akaW4A60jLYOwQCwZ

(Press/Click on the above link to be automatically sent to the web page.)

Please make the correction(s) as specified above and resubmit your paper following the same steps as before.

If you have any questions please feel free to contact us.

Sincerely,

Editorial Office
Cell Death & Disease
cddisease@springernature.com

This email has been sent through the NPG Manuscript Tracking System NY-610A-NPG&MTS

Confidentiality Statement:

This e-mail is confidential and subject to copyright. Any unauthorised use or disclosure of its contents is prohibited. If you have received this email in error please notify our Manuscript Tracking System Helpdesk team at http://platformsupport.nature.com .

Details of the confidentiality and pre-publicity policy may be found here http://www.nature.com/authors/policies/confidentiality.html

Privacy Policy | Update Profile

XianYang Qin

**差出人:**	Yumiko Yamano <y-yamano@kobepharma-u.ac.jp>
**送信日時:**	2023年4月26日水曜日 18:09
**宛先:**	XianYang Qin
**件名:**	Re: Your response is NEEDED

Dear Dr. Xian-Yang QIN

I agree with your suggestion.

Best regard,

Yumiko Yamano
Kobe Pharmaceutical University

> Dear all,
>
> The ACR-TG2 paper is currently undergoing the final quality check in Cell Death & Disease, and I need your confirmation on the authorships.
>
> The most current author list is as follows:
>
> Xian-Yang Qin, Yutaka Furutani, Kento Yonezawa, Nobutaka Shimizu, Miyuki Kato-Murayama, Mikako Shirouzu, Yali Xu, Yumiko Yamano, Akimori Wada, Luc Gailhouste, Rajan Shrestha, Masataka Takahashi, Jeffrey W. Keillor, Ting Su, Wenkui Yu, Shinya Fujii, Hiroyuki Kagechika, Naoshi Dohmae, Yohei Shirakami, Masahito Shimizu, Takahiro Masaki, Tomokazu Matsuura, Harukazu Suzuki
>
> As requested by the journal, I have removed Kojima-sensei from the author list. Alternatively, a footnote has been added to the title to highlight that "This manuscript is dedicated to the memory of Dr. Soichi Kojima (1961-2019)".
>
> Please reply to this email with your agreement on the authorship. I apologize for any inconvenience. You may simply reply with a single word. The most current version of the manuscript is attached at the end of this email.

Best regards,

Xian-Yang

Xian-Yang QIN, Ph.D.

Laboratory for Cellular Function Conversion Technology,

RIKEN Center for Integrative Medical Sciences

1-7-22 Suehiro, Tsurumi, Yokohama, Kanagawa 230-0045, Japan

Tel. +81-(0)50-3502-3731; Fax +81-45-503-9216

E-mail address: xyqin@riken.jp

From: cddisease@springernature.com <cddisease@springernature.com>
Sent: Tuesday, April 25, 2023 11:38 PM
To: XianYang Qin <xyqin@riken.jp>
Subject: CDDIS-22-1027RRR Initial Quality Check

Dear Dr Qin,

In checking in your manuscript submitted to Cell Death & Disease it has come to our attention that the following must be addressed before we can proceed.

1. It has come to our attention that your most recent author list differs from the one in your original submission. Please request agreement from all authors including additions and deletions, these can be collected in the following way:

Email your co-authors with the final author list, and ask them to reply to your email confirming that they agree to the way it is presented. Once you have collected these replies, please combine all of the co-authors' email responses in one document and upload this file to your submission.

2. In checking your manuscript, we have noticed that the author list in your article file differs from your submission. Please could you ensure that all authors are included in both your article file and your online submission.

3. Please make sure you include a data availability statement in your Article file under its own subheading.

Your paper is now available for you to edit, you may access via the following link:

https://mts-cddis.nature.com/cgi-bin/main.plex?el=A5CA3XVP3D2BUIH7F5A9ftdobz29akaW4A60jLYOwQCwZ

(Press/Click on the above link to be automatically sent to the web page.)

Please make the correction(s) as specified above and resubmit your paper following the same steps as before.

If you have any questions please feel free to contact us.

Sincerely,

Editorial Office
Cell Death & Disease
cddisease@springernature.com

XianYang Qin

**差出人:**	和田昭盛 <w.akimori5139@gmail.com>
**送信日時:**	2023年4月26日水曜日 17:59
**宛先:**	XianYang Qin
**ＣＣ:**	yfurutani@jikei.ac.jp; yonezawa.kento@ms.naist.jp; nobutaka.shimizu@kek.jp; miyuki.murayama.aa@riken.jp; Mikako Shirouzu; y-yamano@kobepharma-u.ac.jp; Luc Gailhouste; rajanshrestha@gmail.com; jkeillor@uottawa.ca; fujiis.chem@tmd.ac.jp; kage.chem@tmd.ac.jp; tmasaki@jikei.ac.jp; matsuuratomo@gmail.com; Harukazu Suzuki; dr.suting@smail.nju.edu.cn; yudrnj@nju.edu.cn; Naoshi Dohmae; Yali Xu; shimizu.masahito.j1@f.gifu-u.ac.jp; shirakami.yohei.t3@f.gifu-u.ac.jp; rajanshrestha@ku.edu.np
**件名:**	Re: Your response is NEEDED

Dear Dr. Xian-Yang QIN

I agree with you.

Best regard,

Akimori Wada, Ph. D.

2023 年 4 月 26 日(水) 11:08 XianYang Qin <xyqin@riken.jp>:

> Dear all,
>
> The ACR-TG2 paper is currently undergoing the final quality check in Cell Death & Disease, and I need your confirmation on the authorships.
>
> The most current author list is as follows:
>
> Xian-Yang Qin, Yutaka Furutani, Kento Yonezawa, Nobutaka Shimizu, Miyuki Kato-Murayama, Mikako Shirouzu, Yali Xu, Yumiko Yamano, Akimori Wada, Luc Gailhouste, Rajan Shrestha, Masataka Takahashi, Jeffrey W. Keillor, Ting Su, Wenkui Yu, Shinya Fujii, Hiroyuki Kagechika, Naoshi Dohmae, Yohei Shirakami, Masahito Shimizu, Takahiro Masaki, Tomokazu Matsuura, Harukazu Suzuki
>
> As requested by the journal, I have removed Kojima-sensei from the author list. Alternatively, a footnote has been added to the title to highlight that "This manuscript is dedicated to the memory of Dr. Soichi Kojima (1961-2019)".

Please reply to this email with your agreement on the authorship. I apologize for any inconvenience. You may simply reply with a single word. The most current version of the manuscript is attached at the end of this email.

Best regards,

Xian-Yang

Xian-Yang QIN, Ph.D.

Laboratory for Cellular Function Conversion Technology,

RIKEN Center for Integrative Medical Sciences

1-7-22 Suehiro, Tsurumi, Yokohama, Kanagawa 230-0045, Japan

Tel. +81-(0)50-3502-3731; Fax +81-45-503-9216

E-mail address: xyqin@riken.jp

From: cddisease@springernature.com <cddisease@springernature.com>
Sent: Tuesday, April 25, 2023 11:38 PM
To: XianYang Qin <xyqin@riken.jp>
Subject: CDDIS-22-1027RRR Initial Quality Check

Dear Dr Qin,

In checking in your manuscript submitted to Cell Death & Disease it has come to our attention that the following must be addressed before we can proceed.

1. It has come to our attention that your most recent author list differs from the one in your original submission. Please request agreement from all authors including additions and deletions, these can be collected in the following way:

Email your co-authors with the final author list, and ask them to reply to your email confirming that they agree to the way it is presented. Once you have collected these replies, please combine all of the co-authors' email responses in one document and upload this file to your submission.

2. In checking your manuscript, we have noticed that the author list in your article file differs from your

submission. Please could you ensure that all authors are included in both your article file and your online submission.

3. Please make sure you include a data availability statement in your Article file under its own subheading.

Your paper is now available for you to edit, you may access via the following link:

https://mts-cddis.nature.com/cgi-bin/main.plex?el=A5CA3XVP3D2BUIH7F5A9ftdobz29akaW4A60jLYOwQCwZ

(Press/Click on the above link to be automatically sent to the web page.)

Please make the correction(s) as specified above and resubmit your paper following the same steps as before.

If you have any questions please feel free to contact us.

Sincerely,

Editorial Office
Cell Death & Disease
cddisease@springernature.com

This email has been sent through the NPG Manuscript Tracking System NY-610A-NPG&MTS

Confidentiality Statement:

This e-mail is confidential and subject to copyright. Any unauthorised use or disclosure of its contents is prohibited. If you have received this email in error please notify our Manuscript Tracking System Helpdesk team at http://platformsupport.nature.com .

Details of the confidentiality and pre-publicity policy may be found here http://www.nature.com/authors/policies/confidentiality.html

Privacy Policy | Update Profile

差出人: Luc Gailhouste
送信日時: 2023年4月26日水曜日 11:19
宛先: XianYang Qin; yfurutani@jikei.ac.jp; yonezawa.kento@ms.naist.jp; nobutaka.shimizu@kek.jp; miyuki.murayama.aa@riken.jp; Mikako Shirouzu; y-yamano@kobepharma-u.ac.jp; w.akimori5139@gmail.com; rajanshrestha@gmail.com; jkeillor@uottawa.ca; fujiis.chem@tmd.ac.jp; kage.chem@tmd.ac.jp; tmasaki@jikei.ac.jp; matsuuratomo@gmail.com; Harukazu Suzuki; dr.suting@smail.nju.edu.cn; yudrnj@nju.edu.cn; Naoshi Dohmae; Yali Xu; shimizu.masahito.j1@f.gifu-u.ac.jp; shirakami.yohei.t3@f.gifu-u.ac.jp; rajanshrestha@ku.edu.np
件名: Re: Your response is NEEDED

Qin-san,

Sure, I agree on the authorship.

Congrats again on your success and for all your hard work.

Best,

Luc

--
Luc Gailhouste, Ph.D.
理化学研究所
RIKEN Center for Brain Science
Lab for Brain Development & Disorders
Brain Central Research Building N406, 2-1 Hirosawa, Wako
Saitama 351-0198. JAPAN
Tel: +81-(0)50-3495-0326

From: XianYang Qin <xyqin@riken.jp>
Sent: 26 April 2023 11:08
To: yfurutani@jikei.ac.jp <yfurutani@jikei.ac.jp>; yonezawa.kento@ms.naist.jp <yonezawa.kento@ms.naist.jp>; nobutaka.shimizu@kek.jp <nobutaka.shimizu@kek.jp>; miyuki.murayama.aa@riken.jp <miyuki.murayama.aa@riken.jp>; Mikako Shirouzu <mikako.shirouzu@riken.jp>; y-yamano@kobepharma-u.ac.jp <y-yamano@kobepharma-u.ac.jp>; w.akimori5139@gmail.com <w.akimori5139@gmail.com>; Luc Gailhouste <luc.gailhouste@riken.jp>; rajanshrestha@gmail.com <rajanshrestha@gmail.com>; jkeillor@uottawa.ca <jkeillor@uottawa.ca>; fujiis.chem@tmd.ac.jp <fujiis.chem@tmd.ac.jp>; kage.chem@tmd.ac.jp <kage.chem@tmd.ac.jp>; tmasaki@jikei.ac.jp <tmasaki@jikei.ac.jp>; matsuuratomo@gmail.com <matsuuratomo@gmail.com>; Harukazu Suzuki <harukazu.suzuki@riken.jp>; dr.suting@smail.nju.edu.cn <dr.suting@smail.nju.edu.cn>; yudrnj@nju.edu.cn <yudrnj@nju.edu.cn>; Naoshi Dohmae <dohmae@riken.jp>; Yali Xu <yali.xu@riken.jp>; shimizu.masahito.j1@f.gifu-u.ac.jp <shimizu.masahito.j1@f.gifu-u.ac.jp>;

shirakami.yohei.t3@f.gifu-u.ac.jp <shirakami.yohei.t3@f.gifu-u.ac.jp>; rajanshrestha@ku.edu.np <rajanshrestha@ku.edu.np>
Subject: Your response is NEEDED

Dear all,

The ACR-TG2 paper is currently undergoing the final quality check in Cell Death & Disease, and I need your confirmation on the authorships.

The most current author list is as follows:
Xian-Yang Qin, Yutaka Furutani, Kento Yonezawa, Nobutaka Shimizu, Miyuki Kato-Murayama, Mikako Shirouzu, Yali Xu, Yumiko Yamano, Akimori Wada, Luc Gailhouste, Rajan Shrestha, Masataka Takahashi, Jeffrey W. Keillor, Ting Su, Wenkui Yu, Shinya Fujii, Hiroyuki Kagechika, Naoshi Dohmae, Yohei Shirakami, Masahito Shimizu, Takahiro Masaki, Tomokazu Matsuura, Harukazu Suzuki

As requested by the journal, I have removed Kojima-sensei from the author list. Alternatively, a footnote has been added to the title to highlight that "This manuscript is dedicated to the memory of Dr. Soichi Kojima (1961-2019)".

Please reply to this email with your agreement on the authorship. I apologize for any inconvenience. You may simply reply with a single word. The most current version of the manuscript is attached at the end of this email.

Best regards,
Xian-Yang

Xian-Yang QIN, Ph.D.
Laboratory for Cellular Function Conversion Technology,
RIKEN Center for Integrative Medical Sciences
1-7-22 Suehiro, Tsurumi, Yokohama, Kanagawa 230-0045, Japan
Tel. +81-(0)50-3502-3731; Fax +81-45-503-9216
E-mail address: xyqin@riken.jp

From: cddisease@springernature.com <cddisease@springernature.com>
Sent: Tuesday, April 25, 2023 11:38 PM
To: XianYang Qin <xyqin@riken.jp>
Subject: CDDIS-22-1027RRR Initial Quality Check

Dear Dr Qin,

In checking in your manuscript submitted to Cell Death & Disease it has come to our attention that the following must be addressed before we can proceed.

1. It has come to our attention that your most recent author list differs from the one in your original submission. Please request agreement from all authors including additions and deletions, these can be collected in the following way:

Email your co-authors with the final author list, and ask them to reply to your email confirming that they agree to the way it is presented. Once you have collected these replies, please combine all of the co-authors' email responses in one document and upload this file to your submission.

2. In checking your manuscript, we have noticed that the author list in your article file differs from your submission. Please could you ensure that all authors are included in both your article file and your online submission.

3. Please make sure you include a data availability statement in your Article file under its own subheading.

Your paper is now available for you to edit, you may access via the following link:

https://mts-cddis.nature.com/cgi-bin/main.plex?el=A5CA3XVP3D2BUIH7F5A9ftdobz29akaW4A60jLYOwQCwZ

(Press/Click on the above link to be automatically sent to the web page.)

Please make the correction(s) as specified above and resubmit your paper following the same steps as before.

If you have any questions please feel free to contact us.

Sincerely,

Editorial Office
Cell Death & Disease
cddisease@springernature.com

This email has been sent through the NPG Manuscript Tracking System NY-610A-NPG&MTS

Confidentiality Statement:

This e-mail is confidential and subject to copyright. Any unauthorised use or disclosure of its contents is prohibited. If you have received this email in error please notify our Manuscript Tracking System Helpdesk team at http://platformsupport.nature.com .
Details of the confidentiality and pre-publicity policy may be found here
http://www.nature.com/authors/policies/confidentiality.html
Privacy Policy | Update Profile

XianYang Qin

**差出人:**	Rajan Shrestha <rajanshrestha@ku.edu.np>
**送信日時:**	2023年4月26日水曜日 17:08
**宛先:**	XianYang Qin
**件名:**	Re: Your response is NEEDED

Dear Qin-San,
Thanks and congratulations for your success. You have my agreement on the author list.

Sincerely Yours`
Rajan

On Wed, Apr 26, 2023 at 7:53 AM XianYang Qin <xyqin@riken.jp> wrote:

> Dear all,
>
> The ACR-TG2 paper is currently undergoing the final quality check in Cell Death & Disease, and I need your confirmation on the authorships.
>
> The most current author list is as follows:
>
> Xian-Yang Qin, Yutaka Furutani, Kento Yonezawa, Nobutaka Shimizu, Miyuki Kato-Murayama, Mikako Shirouzu, Yali Xu, Yumiko Yamano, Akimori Wada, Luc Gailhouste, Rajan Shrestha, Masataka Takahashi, Jeffrey W. Keillor, Ting Su, Wenkui Yu, Shinya Fujii, Hiroyuki Kagechika, Naoshi Dohmae, Yohei Shirakami, Masahito Shimizu, Takahiro Masaki, Tomokazu Matsuura, Harukazu Suzuki
>
> As requested by the journal, I have removed Kojima-sensei from the author list. Alternatively, a footnote has been added to the title to highlight that "This manuscript is dedicated to the memory of Dr. Soichi Kojima (1961-2019)".
>
> Please reply to this email with your agreement on the authorship. I apologize for any inconvenience. You may simply reply with a single word. The most current version of the manuscript is attached at the end of this email.
>
> Best regards,

Xian-Yang

Xian-Yang QIN, Ph.D.

Laboratory for Cellular Function Conversion Technology,

RIKEN Center for Integrative Medical Sciences

1-7-22 Suehiro, Tsurumi, Yokohama, Kanagawa 230-0045, Japan

Tel. +81-(0)50-3502-3731; Fax +81-45-503-9216

E-mail address: xyqin@riken.jp

From: cddisease@springernature.com <cddisease@springernature.com>
Sent: Tuesday, April 25, 2023 11:38 PM
To: XianYang Qin <xyqin@riken.jp>
Subject: CDDIS-22-1027RRR Initial Quality Check

Dear Dr Qin,

In checking in your manuscript submitted to Cell Death & Disease it has come to our attention that the following must be addressed before we can proceed.

1. It has come to our attention that your most recent author list differs from the one in your original submission. Please request agreement from all authors including additions and deletions, these can be collected in the following way:

Email your co-authors with the final author list, and ask them to reply to your email confirming that they agree to the way it is presented. Once you have collected these replies, please combine all of the co-authors' email responses in one document and upload this file to your submission.

2. In checking your manuscript, we have noticed that the author list in your article file differs from your submission. Please could you ensure that all authors are included in both your article file and your online submission.

3. Please make sure you include a data availability statement in your Article file under its own subheading.

Your paper is now available for you to edit, you may access via the following link:

https://mts-cddis.nature.com/cgi-

bin/main.plex?el=A5CA3XVP3D2BUIH7F5A9ftdobz29akaW4A60jLYOwQCwZ

(Press/Click on the above link to be automatically sent to the web page.)

Please make the correction(s) as specified above and resubmit your paper following the same steps as before.

If you have any questions please feel free to contact us.

Sincerely,

Editorial Office
Cell Death & Disease
cddisease@springernature.com

This email has been sent through the NPG Manuscript Tracking System NY-610A-NPG&MTS

Confidentiality Statement:

This e-mail is confidential and subject to copyright. Any unauthorised use or disclosure of its contents is prohibited. If you have received this email in error please notify our Manuscript Tracking System Helpdesk team at http://platformsupport.nature.com .

Details of the confidentiality and pre-publicity policy may be found here http://www.nature.com/authors/policies/confidentiality.html

Privacy Policy | Update Profile

XianYang Qin

差出人: MASA TAKAHASHI <t8shikun@gmail.com>
送信日時: 2023年4月27日木曜日 0:34
宛先: XianYang Qin
件名: Re: FW: Your response is NEEDED

秦さゆ

お疲れ様です。
下記確認しました。よろしくお願い致します。

高橋

2023 年 4 月 26 日(水) 17:10 XianYang Qin <xyqin@riken.jp>:

> 高橋さん
>
> お疲れ様です。
>
> メールアドレスを失念してすみません。投稿中の CDDis 論文について Authorship の確認が必要なので、お時間がある時に返事をいただけますと幸いです。
>
> お手数ですが、よろしくお願いいたします。
>
> 秦咸陽
>
> ---
>
> **From:** XianYang Qin
> **Sent:** Wednesday, April 26, 2023 11:08 AM
> **To:** yfurutani@jikei.ac.jp; yonezawa.kento@ms.naist.jp; nobutaka.shimizu@kek.jp; 'miyuki.murayama.aa@riken.jp' <miyuki.murayama.aa@riken.jp>; Mikako Shirouzu <mikako.shirouzu@riken.jp>; y-yamano@kobepharma-u.ac.jp; w.akimori5139@gmail.com; Luc Gailhouste <luc.gailhouste@riken.jp>; rajanshrestha@gmail.com; jkeillor@uottawa.ca; fujiis.chem@tmd.ac.jp; kage.chem@tmd.ac.jp; tmasaki@jikei.ac.jp; matsuuratomo@gmail.com; Harukazu Suzuki <harukazu.suzuki@riken.jp>; dr.suting@smail.nju.edu.cn; yudrnj@nju.edu.cn; Naoshi Dohmae <dohmae@riken.jp>; Yali Xu <yali.xu@riken.jp>; shimizu.masahito.j1@f.gifu-u.ac.jp;

shirakami.yohei.t3@f.gifu-u.ac.jp; rajanshrestha@ku.edu.np
Subject: Your response is NEEDED

Dear all,

The ACR-TG2 paper is currently undergoing the final quality check in Cell Death & Disease, and I need your confirmation on the authorships.

The most current author list is as follows:

Xian-Yang Qin, Yutaka Furutani, Kento Yonezawa, Nobutaka Shimizu, Miyuki Kato-Murayama, Mikako Shirouzu, Yali Xu, Yumiko Yamano, Akimori Wada, Luc Gailhouste, Rajan Shrestha, Masataka Takahashi, Jeffrey W. Keillor, Ting Su, Wenkui Yu, Shinya Fujii, Hiroyuki Kagechika, Naoshi Dohmae, Yohei Shirakami, Masahito Shimizu, Takahiro Masaki, Tomokazu Matsuura, Harukazu Suzuki

As requested by the journal, I have removed Kojima-sensei from the author list. Alternatively, a footnote has been added to the title to highlight that "This manuscript is dedicated to the memory of Dr. Soichi Kojima (1961-2019)".

Please reply to this email with your agreement on the authorship. I apologize for any inconvenience. You may simply reply with a single word. The most current version of the manuscript is attached at the end of this email.

Best regards,

Xian-Yang

Xian-Yang QIN, Ph.D.

Laboratory for Cellular Function Conversion Technology,

RIKEN Center for Integrative Medical Sciences

1-7-22 Suehiro, Tsurumi, Yokohama, Kanagawa 230-0045, Japan

Tel. +81-(0)50-3502-3731; Fax +81-45-503-9216

E-mail address: xyqin@riken.jp

From: cddisease@springernature.com <cddisease@springernature.com>
Sent: Tuesday, April 25, 2023 11:38 PM
To: XianYang Qin <xyqin@riken.jp>
Subject: CDDIS-22-1027RRR Initial Quality Check

Dear Dr Qin,

In checking in your manuscript submitted to Cell Death & Disease it has come to our attention that the following must be addressed before we can proceed.

1. It has come to our attention that your most recent author list differs from the one in your original submission. Please request agreement from all authors including additions and deletions, these can be collected in the following way:

Email your co-authors with the final author list, and ask them to reply to your email confirming that they agree to the way it is presented. Once you have collected these replies, please combine all of the co-authors' email responses in one document and upload this file to your submission.

2. In checking your manuscript, we have noticed that the author list in your article file differs from your submission. Please could you ensure that all authors are included in both your article file and your online submission.

3. Please make sure you include a data availability statement in your Article file under its own subheading.

Your paper is now available for you to edit, you may access via the following link:

https://mts-cddis.nature.com/cgi-bin/main.plex?el=A5CA3XVP3D2BUIH7F5A9ftdobz29akaW4A60jLYOwQCwZ

(Press/Click on the above link to be automatically sent to the web page.)

Please make the correction(s) as specified above and resubmit your paper following the same steps as before.

If you have any questions please feel free to contact us.

Sincerely,

Editorial Office
Cell Death & Disease

cddisease@springernature.com

This email has been sent through the NPG Manuscript Tracking System NY-610A-NPG&MTS

Confidentiality Statement:

This e-mail is confidential and subject to copyright. Any unauthorised use or disclosure of its contents is prohibited. If you have received this email in error please notify our Manuscript Tracking System Helpdesk team at http://platformsupport.nature.com .

Details of the confidentiality and pre-publicity policy may be found here http://www.nature.com/authors/policies/confidentiality.html

Privacy Policy | Update Profile

--

髙橋昌剛
〒1360072
江東区大島 3-12-8-101

TEL:090-5624-5830

差出人: Jeffrey W. Keillor <jkeillor@uottawa.ca>
送信日時: 2023年4月26日水曜日 22:49
宛先: XianYang Qin; yfurutani@jikei.ac.jp; yonezawa.kento@ms.naist.jp; nobutaka.shimizu@kek.jp; miyuki.murayama.aa@riken.jp; Mikako Shirouzu; y-yamano@kobepharma-u.ac.jp; w.akimori5139@gmail.com; Luc Gailhouste; rajanshrestha@gmail.com; fujiis.chem@tmd.ac.jp; kage.chem@tmd.ac.jp; tmasaki@jikei.ac.jp; matsuuratomo@gmail.com; Harukazu Suzuki; dr.suting@smail.nju.edu.cn; yudrnj@nju.edu.cn; Naoshi Dohmae; Yali Xu; shimizu.masahito.j1@f.gifu-u.ac.jp; shirakami.yohei.t3@f.gifu-u.ac.jp; rajanshrestha@ku.edu.np
件名: Re: Your response is NEEDED

Hello,
Thank you for your email.
I understand your situation, but I do not think the *authorship* of a paper should be decided ultimately by the policies of an institution with respect to their email accounts! I would think that if you communicated with the Editor directly, to explain this situation, they would understand and be able to add an author name manually, without requiring the email address.
Regarding the addition of the other authors, I am of course fully in support, as I understand how their assistance was required to complete this fine work.
Respectfully,
Jeffrey Keillor

From: XianYang Qin <xyqin@riken.jp>
Date: Wednesday, 26 April 2023 at 09:35
To: Jeffrey W. Keillor <jkeillor@uottawa.ca>, yfurutani@jikei.ac.jp <yfurutani@jikei.ac.jp>, yonezawa.kento@ms.naist.jp <yonezawa.kento@ms.naist.jp>, nobutaka.shimizu@kek.jp <nobutaka.shimizu@kek.jp>, miyuki.murayama.aa@riken.jp <miyuki.murayama.aa@riken.jp>, Mikako Shirouzu <mikako.shirouzu@riken.jp>, y-yamano@kobepharma-u.ac.jp <y-yamano@kobepharma-u.ac.jp>, w.akimori5139@gmail.com <w.akimori5139@gmail.com>, Luc Gailhouste <luc.gailhouste@riken.jp>, rajanshrestha@gmail.com <rajanshrestha@gmail.com>, fujiis.chem@tmd.ac.jp <fujiis.chem@tmd.ac.jp>, kage.chem@tmd.ac.jp <kage.chem@tmd.ac.jp>, tmasaki@jikei.ac.jp <tmasaki@jikei.ac.jp>, matsuuratomo@gmail.com <matsuuratomo@gmail.com>, Harukazu Suzuki <harukazu.suzuki@riken.jp>, dr.suting@smail.nju.edu.cn <dr.suting@smail.nju.edu.cn>, yudrnj@nju.edu.cn <yudrnj@nju.edu.cn>, Naoshi Dohmae <dohmae@riken.jp>, Yali Xu <yali.xu@riken.jp>, shimizu.masahito.j1@f.gifu-u.ac.jp <shimizu.masahito.j1@f.gifu-u.ac.jp>, shirakami.yohei.t3@f.gifu-u.ac.jp <shirakami.yohei.t3@f.gifu-u.ac.jp>, rajanshrestha@ku.edu.np <rajanshrestha@ku.edu.np>
Subject: RE: Your response is NEEDED

Attention : courriel externe | external email
Dear Jeff,

Thank you for reminding me about this point. The issue is that the journal requires an active email address in the online submission system. In previous submissions to other journals, I was suggested

to add a memorial for Kojima-sensei in the acknowledgements or as a footnote in the manuscript. Initially, I thought that Cell Death & Disease would allow for a different author list in the article file and the online submission system since they did not mention this in our original submission… However, I will contact the journal again to explain this situation.

Another reason for the confirmation of authorship is needed is because new authors were added to prepare the revised manuscript.

Best regards,
Xian-Yang

差出人: Jeffrey W. Keillor
送信日時: 2023 年 4 月 26 日 20:50
宛先: XianYang Qin; yfurutani@jikei.ac.jp; yonezawa.kento@ms.naist.jp; nobutaka.shimizu@kek.jp; miyuki.murayama.aa@riken.jp; Mikako Shirouzu; y-yamano@kobepharma-u.ac.jp; w.akimori5139@gmail.com; Luc Gailhouste; rajanshrestha@gmail.com; fujiis.chem@tmd.ac.jp; kage.chem@tmd.ac.jp; tmasaki@jikei.ac.jp; matsuuratomo@gmail.com; Harukazu Suzuki; dr.suting@smail.nju.edu.cn; yudrnj@nju.edu.cn; Naoshi Dohmae; Yali Xu; shimizu.masahito.j1@f.gifu-u.ac.jp; shirakami.yohei.t3@f.gifu-u.ac.jp; rajanshrestha@ku.edu.np
件名: Re: Your response is NEEDED

Hello,
I don't understand why the journal would ask you to remove the name of Soichi Kojima.
If he was involved in the intellectual framework of this specific study, and contributed to the design of experiments for this specific study, he should be listed as an author. (If someone leaves my group, and their work is published many years later, they are still listed as a co-author.) Surely this is something that we can justify to the journal.
On the other hand, if he had nothing to do with this study, and it was conceived, initiated and completed after his untimely passing, then I can understand removing his name.
Xian-Yang, I think you are in the best position to discern between these two scenarios, and I will approve your decision. I just want to remind you that it is not the journal that decides, it is the authors.
Thank you,
Jeffrey Keillor

From: XianYang Qin <xyqin@riken.jp>
Date: Tuesday, 25 April 2023 at 22:08
To: yfurutani@jikei.ac.jp <yfurutani@jikei.ac.jp>, yonezawa.kento@ms.naist.jp <yonezawa.kento@ms.naist.jp>, nobutaka.shimizu@kek.jp <nobutaka.shimizu@kek.jp>, miyuki.murayama.aa@riken.jp <miyuki.murayama.aa@riken.jp>, Mikako Shirouzu <mikako.shirouzu@riken.jp>, y-yamano@kobepharma-u.ac.jp <y-yamano@kobepharma-u.ac.jp>, w.akimori5139@gmail.com <w.akimori5139@gmail.com>, Luc Gailhouste <luc.gailhouste@riken.jp>, rajanshrestha@gmail.com <rajanshrestha@gmail.com>, Jeffrey W. Keillor <jkeillor@uottawa.ca>, fujiis.chem@tmd.ac.jp <fujiis.chem@tmd.ac.jp>, kage.chem@tmd.ac.jp <kage.chem@tmd.ac.jp>, tmasaki@jikei.ac.jp <tmasaki@jikei.ac.jp>, matsuuratomo@gmail.com <matsuuratomo@gmail.com>, Harukazu Suzuki <harukazu.suzuki@riken.jp>, dr.suting@smail.nju.edu.cn <dr.suting@smail.nju.edu.cn>,

yudrnj@nju.edu.cn <yudrnj@nju.edu.cn>, Naoshi Dohmae <dohmae@riken.jp>, Yali Xu <yali.xu@riken.jp>, shimizu.masahito.j1@f.gifu-u.ac.jp <shimizu.masahito.j1@f.gifu-u.ac.jp>, shirakami.yohei.t3@f.gifu-u.ac.jp <shirakami.yohei.t3@f.gifu-u.ac.jp>, rajanshrestha@ku.edu.np <rajanshrestha@ku.edu.np>
Subject: Your response is NEEDED

Attention : courriel externe | external email
Dear all,

The ACR-TG2 paper is currently undergoing the final quality check in Cell Death & Disease, and I need your confirmation on the authorships.

The most current author list is as follows:
Xian-Yang Qin, Yutaka Furutani, Kento Yonezawa, Nobutaka Shimizu, Miyuki Kato-Murayama, Mikako Shirouzu, Yali Xu, Yumiko Yamano, Akimori Wada, Luc Gailhouste, Rajan Shrestha, Masataka Takahashi, Jeffrey W. Keillor, Ting Su, Wenkui Yu, Shinya Fujii, Hiroyuki Kagechika, Naoshi Dohmae, Yohei Shirakami, Masahito Shimizu, Takahiro Masaki, Tomokazu Matsuura, Harukazu Suzuki

As requested by the journal, I have removed Kojima-sensei from the author list. Alternatively, a footnote has been added to the title to highlight that "This manuscript is dedicated to the memory of Dr. Soichi Kojima (1961-2019)".

Please reply to this email with your agreement on the authorship. I apologize for any inconvenience. You may simply reply with a single word. The most current version of the manuscript is attached at the end of this email.

Best regards,
Xian-Yang

Xian-Yang QIN, Ph.D.
Laboratory for Cellular Function Conversion Technology,
RIKEN Center for Integrative Medical Sciences
1-7-22 Suehiro, Tsurumi, Yokohama, Kanagawa 230-0045, Japan
Tel. +81-(0)50-3502-3731; Fax +81-45-503-9216
E-mail address: xyqin@riken.jp

From: cddisease@springernature.com <cddisease@springernature.com>
Sent: Tuesday, April 25, 2023 11:38 PM
To: XianYang Qin <xyqin@riken.jp>
Subject: CDDIS-22-1027RRR Initial Quality Check

Dear Dr Qin,

In checking in your manuscript submitted to Cell Death & Disease it has come to our attention that the following must be addressed before we can proceed.

1. It has come to our attention that your most recent author list differs from the one in your original submission. Please request agreement from all authors including additions and deletions, these can be collected in the following way:

Email your co-authors with the final author list, and ask them to reply to your email confirming that they agree to the way it is presented. Once you have collected these replies, please combine all of the co-authors' email responses in one document and upload this file to your submission.

2. In checking your manuscript, we have noticed that the author list in your article file differs from

your submission. Please could you ensure that all authors are included in both your article file and your online submission.

3. Please make sure you include a data availability statement in your Article file under its own subheading.

Your paper is now available for you to edit, you may access via the following link:

https://mts-cddis.nature.com/cgi-bin/main.plex?el=A5CA3XVP3D2BUIH7F5A9ftdobz29akaW4A60jLYOwQCwZ

(Press/Click on the above link to be automatically sent to the web page.)

Please make the correction(s) as specified above and resubmit your paper following the same steps as before.

If you have any questions please feel free to contact us.

Sincerely,

Editorial Office
Cell Death & Disease
cddisease@springernature.com

This email has been sent through the NPG Manuscript Tracking System NY-610A-NPG&MTS

Confidentiality Statement:

This e-mail is confidential and subject to copyright. Any unauthorised use or disclosure of its contents is prohibited. If you have received this email in error please notify our Manuscript Tracking System Helpdesk team at http://platformsupport.nature.com .

Details of the confidentiality and pre-publicity policy may be found here http://www.nature.com/authors/policies/confidentiality.html

Privacy Policy | Update Profile

XianYang Qin

差出人: Ting Su <dr.suting@smail.nju.edu.cn>
送信日時: 2023年4月26日水曜日 11:44
宛先: XianYang Qin

件名: 回复:Your response is NEEDED

Qin-sensei,

Thank you very much and congratulations.
I surely agree on the authorships.

Best wishes!

Su

--------------原始邮件--------------

发件人:"XianYang Qin "<xyqin@riken.jp>;
发送时间:2023 年 4 月 26 日(星期三) 上午 10:08
收件人:"yfurutani@jikei.ac.jp" <yfurutani@jikei.ac.jp>;"yonezawa.kento@ms.naist.jp" <yonezawa.kento@ms.naist.jp>;"nobutaka.shimizu@kek.jp" <nobutaka.shimizu@kek.jp>;"miyuki.murayama.aa@riken.jp" <miyuki.murayama.aa@riken.jp>;"Mikako Shirouzu" <mikako.shirouzu@riken.jp>;"y-yamano@kobepharma-u.ac.jp" <y-yamano@kobepharma-u.ac.jp>;"w.akimori5139@gmail.com" <w.akimori5139@gmail.com>;"Luc Gailhouste" <luc.gailhouste@riken.jp>;"rajanshrestha@gmail.com" <rajanshrestha@gmail.com>;"jkeillor@uottawa.ca" <jkeillor@uottawa.ca>;"fujiis.chem@tmd.ac.jp" <fujiis.chem@tmd.ac.jp>;"kage.chem@tmd.ac.jp" <kage.chem@tmd.ac.jp>;"tmasaki@jikei.ac.jp" <tmasaki@jikei.ac.jp>;"matsuuratomo@gmail.com" <matsuuratomo@gmail.com>;"Harukazu Suzuki" <harukazu.suzuki@riken.jp>;"dr.suting@smail.nju.edu.cn" <dr.suting@smail.nju.edu.cn>;"yudrnj@nju.edu.cn" <yudrnj@nju.edu.cn>;"NaoshiDohmae" <dohmae@riken.jp>;"Yali Xu" <yali.xu@riken.jp>;"shimizu.masahito.j1@f.gifu-u.ac.jp" <shimizu.masahito.j1@f.gifu-u.ac.jp>;"shirakami.yohei.t3@f.gifu-u.ac.jp" <shirakami.yohei.t3@f.gifu-u.ac.jp>;"rajanshrestha@ku.edu.np" <rajanshrestha@ku.edu.np>;

主题:Your response is NEEDED

Dear all,

The ACR-TG2 paper is currently undergoing the final quality check in Cell Death & Disease, and I need your confirmation on the authorships.

The most current author list is as follows:

Xian-Yang Qin, Yutaka Furutani, Kento Yonezawa, Nobutaka Shimizu, Miyuki Kato-Murayama, Mikako Shirouzu, Yali Xu, Yumiko Yamano, Akimori Wada, Luc Gailhouste, Rajan Shrestha, Masataka Takahashi, Jeffrey W. Keillor, Ting Su, Wenkui Yu, Shinya Fujii, Hiroyuki Kagechika, Naoshi Dohmae, Yohei Shirakami, Masahito Shimizu, Takahiro Masaki, Tomokazu Matsuura, Harukazu Suzuki

As requested by the journal, I have removed Kojima-sensei from the author list. Alternatively, a footnote has been added to the title to highlight that "This manuscript is dedicated to the memory of Dr. Soichi Kojima (1961-2019)".

Please reply to this email with your agreement on the authorship. I apologize for any inconvenience. You may simply reply with a single word. The most current version of the manuscript is attached at the end of this email.

Best regards,

Xian-Yang

Xian-Yang QIN, Ph.D.

Laboratory for Cellular Function Conversion Technology,

RIKEN Center for Integrative Medical Sciences

1-7-22 Suehiro, Tsurumi, Yokohama, Kanagawa 230-0045, Japan

Tel. +81-(0)50-3502-3731; Fax +81-45-503-9216

E-mail address: xyqin@riken.jp

From: cddisease@springernature.com <cddisease@springernature.com>
Sent: Tuesday, April 25, 2023 11:38 PM
To: XianYang Qin <xyqin@riken.jp>
Subject: CDDIS-22-1027RRR Initial Quality Check

Dear Dr Qin,

In checking in your manuscript submitted to Cell Death & Disease it has come to our attention that the

following must be addressed before we can proceed.

1. It has come to our attention that your most recent author list differs from the one in your original submission. Please request agreement from all authors including additions and deletions, these can be collected in the following way:

Email your co-authors with the final author list, and ask them to reply to your email confirming that they agree to the way it is presented. Once you have collected these replies, please combine all of the co-authors' email responses in one document and upload this file to your submission.

2. In checking your manuscript, we have noticed that the author list in your article file differs from your submission. Please could you ensure that all authors are included in both your article file and your online submission.

3. Please make sure you include a data availability statement in your Article file under its own subheading.

Your paper is now available for you to edit, you may access via the following link:

https://mts-cddis.nature.com/cgi-bin/main.plex?el=A5CA3XVP3D2BUIH7F5A9ftdobz29akaW4A60jLYOwQCwZ

(Press/Click on the above link to be automatically sent to the web page.)

Please make the correction(s) as specified above and resubmit your paper following the same steps as before.

If you have any questions please feel free to contact us.

Sincerely,

Editorial Office
Cell Death & Disease
cddisease@springernature.com

This email has been sent through the NPG Manuscript Tracking System NY-610A-NPG&MTS

Confidentiality Statement:

This e-mail is confidential and subject to copyright. Any unauthorised use or disclosure of its contents is prohibited. If you have received this email in error please notify our Manuscript Tracking System Helpdesk team at http://platformsupport.nature.com .

Details of the confidentiality and pre-publicity policy may be found here
http://www.nature.com/authors/policies/confidentiality.html

Privacy Policy | Update Profile

XianYang Qin

差出人: yudrnj <yudrnj@nju.edu.cn>
送信日時: 2023年4月26日水曜日 16:06
宛先: XianYang Qin

April 26, 2023

Dear Dr Qin

I agree with your suggestion.

Thank you.

wenkui

nanjing university

XianYang Qin

**差出人:**	fujchem@tmd.ac.jp は FUJII Shinya <fujiis.chem@tmd.ac.jp> の代理
**送信日時:**	2023年4月26日水曜日 11:47
**宛先:**	XianYang Qin
**件名:**	Re: Your response is NEEDED

Dear Qin-san,

I agree on the authorship.

Congratulations on the acceptance of this paper.
I am sure Kojima-sensei is pleased very much.

Best regards,

Shinya FUJII

藤井 晋也 -FUJII, Shinya---------------
東京医科歯科大学 生体材料工学研究所
薬化学分野
101-0062 東京都千代田区神田駿河台 2-3-10
Tel:03-5280-8034 Fax:03-5280-8127
fujiis.chem@tmd.ac.jp

----- Original Message -----
>> From: XianYang Qin <xyqin@riken.jp>
>> To: "yfurutani@jikei.ac.jp" <yfurutani@jikei.ac.jp>,"yonezawa.kento@ms.naist.jp"
<yonezawa.kento@ms.naist.jp>,"nobutaka.shimizu@kek.jp" <nobutaka.shimizu@kek.jp>
>> "miyuki.murayama.aa@riken.jp" <miyuki.murayama.aa@riken.jp>,Mikako Shirouzu
<mikako.shirouzu@riken.jp>,"y-yamano@kobepharma-u.ac.jp" <y-yamano@kobepharma-u.ac.jp>
>> "w.akimori5139@gmail.com" <w.akimori5139@gmail.com>,Luc Gailhouste
<luc.gailhouste@riken.jp>,"rajanshrestha@gmail.com" <rajanshrestha@gmail.com>
>> "jkeillor@uottawa.ca" <jkeillor@uottawa.ca>,"fujiis.chem@tmd.ac.jp"
<fujiis.chem@tmd.ac.jp>,"kage.chem@tmd.ac.jp" <kage.chem@tmd.ac.jp>
>> "tmasaki@jikei.ac.jp" <tmasaki@jikei.ac.jp>,"matsuuratomo@gmail.com"

<matsuuratomo@gmail.com>,Harukazu Suzuki <harukazu.suzuki@riken.jp>
>> "dr.suting@smail.nju.edu.cn" <dr.suting@smail.nju.edu.cn>,"yudrnj@nju.edu.cn" <yudrnj@nju.edu.cn>,Naoshi Dohmae <dohmae@riken.jp>
>> Yali Xu <yali.xu@riken.jp>,"shimizu.masahito.j1@f.gifu-u.ac.jp" <shimizu.masahito.j1@f.gifu-u.ac.jp>,"shirakami.yohei.t3@f.gifu-u.ac.jp" <shirakami.yohei.t3@f.gifu-u.ac.jp>
>> "rajanshrestha@ku.edu.np" <rajanshrestha@ku.edu.np>
>> Date: 2023-04-26 11:08:27
>> Subject: Your response is NEEDED
>>
>> Dear all,
>>
>> The ACR-TG2 paper is currently undergoing the final quality check in Cell Death & Disease, and I need your confirmation on the authorships.
>>
>> The most current author list is as follows:
>> Xian-Yang Qin, Yutaka Furutani, Kento Yonezawa, Nobutaka Shimizu,
>> Miyuki Kato-Murayama, Mikako Shirouzu, Yali Xu, Yumiko Yamano,
>> Akimori Wada, Luc Gailhouste, Rajan Shrestha, Masataka Takahashi,
>> Jeffrey W. Keillor, Ting Su, Wenkui Yu, Shinya Fujii, Hiroyuki
>> Kagechika, Naoshi Dohmae, Yohei Shirakami, Masahito Shimizu, Takahiro
>> Masaki, Tomokazu Matsuura, Harukazu Suzuki
>>
>> As requested by the journal, I have removed Kojima-sensei from the author list. Alternatively, a footnote has been added to the title to highlight that "This manuscript is dedicated to the memory of Dr. Soichi Kojima (1961-2019)".
>>
>> Please reply to this email with your agreement on the authorship. I apologize for any inconvenience. You may simply reply with a single word. The most current version of the manuscript is attached at the end of this email.
>>
>> Best regards,
>> Xian-Yang
>>
>> Xian-Yang QIN, Ph.D.
>> Laboratory for Cellular Function Conversion Technology, RIKEN Center
>> for Integrative Medical Sciences
>> 1-7-22 Suehiro, Tsurumi, Yokohama, Kanagawa 230-0045, Japan Tel.
>> +81-(0)50-3502-3731; Fax +81-45-503-9216 E-mail address:
>> xyqin@riken.jp<mailto:xyqin@riken.jp>
>>
>> From: cddisease@springernature.com <cddisease@springernature.com>

>> Sent: Tuesday, April 25, 2023 11:38 PM
>> To: XianYang Qin <xyqin@riken.jp>
>> Subject: CDDIS-22-1027RRR Initial Quality Check
>>
>> Dear Dr Qin,
>>
>> In checking in your manuscript submitted to Cell Death & Disease it has come to our attention that the following must be addressed before we can proceed.
>>
>> 1. It has come to our attention that your most recent author list differs from the one in your original submission. Please request agreement from all authors including additions and deletions, these can be collected in the following way:
>>
>> Email your co-authors with the final author list, and ask them to reply to your email confirming that they agree to the way it is presented. Once you have collected these replies, please combine all of the co-authors' email responses in one document and upload this file to your submission.
>>
>> 2. In checking your manuscript, we have noticed that the author list in your article file differs from your submission. Please could you ensure that all authors are included in both your article file and your online submission.
>>
>> 3. Please make sure you include a data availability statement in your Article file under its own subheading.
>>
>>
>> Your paper is now available for you to edit, you may access via the following link:
>>
>> https://mts-cddis.nature.com/cgi-bin/main.plex?el=A5CA3XVP3D2BUIH7F5A
>> 9ftdobz29akaW4A60jLYOwQCwZ
>>
>> (Press/Click on the above link to be automatically sent to the web
>> page.)
>>
>> Please make the correction(s) as specified above and resubmit your paper following the same steps as before.
>>
>> If you have any questions please feel free to contact us.
>>
>> Sincerely,
>>
>> Editorial Office

>> Cell Death & Disease
>> cddisease@springernature.com<mailto:cddisease@springernature.com>
>>
>>
>>
>>
>>
>> This email has been sent through the NPG Manuscript Tracking System
>> NY-610A-NPG&MTS
>>
>> Confidentiality Statement:
>>
>> This e-mail is confidential and subject to copyright. Any unauthorised use or disclosure of its contents is prohibited. If you have received this email in error please notify our Manuscript Tracking System Helpdesk team at http://platformsupport.nature.com .
>>
>> Details of the confidentiality and pre-publicity policy may be found
>> here http://www.nature.com/authors/policies/confidentiality.html
>>
>> Privacy Policy<http://www.nature.com/info/privacy.html> | Update
>> Profile<https://mts-cddis.nature.com>
>>
>>

XianYang Qin

**差出人:**	kagechem <kage.chem@tmd.ac.jp>
**送信日時:**	2023年4月26日水曜日 12:57
**宛先:**	XianYang Qin
**ＣＣ:**	kagechem
**件名:**	Re: Your response is NEEDED

I agree!
Hiroyuki Kagechika

2023/04/26 11:08、XianYang Qin <xyqin@riken.jp>のメール:

Dear all,

The ACR-TG2 paper is currently undergoing the final quality check in Cell Death & Disease, and I need your confirmation on the authorships.

The most current author list is as follows:
Xian-Yang Qin, Yutaka Furutani, Kento Yonezawa, Nobutaka Shimizu, Miyuki Kato-Murayama, Mikako Shirouzu, Yali Xu, Yumiko Yamano, Akimori Wada, Luc Gailhouste, Rajan Shrestha, Masataka Takahashi, Jeffrey W. Keillor, Ting Su, Wenkui Yu, Shinya Fujii, Hiroyuki Kagechika, Naoshi Dohmae, Yohei Shirakami, Masahito Shimizu, Takahiro Masaki, Tomokazu Matsuura, Harukazu Suzuki

As requested by the journal, I have removed Kojima-sensei from the author list. Alternatively, a footnote has been added to the title to highlight that "This manuscript is dedicated to the memory of Dr. Soichi Kojima (1961-2019)".

Please reply to this email with your agreement on the authorship. I apologize for any inconvenience. You may simply reply with a single word. The most current version of the manuscript is attached at the end of this email.

Best regards,
Xian-Yang

Xian-Yang QIN, Ph.D.
Laboratory for Cellular Function Conversion Technology,
RIKEN Center for Integrative Medical Sciences
1-7-22 Suehiro, Tsurumi, Yokohama, Kanagawa 230-0045, Japan
Tel. +81-(0)50-3502-3731; Fax +81-45-503-9216
E-mail address: xyqin@riken.jp

From: cddisease@springernature.com <cddisease@springernature.com>
Sent: Tuesday, April 25, 2023 11:38 PM
To: XianYang Qin <xyqin@riken.jp>
Subject: CDDIS-22-1027RRR Initial Quality Check

Dear Dr Qin,

In checking in your manuscript submitted to Cell Death & Disease it has come to our attention that

the following must be addressed before we can proceed.

1. It has come to our attention that your most recent author list differs from the one in your original submission. Please request agreement from all authors including additions and deletions, these can be collected in the following way:

Email your co-authors with the final author list, and ask them to reply to your email confirming that they agree to the way it is presented. Once you have collected these replies, please combine all of the co-authors' email responses in one document and upload this file to your submission.

2. In checking your manuscript, we have noticed that the author list in your article file differs from your submission. Please could you ensure that all authors are included in both your article file and your online submission.

3. Please make sure you include a data availability statement in your Article file under its own subheading.

Your paper is now available for you to edit, you may access via the following link:

https://mts-cddis.nature.com/cgi-bin/main.plex?el=A5CA3XVP3D2BUIH7F5A9ftdobz29akaW4A60jLYOwQCwZ

(Press/Click on the above link to be automatically sent to the web page.)

Please make the correction(s) as specified above and resubmit your paper following the same steps as before.

If you have any questions please feel free to contact us.

Sincerely,

Editorial Office
Cell Death & Disease
cddisease@springernature.com

This email has been sent through the NPG Manuscript Tracking System NY-610A-NPG&MTS

Confidentiality Statement:

This e-mail is confidential and subject to copyright. Any unauthorised use or disclosure of its contents is prohibited. If you have received this email in error please notify our Manuscript Tracking System Helpdesk team at http://platformsupport.nature.com .

Details of the confidentiality and pre-publicity policy may be found here http://www.nature.com/authors/policies/confidentiality.html

Privacy Policy | Update Profile

<4_ACR-TG2_CDDis_Manuscript_R4.docx>

****
影近　弘之
国立大学法人　東京医科歯科大学（TMDU）
生体材料工学研究所
教授（所長）
〒101-0062
東京都千代田区神田駿河台2-3-10
TEL: 03-5280-8032
FAX: 03-5280-8127
e-mail: kage.chem@tmd.ac.jp
URL: http://www.tmd.ac.jp/mri/omc/
****

XianYang Qin

差出人: Naoshi Dohmae
送信日時: 2023年4月26日水曜日 11:15
宛先: XianYang Qin
件名: Re: Your response is NEEDED

Dear Sir,

I agree with the change of the author.
Thanks,

Naoshi Dohmae

理研 CSRS 堂前
****
DOHMAE, Naoshi Ph,D. Deputy Division Director
& Unit Leader
dohmae@riken.jp
Biomolecular Characterization Unit,
Technology Platform Division,
RIKEN Center for Sustainable Resource Science
2-1, HIROSAWA, WAKO, SAITAMA. 351-0198 JAPAN

堂前 直 dohmae@riken.jp
理化学研究所 環境資源科学研究センター
技術基盤部門 副部門長
生命分子解析ユニット ユニットリーダー(兼)
〒351-0198 埼玉県和光市広沢 2-1 生物科学研究棟
TEL:048-467-9510 FAX:048-462-4704
http://www.riken.jp/BiomolChar/index.html

埼玉大学大学院理工学研究科 連携教授
http://www.molbiol.saitama-u.ac.jp/lab.html
****

差出人: XianYang Qin <xyqin@riken.jp>
送信日時: 2023 年 4 月 26 日 11:08
宛先: yfurutani@jikei.ac.jp <yfurutani@jikei.ac.jp>; yonezawa.kento@ms.naist.jp <yonezawa.kento@ms.naist.jp>; nobutaka.shimizu@kek.jp <nobutaka.shimizu@kek.jp>; miyuki.murayama.aa@riken.jp <miyuki.murayama.aa@riken.jp>; Mikako Shirouzu <mikako.shirouzu@riken.jp>; y-yamano@kobepharma-u.ac.jp <y-yamano@kobepharma-u.ac.jp>; w.akimori5139@gmail.com <w.akimori5139@gmail.com>; Luc Gailhouste <luc.gailhouste@riken.jp>; rajanshrestha@gmail.com <rajanshrestha@gmail.com>; jkeillor@uottawa.ca <jkeillor@uottawa.ca>; fujiis.chem@tmd.ac.jp <fujiis.chem@tmd.ac.jp>; kage.chem@tmd.ac.jp <kage.chem@tmd.ac.jp>; tmasaki@jikei.ac.jp <tmasaki@jikei.ac.jp>; matsuuratomo@gmail.com <matsuuratomo@gmail.com>; Harukazu Suzuki <harukazu.suzuki@riken.jp>; dr.suting@smail.nju.edu.cn <dr.suting@smail.nju.edu.cn>; yudrnj@nju.edu.cn <yudrnj@nju.edu.cn>; Naoshi Dohmae <dohmae@riken.jp>; Yali Xu <yali.xu@riken.jp>; shimizu.masahito.j1@f.gifu-u.ac.jp <shimizu.masahito.j1@f.gifu-u.ac.jp>;

shirakami.yohei.t3@f.gifu-u.ac.jp <shirakami.yohei.t3@f.gifu-u.ac.jp>; rajanshrestha@ku.edu.np <rajanshrestha@ku.edu.np>
件名: Your response is NEEDED

Dear all,

The ACR-TG2 paper is currently undergoing the final quality check in Cell Death & Disease, and I need your confirmation on the authorships.

The most current author list is as follows:
Xian-Yang Qin, Yutaka Furutani, Kento Yonezawa, Nobutaka Shimizu, Miyuki Kato-Murayama, Mikako Shirouzu, Yali Xu, Yumiko Yamano, Akimori Wada, Luc Gailhouste, Rajan Shrestha, Masataka Takahashi, Jeffrey W. Keillor, Ting Su, Wenkui Yu, Shinya Fujii, Hiroyuki Kagechika, Naoshi Dohmae, Yohei Shirakami, Masahito Shimizu, Takahiro Masaki, Tomokazu Matsuura, Harukazu Suzuki

As requested by the journal, I have removed Kojima-sensei from the author list. Alternatively, a footnote has been added to the title to highlight that "This manuscript is dedicated to the memory of Dr. Soichi Kojima (1961-2019)".

Please reply to this email with your agreement on the authorship. I apologize for any inconvenience. You may simply reply with a single word. The most current version of the manuscript is attached at the end of this email.

Best regards,
Xian-Yang

Xian-Yang QIN, Ph.D.
Laboratory for Cellular Function Conversion Technology,
RIKEN Center for Integrative Medical Sciences
1-7-22 Suehiro, Tsurumi, Yokohama, Kanagawa 230-0045, Japan
Tel. +81-(0)50-3502-3731; Fax +81-45-503-9216
E-mail address: xyqin@riken.jp

From: cddisease@springernature.com <cddisease@springernature.com>
Sent: Tuesday, April 25, 2023 11:38 PM
To: XianYang Qin <xyqin@riken.jp>
Subject: CDDIS-22-1027RRR Initial Quality Check

Dear Dr Qin,

In checking in your manuscript submitted to Cell Death & Disease it has come to our attention that the following must be addressed before we can proceed.

1. It has come to our attention that your most recent author list differs from the one in your original submission. Please request agreement from all authors including additions and deletions, these can be collected in the following way:

Email your co-authors with the final author list, and ask them to reply to your email confirming that they agree to the way it is presented. Once you have collected these replies, please combine all of the co-authors' email responses in one document and upload this file to your submission.

2. In checking your manuscript, we have noticed that the author list in your article file differs from your submission. Please could you ensure that all authors are included in both your article file and your online submission.

3. Please make sure you include a data availability statement in your Article file under its own subheading.

Your paper is now available for you to edit, you may access via the following link:

https://mts-cddis.nature.com/cgi-bin/main.plex?el=A5CA3XVP3D2BUIH7F5A9ftdobz29akaW4A60jLYOwQCwZ

(Press/Click on the above link to be automatically sent to the web page.)

Please make the correction(s) as specified above and resubmit your paper following the same steps as before.

If you have any questions please feel free to contact us.

Sincerely,

Editorial Office
Cell Death & Disease
cddisease@springernature.com

This email has been sent through the NPG Manuscript Tracking System NY-610A-NPG&MTS

Confidentiality Statement:

This e-mail is confidential and subject to copyright. Any unauthorised use or disclosure of its contents is prohibited. If you have received this email in error please notify our Manuscript Tracking System Helpdesk team at http://platformsupport.nature.com .
Details of the confidentiality and pre-publicity policy may be found here http://www.nature.com/authors/policies/confidentiality.html
Privacy Policy | Update Profile

XianYang Qin

**差出人:**	SHIRAKAMI Yohei <shirakami.yohei.t3@f.gifu-u.ac.jp>
**送信日時:**	2023年4月26日水曜日 11:57
**宛先:**	XianYang Qin
**件名:**	RE: Your response is NEEDED

秦先生

お世話になります。
著者確認いたしました。有難うございました。

岐阜大学　白上

From: XianYang Qin <xyqin@riken.jp>
Sent: Wednesday, April 26, 2023 11:08 AM
To: yfurutani@jikei.ac.jp; yonezawa.kento@ms.naist.jp; nobutaka.shimizu@kek.jp; miyuki.murayama.aa@riken.jp; Mikako Shirouzu <mikako.shirouzu@riken.jp>; y-yamano@kobepharma-u.ac.jp; w.akimori5139@gmail.com; Luc Gailhouste <luc.gailhouste@riken.jp>; rajanshrestha@gmail.com; jkeillor@uottawa.ca; fujiis.chem@tmd.ac.jp; kage.chem@tmd.ac.jp; tmasaki@jikei.ac.jp; matsuuratomo@gmail.com; Harukazu Suzuki <harukazu.suzuki@riken.jp>; dr.suting@smail.nju.edu.cn; yudrnj@nju.edu.cn; Naoshi Dohmae <dohmae@riken.jp>; Yali Xu <yali.xu@riken.jp>; SHIMIZU Masahito <shimizu.masahito.j1@f.gifu-u.ac.jp>; SHIRAKAMI Yohei <shirakami.yohei.t3@f.gifu-u.ac.jp>; rajanshrestha@ku.edu.np
Subject: Your response is NEEDED

Dear all,

The ACR-TG2 paper is currently undergoing the final quality check in Cell Death & Disease, and I need your confirmation on the authorships.

The most current author list is as follows:
Xian-Yang Qin, Yutaka Furutani, Kento Yonezawa, Nobutaka Shimizu, Miyuki Kato-Murayama, Mikako Shirouzu, Yali Xu, Yumiko Yamano, Akimori Wada, Luc Gailhouste, Rajan Shrestha, Masataka Takahashi, Jeffrey W. Keillor, Ting Su, Wenkui Yu, Shinya Fujii, Hiroyuki Kagechika, Naoshi Dohmae, Yohei Shirakami, Masahito Shimizu, Takahiro Masaki, Tomokazu Matsuura, Harukazu Suzuki

As requested by the journal, I have removed Kojima-sensei from the author list. Alternatively, a footnote has been added to the title to highlight that "This manuscript is dedicated to the memory of Dr. Soichi Kojima (1961-2019)".

Please reply to this email with your agreement on the authorship. I apologize for any inconvenience. You may simply reply with a single word. The most current version of the manuscript is attached at the end of this email.

Best regards,
Xian-Yang

Xian-Yang QIN, Ph.D.
Laboratory for Cellular Function Conversion Technology,
RIKEN Center for Integrative Medical Sciences
1-7-22 Suehiro, Tsurumi, Yokohama, Kanagawa 230-0045, Japan
Tel. +81-(0)50-3502-3731; Fax +81-45-503-9216

E-mail address: xyqin@riken.jp

From: cddisease@springernature.com <cddisease@springernature.com>
Sent: Tuesday, April 25, 2023 11:38 PM
To: XianYang Qin <xyqin@riken.jp>
Subject: CDDIS-22-1027RRR Initial Quality Check

Dear Dr Qin,

In checking in your manuscript submitted to Cell Death & Disease it has come to our attention that the following must be addressed before we can proceed.

1. It has come to our attention that your most recent author list differs from the one in your original submission. Please request agreement from all authors including additions and deletions, these can be collected in the following way:

Email your co-authors with the final author list, and ask them to reply to your email confirming that they agree to the way it is presented. Once you have collected these replies, please combine all of the co-authors' email responses in one document and upload this file to your submission.

2. In checking your manuscript, we have noticed that the author list in your article file differs from your submission. Please could you ensure that all authors are included in both your article file and your online submission.

3. Please make sure you include a data availability statement in your Article file under its own subheading.

Your paper is now available for you to edit, you may access via the following link:

https://mts-cddis.nature.com/cgi-bin/main.plex?el=A5CA3XVP3D2BUIH7F5A9ftdobz29akaW4A60jLYOwQCwZ

(Press/Click on the above link to be automatically sent to the web page.)

Please make the correction(s) as specified above and resubmit your paper following the same steps as before.

If you have any questions please feel free to contact us.

Sincerely,

Editorial Office
Cell Death & Disease
cddisease@springernature.com

This email has been sent through the NPG Manuscript Tracking System NY-610A-NPG&MTS

Confidentiality Statement:

This e-mail is confidential and subject to copyright. Any unauthorised use or disclosure of its contents is prohibited. If you have received this email in error please notify our Manuscript Tracking System Helpdesk team at http://platformsupport.nature.com .

Details of the confidentiality and pre-publicity policy may be found here http://www.nature.com/authors/policies/confidentiality.html

Privacy Policy | Update Profile

XianYang Qin

**差出人:**	SHIMIZU Masahito <shimizu.masahito.j1@f.gifu-u.ac.jp>
**送信日時:**	2023年4月26日水曜日 13:42
**宛先:**	XianYang Qin; yfurutani@jikei.ac.jp; yonezawa.kento@ms.naist.jp; nobutaka.shimizu@kek.jp; miyuki.murayama.aa@riken.jp; Mikako Shirouzu; y-yamano@kobepharma-u.ac.jp; w.akimori5139@gmail.com; Luc Gailhouste; rajanshrestha@gmail.com; jkeillor@uottawa.ca; fujiis.chem@tmd.ac.jp; kage.chem@tmd.ac.jp; tmasaki@jikei.ac.jp; matsuuratomo@gmail.com; Harukazu Suzuki; dr.suting@smail.nju.edu.cn; yudrnj@nju.edu.cn; Naoshi Dohmae; Yali Xu; SHIRAKAMI Yohei; rajanshrestha@ku.edu.np
**件名:**	Re: Your response is NEEDED

April 26, 2023

Dear Dr Qin,

I agree with your suggestion.
Thank you.

Masahito
Gifu University

送信元: XianYang Qin <xyqin@riken.jp>
日付: 2023年4月26日 水曜日 11:08
宛先: "yfurutani@jikei.ac.jp" <yfurutani@jikei.ac.jp>, "yonezawa.kento@ms.naist.jp" <yonezawa.kento@ms.naist.jp>, "nobutaka.shimizu@kek.jp" <nobutaka.shimizu@kek.jp>, "miyuki.murayama.aa@riken.jp" <miyuki.murayama.aa@riken.jp>, Mikako Shirouzu <mikako.shirouzu@riken.jp>, "y-yamano@kobepharma-u.ac.jp" <y-yamano@kobepharma-u.ac.jp>, "w.akimori5139@gmail.com" <w.akimori5139@gmail.com>, Luc Gailhouste <luc.gailhouste@riken.jp>, "rajanshrestha@gmail.com" <rajanshrestha@gmail.com>, "jkeillor@uottawa.ca" <jkeillor@uottawa.ca>, "fujiis.chem@tmd.ac.jp" <fujiis.chem@tmd.ac.jp>, "kage.chem@tmd.ac.jp" <kage.chem@tmd.ac.jp>, "tmasaki@jikei.ac.jp" <tmasaki@jikei.ac.jp>, "matsuuratomo@gmail.com" <matsuuratomo@gmail.com>, Harukazu Suzuki <harukazu.suzuki@riken.jp>, "dr.suting@smail.nju.edu.cn" <dr.suting@smail.nju.edu.cn>, "yudrnj@nju.edu.cn" <yudrnj@nju.edu.cn>, Naoshi Dohmae <dohmae@riken.jp>, Yali Xu <yali.xu@riken.jp>, SHIMIZU Masahito <shimizu.masahito.j1@f.gifu-u.ac.jp>, SHIRAKAMI Yohei <shirakami.yohei.t3@f.gifu-u.ac.jp>, "rajanshrestha@ku.edu.np" <rajanshrestha@ku.edu.np>
件名: Your response is NEEDED

Dear all,

The ACR-TG2 paper is currently undergoing the final quality check in Cell Death & Disease, and I need your confirmation on the authorships.

The most current author list is as follows:
Xian-Yang Qin, Yutaka Furutani, Kento Yonezawa, Nobutaka Shimizu, Miyuki Kato-Murayama, Mikako Shirouzu, Yali Xu, Yumiko Yamano, Akimori Wada, Luc Gailhouste, Rajan Shrestha, Masataka Takahashi, Jeffrey W. Keillor, Ting Su, Wenkui Yu, Shinya Fujii, Hiroyuki Kagechika, Naoshi Dohmae, Yohei Shirakami, Masahito Shimizu, Takahiro Masaki, Tomokazu Matsuura, Harukazu Suzuki

As requested by the journal, I have removed Kojima-sensei from the author list. Alternatively, a footnote has been added to the title to highlight that "This manuscript is dedicated to the memory of Dr. Soichi Kojima (1961-2019)".

Please reply to this email with your agreement on the authorship. I apologize for any inconvenience. You may simply reply with a single word. The most current version of the manuscript is attached at the end of this email.

Best regards,
Xian-Yang

Xian-Yang QIN, Ph.D.
Laboratory for Cellular Function Conversion Technology,
RIKEN Center for Integrative Medical Sciences
1-7-22 Suehiro, Tsurumi, Yokohama, Kanagawa 230-0045, Japan
Tel. +81-(0)50-3502-3731; Fax +81-45-503-9216
E-mail address: xyqin@riken.jp

From: cddisease@springernature.com <cddisease@springernature.com>
Sent: Tuesday, April 25, 2023 11:38 PM
To: XianYang Qin <xyqin@riken.jp>
Subject: CDDIS-22-1027RRR Initial Quality Check

Dear Dr Qin,

In checking in your manuscript submitted to Cell Death & Disease it has come to our attention that the following must be addressed before we can proceed.

1. It has come to our attention that your most recent author list differs from the one in your original submission. Please request agreement from all authors including additions and deletions, these can be collected in the following way:

Email your co-authors with the final author list, and ask them to reply to your email confirming that they agree to the way it is presented. Once you have collected these replies, please combine all of the co-authors' email responses in one document and upload this file to your submission.

2. In checking your manuscript, we have noticed that the author list in your article file differs from your submission. Please could you ensure that all authors are included in both your article file and your online submission.

3. Please make sure you include a data availability statement in your Article file under its own subheading.

Your paper is now available for you to edit, you may access via the following link:

https://mts-cddis.nature.com/cgi-bin/main.plex?el=A5CA3XVP3D2BUIH7F5A9ftdobz29akaW4A60jLYOwQCwZ

(Press/Click on the above link to be automatically sent to the web page.)

Please make the correction(s) as specified above and resubmit your paper following the same steps as before.

If you have any questions please feel free to contact us.

Sincerely,

Editorial Office
Cell Death & Disease
cddisease@springernature.com

This email has been sent through the NPG Manuscript Tracking System NY-610A-NPG&MTS

Confidentiality Statement:

This e-mail is confidential and subject to copyright. Any unauthorised use or disclosure of its contents is prohibited. If you have received this email in error please notify our Manuscript Tracking System Helpdesk team at http://platformsupport.nature.com .

Details of the confidentiality and pre-publicity policy may be found here http://www.nature.com/authors/policies/confidentiality.html

Privacy Policy | Update Profile

差出人: 政木 隆博 <tmasaki@jikei.ac.jp>
送信日時: 2023年4月26日水曜日 11:14
宛先: XianYang Qin
件名: Re: Your response is NEEDED

秦先生、

ご連絡を頂き有難うございます。
論文のアクセプトおめでとうございます！
共著者にして頂き誠に有難うございます。
オーサーシップに関しまして問題ないと思います。
今後ともどうぞよろしくお願い申し上げます。

政木隆博　拝

> 2023/04/26 11:08、XianYang Qin <xyqin@riken.jp>のメール:
>
> Dear all,
>　The ACR-TG2 paper is currently undergoing the final quality check in Cell Death & Disease, and I need your confirmation on the authorships.
>　The most current author list is as follows:
> Xian-Yang Qin, Yutaka Furutani, Kento Yonezawa, Nobutaka Shimizu,
> Miyuki Kato-Murayama, Mikako Shirouzu, Yali Xu, Yumiko Yamano, Akimori Wada, Luc Gailhouste, Rajan Shrestha, Masataka Takahashi, Jeffrey W. Keillor, Ting Su, Wenkui Yu, Shinya Fujii, Hiroyuki Kagechika, Naoshi Dohmae, Yohei Shirakami, Masahito Shimizu, Takahiro Masaki, Tomokazu Matsuura, Harukazu Suzuki　As requested by the journal, I have removed Kojima-sensei from the author list. Alternatively, a footnote has been added to the title to highlight that "This manuscript is dedicated to the memory of Dr. Soichi Kojima (1961-2019)".
>　Please reply to this email with your agreement on the authorship. I apologize for any inconvenience. You may simply reply with a single word. The most current version of the manuscript is attached at the end of this email.
>　Best regards,
> Xian-Yang
>　Xian-Yang QIN, Ph.D.
> Laboratory for Cellular Function Conversion Technology, RIKEN Center
> for Integrative Medical Sciences
> 1-7-22 Suehiro, Tsurumi, Yokohama, Kanagawa 230-0045, Japan Tel.
> +81-(0)50-3502-3731; Fax +81-45-503-9216 E-mail address:

> xyqin@riken.jp
> From: cddisease@springernature.com <cddisease@springernature.com>
> Sent: Tuesday, April 25, 2023 11:38 PM
> To: XianYang Qin <xyqin@riken.jp>
> Subject: CDDIS-22-1027RRR Initial Quality Check Dear Dr Qin,
>
> In checking in your manuscript submitted to Cell Death & Disease it has come to our attention that the following must be addressed before we can proceed.
>
> 1. It has come to our attention that your most recent author list differs from the one in your original submission. Please request agreement from all authors including additions and deletions, these can be collected in the following way:
>
> Email your co-authors with the final author list, and ask them to reply to your email confirming that they agree to the way it is presented. Once you have collected these replies, please combine all of the co-authors' email responses in one document and upload this file to your submission.
>
> 2. In checking your manuscript, we have noticed that the author list in your article file differs from your submission. Please could you ensure that all authors are included in both your article file and your online submission.
>
> 3. Please make sure you include a data availability statement in your Article file under its own subheading.
>
>
> Your paper is now available for you to edit, you may access via the following link:
>
> https://mts-cddis.nature.com/cgi-bin/main.plex?el=A5CA3XVP3D2BUIH7F5A9
> ftdobz29akaW4A60jLYOwQCwZ
>
> (Press/Click on the above link to be automatically sent to the web
> page.)
>
> Please make the correction(s) as specified above and resubmit your paper following the same steps as before.
>
> If you have any questions please feel free to contact us.
>
> Sincerely,
>
> Editorial Office

> Cell Death & Disease
> cddisease@springernature.com
>
>
>
>
> This email has been sent through the NPG Manuscript Tracking System
> NY-610A-NPG&MTS
>
> Confidentiality Statement:
>
> This e-mail is confidential and subject to copyright. Any unauthorised use or disclosure of its contents is prohibited. If you have received this email in error please notify our Manuscript Tracking System Helpdesk team at http://platformsupport.nature.com .
> Details of the confidentiality and pre-publicity policy may be found
> here http://www.nature.com/authors/policies/confidentiality.html
> Privacy Policy | Update Profile<4_ACR-TG2_CDDis_Manuscript_R4.docx>

XianYang Qin

差出人: Tomo Matsuura <matsuuratomo@gmail.com>
送信日時: 2023年4月27日木曜日 9:52
宛先: XianYang Qin
件名: Re: Your response is NEEDED

Dear Dr XY Qin,

Thank you for your e-mail.

I agree.

Tomokazu Matsuura

2023 年 4 月 26 日(水) 11:08 XianYang Qin <xyqin@riken.jp>:

> Dear all,
>
> The ACR-TG2 paper is currently undergoing the final quality check in Cell Death & Disease, and I need your confirmation on the authorships.
>
> The most current author list is as follows:
>
> Xian-Yang Qin, Yutaka Furutani, Kento Yonezawa, Nobutaka Shimizu, Miyuki Kato-Murayama, Mikako Shirouzu, Yali Xu, Yumiko Yamano, Akimori Wada, Luc Gailhouste, Rajan Shrestha, Masataka Takahashi, Jeffrey W. Keillor, Ting Su, Wenkui Yu, Shinya Fujii, Hiroyuki Kagechika, Naoshi Dohmae, Yohei Shirakami, Masahito Shimizu, Takahiro Masaki, Tomokazu Matsuura, Harukazu Suzuki
>
> As requested by the journal, I have removed Kojima-sensei from the author list. Alternatively, a footnote has been added to the title to highlight that "This manuscript is dedicated to the memory of Dr. Soichi Kojima (1961-2019)".
>
> Please reply to this email with your agreement on the authorship. I apologize for any inconvenience. You may simply reply with a single word. The most current version of the manuscript is attached at the end of this email.
>
> Best regards,

Xian-Yang

Xian-Yang QIN, Ph.D.

Laboratory for Cellular Function Conversion Technology,

RIKEN Center for Integrative Medical Sciences

1-7-22 Suehiro, Tsurumi, Yokohama, Kanagawa 230-0045, Japan

Tel. +81-(0)50-3502-3731; Fax +81-45-503-9216

E-mail address: xyqin@riken.jp

From: cddisease@springernature.com <cddisease@springernature.com>
Sent: Tuesday, April 25, 2023 11:38 PM
To: XianYang Qin <xyqin@riken.jp>
Subject: CDDIS-22-1027RRR Initial Quality Check

Dear Dr Qin,

In checking in your manuscript submitted to Cell Death & Disease it has come to our attention that the following must be addressed before we can proceed.

1. It has come to our attention that your most recent author list differs from the one in your original submission. Please request agreement from all authors including additions and deletions, these can be collected in the following way:

Email your co-authors with the final author list, and ask them to reply to your email confirming that they agree to the way it is presented. Once you have collected these replies, please combine all of the co-authors' email responses in one document and upload this file to your submission.

2. In checking your manuscript, we have noticed that the author list in your article file differs from your submission. Please could you ensure that all authors are included in both your article file and your online submission.

3. Please make sure you include a data availability statement in your Article file under its own subheading.

Your paper is now available for you to edit, you may access via the following link:

https://mts-cddis.nature.com/cgi-

bin/main.plex?el=A5CA3XVP3D2BUIH7F5A9ftdobz29akaW4A60jLYOwQCwZ

(Press/Click on the above link to be automatically sent to the web page.)

Please make the correction(s) as specified above and resubmit your paper following the same steps as before.

If you have any questions please feel free to contact us.

Sincerely,

Editorial Office
Cell Death & Disease
cddisease@springernature.com

This email has been sent through the NPG Manuscript Tracking System NY-610A-NPG&MTS

Confidentiality Statement:

This e-mail is confidential and subject to copyright. Any unauthorised use or disclosure of its contents is prohibited. If you have received this email in error please notify our Manuscript Tracking System Helpdesk team at http://platformsupport.nature.com .

Details of the confidentiality and pre-publicity policy may be found here http://www.nature.com/authors/policies/confidentiality.html

Privacy Policy | Update Profile

--
公益財団法人佐々木研究所附属
湘南健診センター
センター長　松浦知和
（東京慈恵会医科大学 客員教授）
電話 0463-21-3811（代表）
matsuuratomo@gmail.com

XianYang Qin

**差出人:**	Harukazu Suzuki
**送信日時:**	2023年4月26日水曜日 11:14
**宛先:**	XianYang Qin; yfurutani@jikei.ac.jp; yonezawa.kento@ms.naist.jp; nobutaka.shimizu@kek.jp; miyuki.murayama.aa@riken.jp; Mikako Shirouzu; y-yamano@kobepharma-u.ac.jp; w.akimori5139@gmail.com; Luc Gailhouste; rajanshrestha@gmail.com; jkeillor@uottawa.ca; fujiis.chem@tmd.ac.jp; kage.chem@tmd.ac.jp; tmasaki@jikei.ac.jp; matsuuratomo@gmail.com; dr.suting@smail.nju.edu.cn; yudrnj@nju.edu.cn; Naoshi Dohmae; Yali Xu; shimizu.masahito.j1@f.gifu-u.ac.jp; shirakami.yohei.t3@f.gifu-u.ac.jp; rajanshrestha@ku.edu.np
**件名:**	RE: Your response is NEEDED

I am fine.

Harukazu

From: XianYang Qin <xyqin@riken.jp>
Sent: Wednesday, April 26, 2023 11:08 AM
To: yfurutani@jikei.ac.jp; yonezawa.kento@ms.naist.jp; nobutaka.shimizu@kek.jp; miyuki.murayama.aa@riken.jp; Mikako Shirouzu <mikako.shirouzu@riken.jp>; y-yamano@kobepharma-u.ac.jp; w.akimori5139@gmail.com; Luc Gailhouste <luc.gailhouste@riken.jp>; rajanshrestha@gmail.com; jkeillor@uottawa.ca; fujiis.chem@tmd.ac.jp; kage.chem@tmd.ac.jp; tmasaki@jikei.ac.jp; matsuuratomo@gmail.com; Harukazu Suzuki <harukazu.suzuki@riken.jp>; dr.suting@smail.nju.edu.cn; yudrnj@nju.edu.cn; Naoshi Dohmae <dohmae@riken.jp>; Yali Xu <yali.xu@riken.jp>; shimizu.masahito.j1@f.gifu-u.ac.jp; shirakami.yohei.t3@f.gifu-u.ac.jp; rajanshrestha@ku.edu.np
Subject: Your response is NEEDED

Dear all,

The ACR-TG2 paper is currently undergoing the final quality check in Cell Death & Disease, and I need your confirmation on the authorships.

The most current author list is as follows:
Xian-Yang Qin, Yutaka Furutani, Kento Yonezawa, Nobutaka Shimizu, Miyuki Kato-Murayama, Mikako Shirouzu, Yali Xu, Yumiko Yamano, Akimori Wada, Luc Gailhouste, Rajan Shrestha, Masataka Takahashi, Jeffrey W. Keillor, Ting Su, Wenkui Yu, Shinya Fujii, Hiroyuki Kagechika, Naoshi Dohmae, Yohei Shirakami, Masahito Shimizu, Takahiro Masaki, Tomokazu Matsuura, Harukazu Suzuki

As requested by the journal, I have removed Kojima-sensei from the author list. Alternatively, a footnote has been added to the title to highlight that "This manuscript is dedicated to the memory of Dr. Soichi Kojima (1961-2019)".

Please reply to this email with your agreement on the authorship. I apologize for any inconvenience. You may simply reply with a single word. The most current version of the manuscript is attached at the end of this email.

Best regards,
Xian-Yang

Xian-Yang QIN, Ph.D.

Laboratory for Cellular Function Conversion Technology,
RIKEN Center for Integrative Medical Sciences
1-7-22 Suehiro, Tsurumi, Yokohama, Kanagawa 230-0045, Japan
Tel. +81-(0)50-3502-3731; Fax +81-45-503-9216
E-mail address: xyqin@riken.jp

From: cddisease@springernature.com <cddisease@springernature.com>
Sent: Tuesday, April 25, 2023 11:38 PM
To: XianYang Qin <xyqin@riken.jp>
Subject: CDDIS-22-1027RRR Initial Quality Check

Dear Dr Qin,

In checking in your manuscript submitted to Cell Death & Disease it has come to our attention that the following must be addressed before we can proceed.

1. It has come to our attention that your most recent author list differs from the one in your original submission. Please request agreement from all authors including additions and deletions, these can be collected in the following way:

Email your co-authors with the final author list, and ask them to reply to your email confirming that they agree to the way it is presented. Once you have collected these replies, please combine all of the co-authors' email responses in one document and upload this file to your submission.

2. In checking your manuscript, we have noticed that the author list in your article file differs from your submission. Please could you ensure that all authors are included in both your article file and your online submission.

3. Please make sure you include a data availability statement in your Article file under its own subheading.

Your paper is now available for you to edit, you may access via the following link:

https://mts-cddis.nature.com/cgi-bin/main.plex?el=A5CA3XVP3D2BUIH7F5A9ftdobz29akaW4A60jLYOwQCwZ

(Press/Click on the above link to be automatically sent to the web page.)

Please make the correction(s) as specified above and resubmit your paper following the same steps as before.

If you have any questions please feel free to contact us.

Sincerely,

Editorial Office
Cell Death & Disease
cddisease@springernature.com

This email has been sent through the NPG Manuscript Tracking System NY-610A-NPG&MTS

Confidentiality Statement:

This e-mail is confidential and subject to copyright. Any unauthorised use or disclosure of its contents is prohibited. If you have received this email in error please notify our Manuscript Tracking System Helpdesk team at *http://platformsupport.nature.com* .

Details of the confidentiality and pre-publicity policy may be found here
http://www.nature.com/authors/policies/confidentiality.html

Privacy Policy | Update Profile
